# Accurate structure prediction of biomolecular interactions with AlphaFold 3

**DOI:** 10.1038/s41586-024-07487-w

**Published:** 2024-05-08

**Authors:** Josh Abramson, Jonas Adler, Jack Dunger, Richard Evans, Tim Green, Alexander Pritzel, Olaf Ronneberger, Lindsay Willmore, Andrew J. Ballard, Joshua Bambrick, Sebastian W. Bodenstein, David A. Evans, Chia-Chun Hung, Michael O’Neill, David Reiman, Kathryn Tunyasuvunakool, Zachary Wu, Akvilė Žemgulytė, Eirini Arvaniti, Charles Beattie, Ottavia Bertolli, Alex Bridgland, Alexey Cherepanov, Miles Congreve, Alexander I. Cowen-Rivers, Andrew Cowie, Michael Figurnov, Fabian B. Fuchs, Hannah Gladman, Rishub Jain, Yousuf A. Khan, Caroline M. R. Low, Kuba Perlin, Anna Potapenko, Pascal Savy, Sukhdeep Singh, Adrian Stecula, Ashok Thillaisundaram, Catherine Tong, Sergei Yakneen, Ellen D. Zhong, Michal Zielinski, Augustin Žídek, Victor Bapst, Pushmeet Kohli, Max Jaderberg, Demis Hassabis, John M. Jumper

**Affiliations:** 1 Core Contributor, Google DeepMind, London, UK; 2 Core Contributor, Isomorphic Labs, London, UK; 3 Google DeepMind, London, UK; 4 Isomorphic Labs, London, UK; 5 Department of Molecular and Cellular Physiology, Stanford University, Stanford, CA USA; 6 Department of Computer Science, Princeton University, Princeton, NJ USA

**Keywords:** Protein structure predictions, Structural biology, Machine learning, Drug discovery

## Abstract

The introduction of AlphaFold 2^1^ has spurred a revolution in modelling the structure of proteins and their interactions, enabling a huge range of applications in protein modelling and design^2–6^. Here we describe our AlphaFold 3 model with a substantially updated diffusion-based architecture that is capable of predicting the joint structure of complexes including proteins, nucleic acids, small molecules, ions and modified residues. The new AlphaFold model demonstrates substantially improved accuracy over many previous specialized tools: far greater accuracy for protein–ligand interactions compared with state-of-the-art docking tools, much higher accuracy for protein–nucleic acid interactions compared with nucleic-acid-specific predictors and substantially higher antibody–antigen prediction accuracy compared with AlphaFold-Multimer v.2.3^7,8^. Together, these results show that high-accuracy modelling across biomolecular space is possible within a single unified deep-learning framework.

## Abstract (web summary)

AlphaFold 3 has a substantially updated architecture that is capable of predicting the joint structure of complexes including proteins, nucleic acids, small molecules, ions and modified residues with greatly improved accuracy over many previous specialized tools.

## Main

Accurate models of biological complexes are critical to our understanding of cellular functions and for the rational design of therapeutics^2–4,9^. Enormous progress has been achieved in protein structure prediction with the development of AlphaFold^1^, and the field has grown tremendously with a number of later methods that build on the ideas and techniques of AlphaFold 2 (AF2)^10–12^. Almost immediately after AlphaFold became available, it was shown that simple input modifications would enable surprisingly accurate protein interaction predictions^13–15^ and that training AF2 specifically for protein interaction prediction yielded a highly accurate system^7^.

These successes lead to the question of whether it is possible to accurately predict the structure of complexes containing a much wider range of biomolecules, including ligands, ions, nucleic acids and modified residues, within a deep-learning framework. A wide range of predictors for various specific interaction types has been developed^16–28^, as well as one generalist method developed concurrently with the present work^29^, but the accuracy of such deep-learning attempts has been mixed and often below that of physics-inspired methods^30,31^. Almost all of these methods are also highly specialized to particular interaction types and cannot predict the structure of general biomolecular complexes containing many types of entities.

**Fig. 1:**
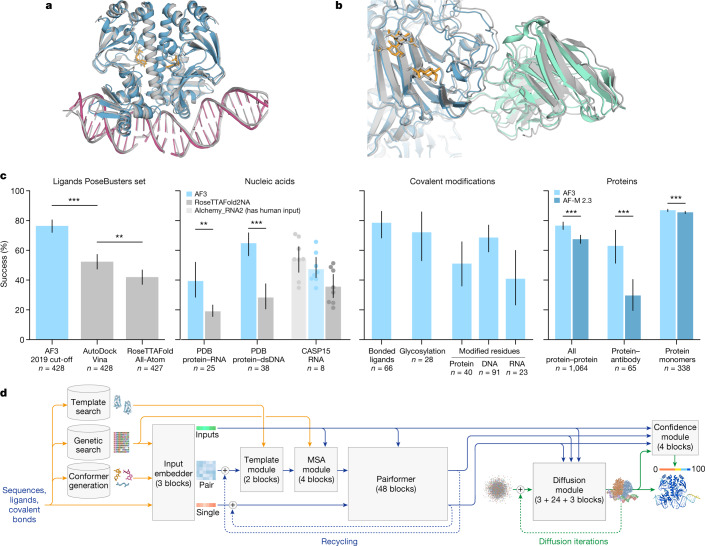
AF3 accurately predicts structures across biomolecular complexes. **a**,**b**, Example structures predicted using AF3. **a**, Bacterial CRP/FNR family transcriptional regulator protein bound to DNA and cGMP (PDB 7PZB; full-complex LDDT^47^, 82.8; global distance test (GDT)^48^, 90.1). **b**, Human coronavirus OC43 spike protein, 4,665 residues, heavily glycosylated and bound by neutralizing antibodies (PDB 7PNM; full-complex LDDT, 83.0; GDT, 83.1). **c**, AF3 performance on PoseBusters (v.1, August 2023 release), our recent PDB evaluation set and CASP15 RNA. Metrics are as follows: percentage of pocket-aligned ligand r.m.s.d. < 2 Å for ligands and covalent modifications; interface LDDT for protein–nucleic acid complexes; LDDT for nucleic acid and protein monomers; and percentage DockQ > 0.23 for protein–protein and protein–antibody interfaces. All scores are reported from the top confidence-ranked sample out of five model seeds (each with five diffusion samples), except for protein–antibody scores, which were ranked across 1,000 model seeds for both models (each AF3 seed with five diffusion samples). Sampling and ranking details are provided in the Methods. For ligands, *n* indicates the number of targets; for nucleic acids, *n* indicates the number of structures; for modifications, *n* indicates the number of clusters; and for proteins, *n* indicates the number of clusters. The bar height indicates the mean; error bars indicate exact binomial distribution 95% confidence intervals for PoseBusters and by 10,000 bootstrap resamples for all others. Significance levels were calculated using two-sided Fisher’s exact tests for PoseBusters and using two-sided Wilcoxon signed-rank tests for all others; ***P < 0.001, **P < 0.01. Exact *P* values (from left to right) are as follows: 2.27 × 10^−13^, 2.57 × 10^−3^, 2.78 × 10^−3^, 7.28 × 10^−12^, 1.81 × 10^−18^, 6.54 × 10^−5^ and 1.74 × 10^−34^. AF-M 2.3, AlphaFold-Multimer v.2.3; dsDNA, double-stranded DNA. **d**, AF3 architecture for inference. The rectangles represent processing modules and the arrows show the data flow. Yellow, input data; blue, abstract network activations; green, output data. The coloured balls represent physical atom coordinates.

Here we present AlphaFold 3 (AF3)—a model that is capable of high-accuracy prediction of complexes containing nearly all molecular types present in the Protein Data Bank^32^ (PDB) (Fig. 1a,b). In all but one category, it achieves a substantially higher performance than strong methods that specialize in just the given task (Fig. 1c and Extended Data Table 1), including higher accuracy at protein structure and the structure of protein–protein interactions.

**Fig. 2:**
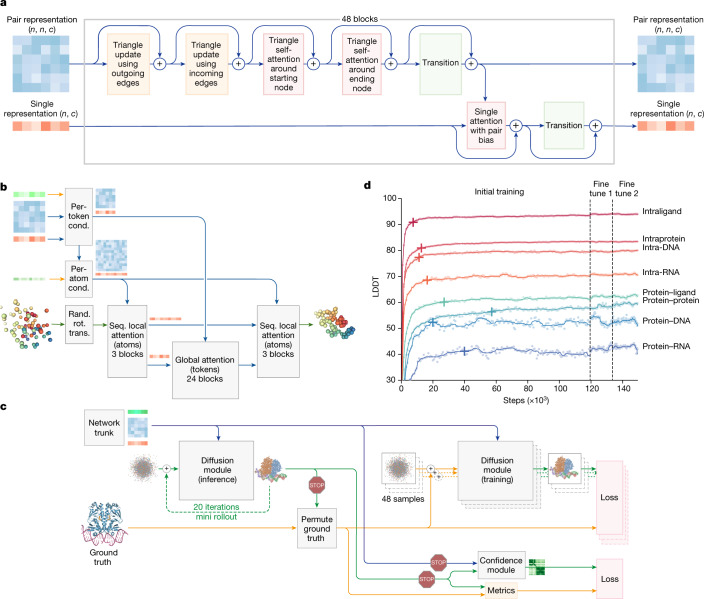
Architectural and training details. **a**, The pairformer module. Input and output: pair representation with dimension (*n*, *n*, *c*) and single representation with dimension (*n*, *c*). *n* is the number of tokens (polymer residues and atoms); *c* is the number of channels (128 for the pair representation, 384 for the single representation). Each of the 48 blocks has an independent set of trainable parameters. **b**, The diffusion module. Input: coarse arrays depict per-token representations (green, inputs; blue, pair; red, single). Fine arrays depict per-atom representations. The coloured balls represent physical atom coordinates. Cond., conditioning; rand. rot. trans., random rotation and translation; seq., sequence. **c**, The training set-up (distogram head omitted) starting from the end of the network trunk. The coloured arrays show activations from the network trunk (green, inputs; blue, pair; red, single). The blue arrows show abstract activation arrays. The yellow arrows show ground-truth data. The green arrows show predicted data. The stop sign represents stopping of the gradient. Both depicted diffusion modules share weights. **d**, Training curves for initial training and fine-tuning stages, showing the LDDT on our evaluation set as a function of optimizer steps. The scatter plot shows the raw datapoints and the lines show the smoothed performance using a median filter with a kernel width of nine datapoints. The crosses mark the point at which the smoothed performance reaches 97% of its initial training maximum.

This is achieved by a substantial evolution of the AF2 architecture and training procedure (Fig. 1d) both to accommodate more general chemical structures and to improve the data efficiency of learning. The system reduces the amount of multiple-sequence alignment (MSA) processing by replacing the AF2 evoformer with the simpler pairformer module (Fig. 2a). Furthermore it directly predicts the raw atom coordinates with a diffusion module, replacing the AF2 structure module that operated on amino-acid-specific frames and side-chain torsion angles (Fig. 2b). The multiscale nature of the diffusion process (low noise levels induce the network to improve local structure) also enable us to eliminate stereochemical losses and most special handling of bonding patterns in the network, easily accommodating arbitrary chemical components.

## Network architecture and training

The overall structure of AF3 (Fig. 1d and Supplementary Methods 3) echoes that of AF2, with a large trunk evolving a pairwise representation of the chemical complex followed by a structure module that uses the pairwise representation to generate explicit atomic positions, but there are large differences in each major component. These modifications were driven both by the need to accommodate a wide range of chemical entities without excessive special casing and by observations of AF2 performance with different modifications. Within the trunk, MSA processing is substantially de-emphasized, with a much smaller and simpler MSA embedding block (Supplementary Methods 3.3). Compared with the original evoformer from AF2, the number of blocks is reduced to four, the processing of the MSA representation uses an inexpensive pair-weighted averaging and only the pair representation is used for later processing steps. The ‘pairformer’ (Fig. 2a and Supplementary Methods 3.6) replaces the evoformer of AF2 as the dominant processing block. It operates only on the pair representation and the single representation; the MSA representation is not retained and all information passes through the pair representation. The pair processing and the number of blocks (48) is largely unchanged from AF2. The resulting pair and single representation together with the input representation are passed to the new diffusion module (Fig. 2b) that replaces the structure module of AF2.

The diffusion module (Fig. 2b and Supplementary Methods 3.7) operates directly on raw atom coordinates, and on a coarse abstract token representation, without rotational frames or any equivariant processing. We had observed in AF2 that removing most of the complexity of the structure module had only a modest effect on the prediction accuracy, and maintaining the backbone frame and side-chain torsion representation add quite a bit of complexity for general molecular graphs. Similarly AF2 required carefully tuned stereochemical violation penalties during training to enforce chemical plausibility of the resulting structures. We use a relatively standard diffusion approach^33^ in which the diffusion model is trained to receive ‘noised’ atomic coordinates and then predict the true coordinates. This task requires the network to learn protein structure at a variety of length scales, whereby the denoising task at small noise emphasizes understanding very local stereochemistry and the denoising task at high noise emphasizes the large-scale structure of the system. At the inference time, random noise is sampled and then recurrently denoised to produce a final structure. Importantly, this is a generative training procedure that produces a distribution of answers. This means that, for each answer, the local structure will be sharply defined (for example, side-chain bond geometry) even when the network is uncertain about the positions. For this reason, we are able to avoid both torsion-based parametrizations of the residues and violation losses on the structure, while handling the full complexity of general ligands. Similarly to some recent work^34^, we find that no invariance or equivariance with respect to global rotations and translation of the molecule are required in the architecture and we therefore omit them to simplify the machine learning architecture.

**Extended Data Fig. 1:**
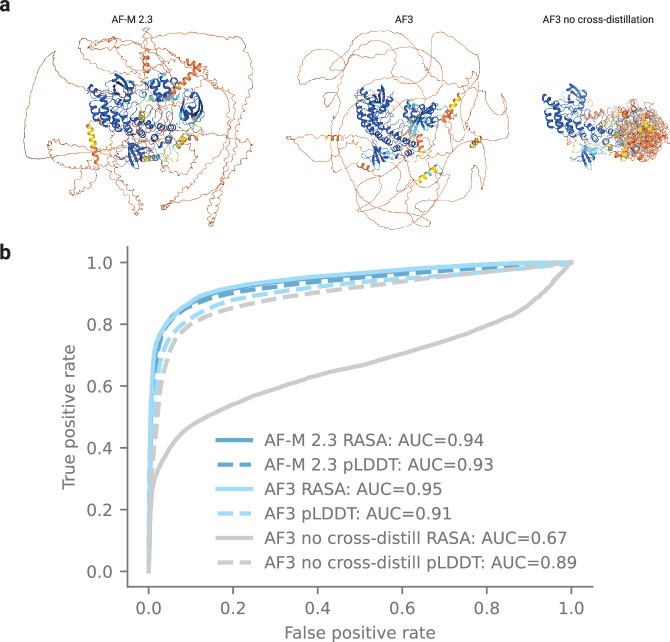
Disordered region prediction. **a**, Example prediction for a disordered protein from AlphaFoldMultimer v2.3, AlphaFold 3, and AlphaFold 3 trained without the disordered protein PDB cross distillation set. Protein is DP02376 from the CAID 2 (Critical Assessment of protein Intrinsic Disorder prediction) set. Predictions coloured by pLDDT (orange: pLDDT <= 50, yellow: 50 < pLDDT <= 70, light blue: 70 < pLDDT <= 90, and dark blue: 90 <= pLDDT < 100). **b**, Predictions of disorder across residues in proteins in the CAID 2 set, which are also low homology to the AF3 training set. Prediction methods include RASA (relative accessible surface area) and pLDDT (N = 151 proteins; 46,093 residues).

The use of a generative diffusion approach comes with some technical challenges that we needed to address. The biggest issue is that generative models are prone to hallucination^35^, whereby the model may invent plausible-looking structure even in unstructured regions. To counteract this effect, we use a cross-distillation method in which we enrich the training data with structures predicted by AlphaFold-Multimer (v.2.3)^7,8^. In these structures, unstructured regions are typically represented by long extended loops instead of compact structures, and training on them ‘teaches’ AF3 to mimic this behaviour. This cross-distillation greatly reduced the hallucination behaviour of AF3 (Extended Data Fig. 1 for disorder prediction results on the CAID 2^36^ benchmark set).

We also developed confidence measures that predict the atom-level and pairwise errors in our final structures. In AF2, this was done directly by regressing the error in the output of the structure module during training. However, this procedure is not applicable to diffusion training, as only a single step of the diffusion is trained instead of a full-structure generation (Fig. 2c). To remedy this, we developed a diffusion ‘rollout’ procedure for the full-structure prediction generation during training (using a larger step size than normal; Fig. 2c (mini-rollout)). This predicted structure is then used to permute the symmetric ground-truth chains and ligands, and to compute the performance metrics to train the confidence head. The confidence head uses the pairwise representation to predict a modified local distance difference test (pLDDT) and a predicted aligned error (PAE) matrix as in AF2, as well as a distance error matrix (PDE), which is the error in the distance matrix of the predicted structure as compared to the true structure (details are provided in Supplementary Methods 4.3).

**Extended Data Fig. 2:**
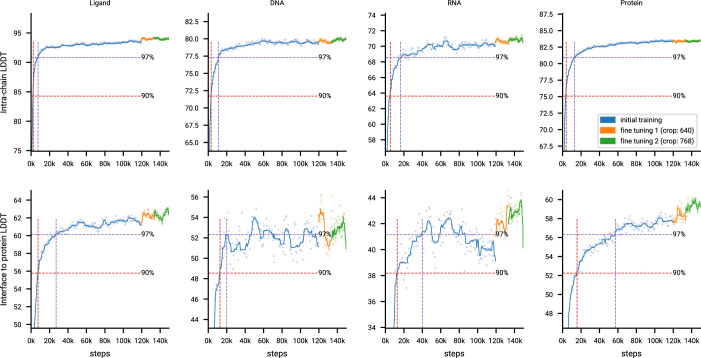
Accuracy across training. Training curves for initial training and fine tuning showing LDDT (local distance difference test) on our evaluation set as a function of optimizer steps. One optimizer step uses a mini batch of 256 trunk samples and during initial training 256 * 48 = 12,288 diffusion samples. For fine tuning the number of diffusion samples is reduced to 256 * 32 = 8,192. The scatter plot shows the raw data points and the lines show the smoothed performance using a median filter with a kernel width of 9 data points. The dashed lines mark the points where the smoothed performance passes 90% and 97% of the initial training maximum for the first time.

Figure 2d shows that, during initial training, the model learns quickly to predict the local structures (all intrachain metrics go up quickly and reach 97% of the maximum performance within the first 20,000 training steps), while the model needs considerably longer to learn the global constellation (the interface metrics go up slowly and protein–protein interface LDDT passes the 97% bar only after 60,000 steps). During AF3 development, we observed that some model abilities topped out relatively early and started to decline (most likely due to overfitting to the limited number of training samples for this capability), while other abilities were still undertrained. We addressed this by increasing or decreasing the sampling probability for the corresponding training sets (Supplementary Methods 2.5.1) and by performing early stopping using a weighted average of all of the above metrics and some additional metrics to select the best model checkpoint (Supplementary Table 7). The fine-tuning stages with the larger crop sizes improve the model on all metrics with an especially high uplift on protein–protein interfaces (Extended Data Fig. 2).

## Accuracy across complex types

**Fig. 3:**
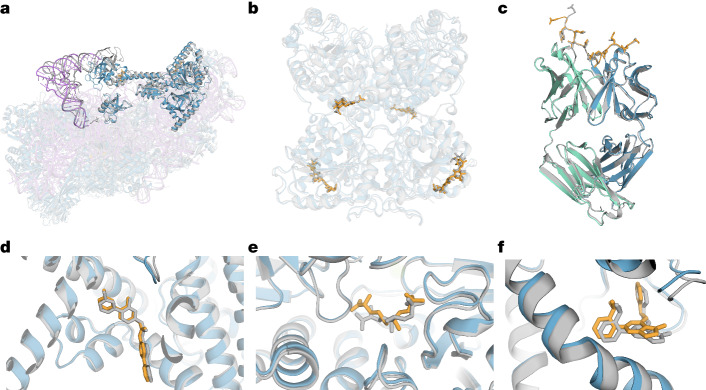
Examples of predicted complexes. Selected structure predictions from AF3. Predicted protein chains are shown in blue (predicted antibody in green), predicted ligands and glycans in orange, predicted RNA in purple and the ground truth is shown in grey. **a**, Human 40S small ribosomal subunit (7,663 residues) including 18S ribosomal RNA and Met-tRNA_i_^Met^ (opaque purple) in a complex with translation initiation factors eIF1A and eIF5B (opaque blue; PDB 7TQL; full-complex LDDT, 87.7; GDT, 86.9). **b**, The glycosylated globular portion of an EXTL3 homodimer (PDB 7AU2; mean pocket-aligned r.m.s.d., 1.10 Å). **c**, Mesothelin C-terminal peptide bound to the monoclonal antibody 15B6 (PDB 7U8C; DockQ, 0.85). **d**, LGK974, a clinical-stage inhibitor, bound to PORCN in a complex with the WNT3A peptide (PDB 7URD; ligand r.m.s.d., 1.00 Å). **e**, (5S,6S)-O7-sulfo DADH bound to the AziU3/U2 complex with a novel fold (PDB 7WUX; ligand r.m.s.d., 1.92 Å). **f**, Analogue of NIH-12848 bound to an allosteric site of PI5P4Kγ (PDB 7QIE; ligand r.m.s.d., 0.37 Å).

AF3 can predict structures from input polymer sequences, residue modifications and ligand SMILES (simplified molecular-input line-entry system). In Fig. 3 we show a selection of examples highlighting the ability of the model to generalize to a number of biologically important and therapeutically relevant modalities. In selecting these examples, we considered novelty in terms of the similarity of individual chains and interfaces to the training set (additional information is provided in Supplementary Methods 8.1).

**Extended Data Fig. 3:**
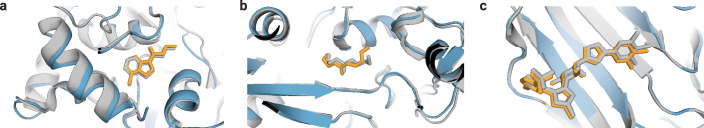
AlphaFold 3 predictions of PoseBusters examples for which Vina and Gold were inaccurate. Predicted protein chains are shown in blue, predicted ligands in orange, and ground truth in grey. **a**, Human Notum bound to inhibitor ARUK3004556 (PDB ID 8BTI, ligand RMSD: 0.65 Å). **b**, Pseudomonas sp. PDC86 Aapf bound to HEHEAA (PDB ID 7KZ9, ligand RMSD: 1.3 Å). **c**, Human Galectin-3 carbohydrate-recognition domain in complex with compound 22 (PDB ID 7XFA, ligand RMSD: 0.44 Å).

**Extended Data Fig. 4:**
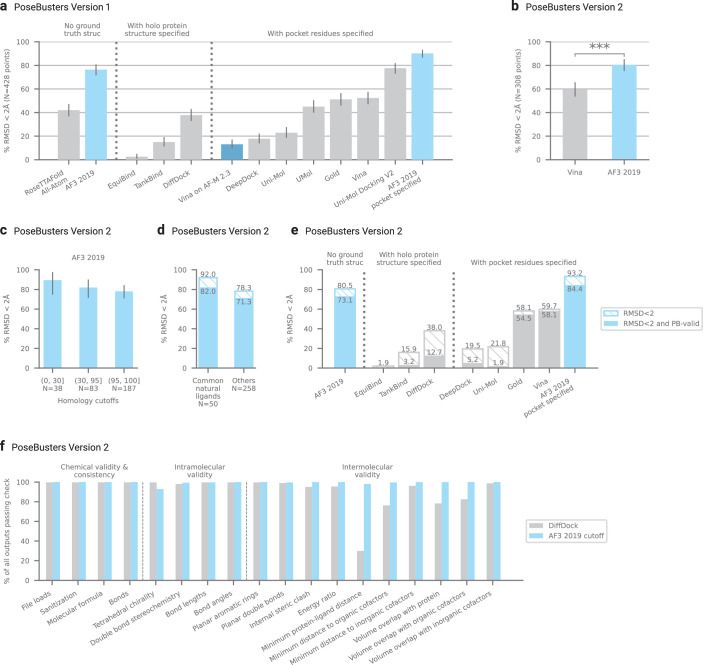
PoseBusters analysis. **a**, Comparison of AlphaFold 3 and baseline method protein-ligand binding success on the PoseBusters Version 1 benchmark set (V1, August 2023 release). Methods classified by the extent of ground truth information used to make predictions. Note all methods that use pocket residue information except for UMol and AF3 also use ground truth holo protein structures. **b**, PoseBusters Version 2 (V2, November 2023 release) comparison between the leading docking method Vina and AF3 2019 (two-sided Fisher exact test, N = 308 targets, p = 2.3 * 10^−8^). **c**, PoseBusters V2 results of AF3 2019 on targets with low, moderate, and high protein sequence homology (integer ranges indicate maximum sequence identity with proteins in the training set). **d**, PoseBusters V2 results of AF3 2019 with ligands split by those characterized as “common natural” ligands and others. “Common natural” ligands are defined as those which occur greater than 100 times in the PDB and which are not non-natural (by visual inspection). A full list may be found in Supplementary Table 15. Dark bar indicates RMSD < 2 Å and passing PoseBusters validity checks (PB-valid). **e**, PoseBusters V2 structural accuracy and validity. Dark bar indicates RMSD < 2 Å and passing PoseBusters validity checks (PB-valid). Light hashed bar indicates RMSD < 2 Å but not PB valid. **f**, PoseBusters V2 detailed validity check comparison. Error bars indicate exact binomial distribution 95% confidence intervals. N = 427 targets for RoseTTAFold All-Atom and 428 targets for all others in Version 1; 308 targets in Version 2.

We evaluated the performance of the system on recent interface-specific benchmarks for each complex type (Fig. 1c and Extended Data Table 1). Performance on protein–ligand interfaces was evaluated on the PoseBusters benchmark set, which is composed of 428 protein–ligand structures released to the PDB in 2021 or later. As our standard training cut-off date is in 2021, we trained a separate AF3 model with an earlier training-set cutoff (Methods). Accuracy on the PoseBusters set is reported as the percentage of protein–ligand pairs with pocket-aligned ligand root mean squared deviation (r.m.s.d.) of less than 2 Å. The baseline models come in two categories: those that use only protein sequence and ligand SMILES as an input and those that additionally leak information from the solved protein–ligand test structure. Traditional docking methods use the latter privileged information, even though that information would not be available in real-world use cases. Even so, AF3 greatly outperforms classical docking tools such as Vina^37,38^ even while not using any structural inputs (Fisher’s exact test, *P* = 2.27 × 10^−13^) and greatly outperforms all other true blind docking like RoseTTAFold All-Atom (*P* = 4.45 × 10^−25^). Extended Data Fig. 3 shows three examples in which AF3 achieves accurate predictions but docking tools Vina and Gold do not^37^. PoseBusters analysis was performed using a training cut-off of 30 September 2019 for AF3 to ensure that the model was not trained on any PoseBusters structures. To compare with the RoseTTAFold All-Atom results, we used PoseBusters version 1. Version 2 (crystal contacts removed from the benchmark set) results including quality metrics are shown in Extended Data Fig. 4b–f and Extended Data Table 1. We use multiple seeds to ensure correct chirality and avoid slight protein–ligand clashing (as opposed to a method like diffusion guidance to enforce) but we are typically able to produce high-quality stereochemistry. Separately, we also train a version of AF3 that receives the ‘pocket information’ as used in some recent deep-learning work^24,26^ (the results are shown in Extended Data Fig. 4a).

AF3 predicts protein–nucleic complexes and RNA structures with higher accuracy than RoseTTAFold2NA^15^ (Fig. 1c (second plot)). As RoseTTAFold2NA is validated only on structures below 1,000 residues, we use only structures below 1,000 residues from our recent PDB evaluation set for this comparison (Methods). AF3 is able to predict protein–nucleic structures with thousands of residues, an example of which is shown in Fig. 3a. Note that we do not compare directly to RoseTTAFold All-Atom, but benchmarks indicate that RoseTTAFold All-Atom is slightly less accurate than RoseTTAFold2NA for nucleic acid predictions^29^.

**Extended Data Fig. 5:**
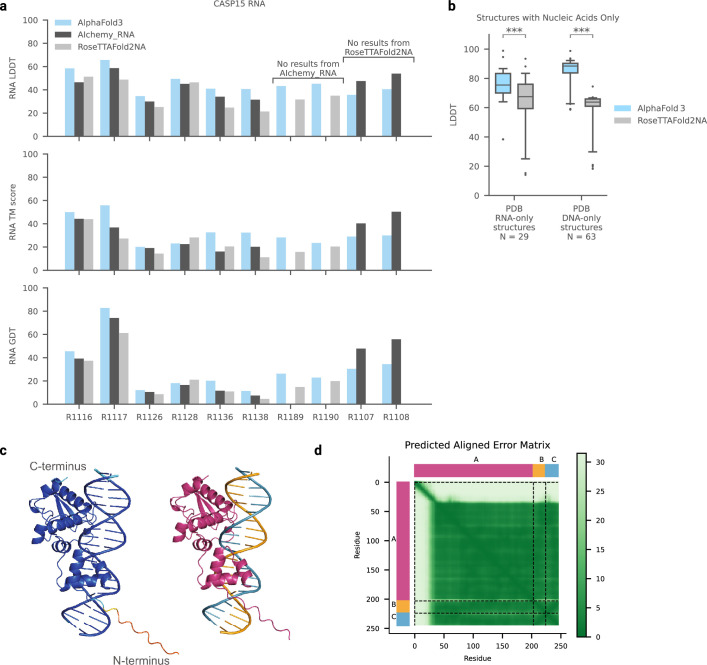
Nucleic acid prediction accuracy and confidences. **a**, CASP15 RNA prediction accuracy from AIChemy_RNA (the top AI-based submission), RoseTTAFold2NA (the AI-based method capable of predicting proteinRNA complexes), and AlphaFold 3. Ten of the 13 targets are available in the PDB or via the CASP15 website for evaluation. Predictions are downloaded from the CASP website for external models. **b**, Accuracy on structures containing low homology RNA-only or DNA-only complexes from the recent PDB evaluation set. Comparison between AlphaFold 3 and RoseTTAFold2NA (RF2NA) (RNA: N = 29 structures, paired Wilcoxon signed-rank test, p = 1.6 * 10^−7^; DNA: N = 63 structures, paired two-sided Wilcoxon signed-rank test, p = 5.2 * 10^−12^). Note RF2NA was only trained and evaluated on duplexes (chains forming at least 10 hydrogen bonds), but some DNA structures in this set may not be duplexes. Box, centerline, and whiskers boundaries are at (25%, 75%) intervals, median, and (5%, 95%) intervals. **c** Predicted structure of a mycobacteriophage immunity repressor protein bound to double stranded DNA (PDB ID 7R6R), coloured by pLDDT (left; orange: 0–50, yellow: 50–70, cyan 70–90, and blue 90–100) and chain id (right). Note the disordered N-terminus not entirely shown. **d**, Predicted aligned error (PAE) per token-pair for the prediction in c with rows and columns labelled by chain id and green gradient indicating PAE.

We also evaluated AF3 performance on the ten publicly available Critical Assessment of Structure Prediction 15 (CASP15) RNA targets: we achieve a higher average performance than RoseTTAFold2NA and AIchemy_RNA^27^ (the best AI-based submission in CASP15^18,31^) on the respective common subsets of our and their predictions (detailed results are shown in Extended Data Fig. 5a). We did not reach the performance of the best human-expert-aided CASP15 submission AIchemy_RNA2^39^ (Fig. 1c (centre left)). Owing to limited dataset sizes, we do not report significance test statistics here. Further analysis of the accuracy of predicting nucleic acids alone (without proteins) is shown in Extended Data Fig. 5b.

**Extended Data Fig. 6:**
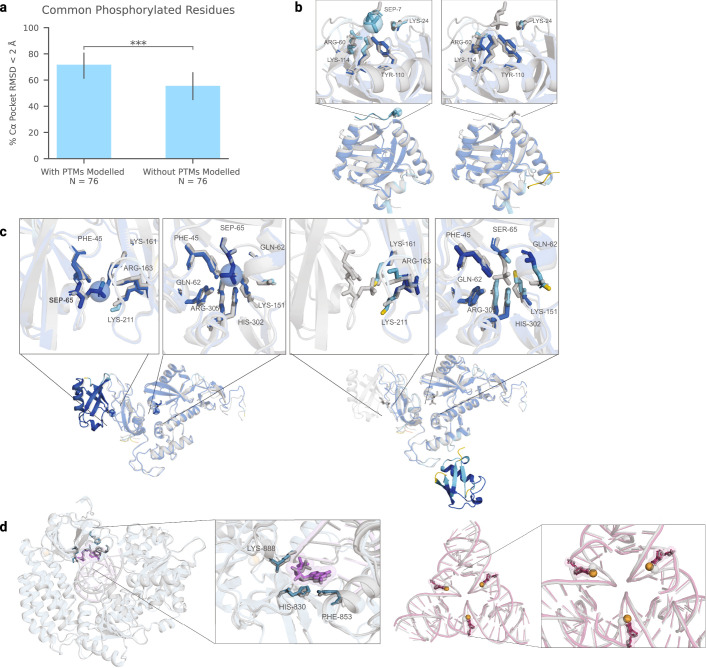
Analysis and examples for modified proteins and nucleic acids. **a**, Accuracy on structures. containing common phosphorylation residues (SEP, TPO, PTR, NEP, HIP) from the recent PDB evaluation set. Comparison between AlphaFold 3 with phosphorylation modelled, and AlphaFold 3 without modelling phosphorylation (N = 76 clusters, paired two-sided Wilcoxon signed-rank test, p = 1.6 * 10^−4^). Note, to predict a structure without modelling phosphorylation, we predict the parent (standard) residue in place of the modification. AlphaFold 3 generally achieves better backbone accuracy when modelling phosphorylation. Error bars indicate exact binomial distribution 95% confidence intervals. **b**, SPOC domain of human SHARP in complex with phosphorylated RNA polymerase II C-terminal domain (PDB ID 7Z1K), predictions coloured by pLDDT (orange: 0–50, yellow: 50–70, cyan 70–90, and blue 90–100). Left: Phosphorylation modelled (mean pocket-aligned RMSD_Cα_ 2.104 Å). Right: Without modelling phosphorylation (mean pocketaligned RMSD_Cα_ 10.261 Å). When excluding phosphorylation, AlphaFold 3 provides lower pLDDT confidence on the phosphopeptide. **c**, Structure of parkin bound to two phospho-ubiquitin molecules (PDB ID 7US1), predictions similarly coloured by pLDDT. Left: Phosphorylation modelled (mean pocket-aligned RMSD_Cα_ 0.424 Å). Right: Without modelling phosphorylation (mean pocket-aligned RMSD_Cα_ 9.706 Å). When excluding phosphorylation, AlphaFold 3 provides lower pLDDT confidence on the interface residues of the incorrectly predicted ubiquitin. **d**, Example structures with modified nucleic acids. Left: Guanosine monophosphate in RNA (PDB ID 7TNZ, mean pocket-aligned modified residue RMSD 0.840 Å). Right: Methylated DNA cytosines (PDB ID 7SDW, mean pocket-aligned modified residue RMSD 0.502 Å). Welabel residues of the predicted structure for reference. Ground truth structure in grey; predicted protein in blue, predicted RNA in purple, predicted DNA in magenta, predicted ions in orange, with predicted modifications highlighted via spheres.

Covalent modifications (bonded ligands, glycosylation, and modified protein residues and nucleic acid bases) are also accurately predicted by AF3 (Fig. 1c (centre right)). Modifications include those to any polymer residue (protein, RNA or DNA). We report accuracy as the percentage of successful predictions (pocket r.m.s.d. < 2 Å). We apply quality filters to the bonded ligands and glycosylation dataset (as does PoseBusters): we include only ligands with high-quality experimental data (ranking_model_fit > 0.5, according to the RCSB structure validation report, that is, X-ray structures with a model quality above the median). As with the PoseBusters set, the bonded ligands and glycosylation datasets are not filtered by homology to the training dataset. Filtering on the basis of the bound polymer chain homology (using polymer template similarity < 40) yielded only five clusters for bonded ligands and seven clusters for glycosylation. We exclude multi-residue glycans here because the RCSB validation report does not provide a ranking_model_fit value for them. The percentage of successful predictions (pocket r.m.s.d. < 2 Å) for multi-residue glycans on all-quality experimental data is 42.1% (*n* = 131 clusters), which is slightly lower than the success rate for single-residue glycans on all-quality experimental data of 46.1% (*n* = 167). The modified residues dataset is filtered similarly to our other polymer test sets: it contains only modified residues in polymer chains with low homology to the training set (Methods). See Extended Data Table 1 for detailed results, and Extended Data Fig. 6 for examples of predicted protein, DNA and RNA structures with covalent modifications, including analysis of the impact of phosphorylation on predictions.

**Extended Data Fig. 7:**
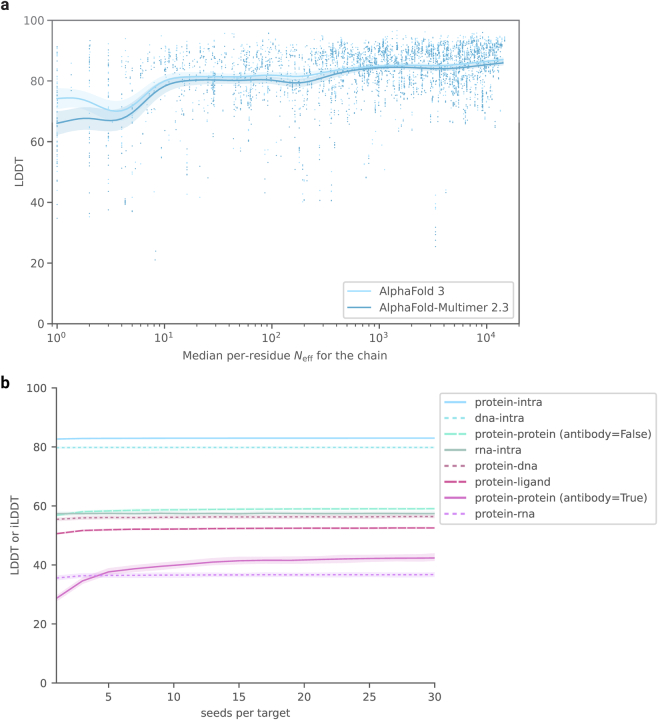
Model accuracy with MSA size and number of seeds. **a**, Effect of MSA depth on protein prediction accuracy. Accuracy is given as single chain LDDT score and MSA depth is computed by counting the number of non-gap residues for each position in the MSA using the *N*_*eff*_ weighting scheme and taking the median across residues (see Methods for details on *N*_*eff*_). MSA used for AF-M 2.3 differs slightly from AF3; the data uses the AF3 MSA depth for both to make the comparison clearer. The analysis uses every protein chain in the low homology Recent PDB set, restricted to chains in complexes with fewer than 20 protein chains and fewer than 2,560 tokens (see Methods for details on Recent PDB set and comparisons to AF-M 2.3). The curves are obtained through Gaussian kernel average smoothing (window size is 0.2 units in log10(*N*_*eff*_)); the shaded area is the 95% confidence interval estimated using bootstrap of 10,000 samples. **b**, Increase in ranked accuracy with number of seeds for different molecule types. Predictions are ranked by confidence, and only the most confident per interface is scored. Evaluated on the low homology recent PDB set, filtered to less than 1,536 tokens. Number of clusters evaluated: dna-intra = 386, protein-intra = 875, rnaintra = 78, protein-dna = 307, protein-rna = 102, protein-protein (antibody = False) = 697, protein-protein (antibody = True) = 58. Confidence intervals are 95% bootstraps over 1,000 samples.

While expanding in modelling abilities, AF3 has also improved in protein complex accuracy relative to AlphaFold-Multimer (v.2.3)^7,8^. Generally, protein–protein prediction success (DockQ > 0.23)^40^ has increased (paired Wilcoxon signed-rank test, *P* = 1.8 × 10^−18^), with antibody–protein interaction prediction in particular showing a marked improvement (Fig. 1c (right); paired Wilcoxon signed-rank test, *P* = 6.5 × 10^−5^, predictions top-ranked from 1,000 rather than the typical 5 seeds; further details are provided in Fig. 5a). Protein monomer LDDT improvement is also significant (paired Wilcoxon signed-rank test, *P* = 1.7 × 10^−34^). AF3 has a very similar dependence on MSA depth to AlphaFold-Multimer v.2.3; proteins with shallow MSAs are predicted with lower accuracy (a comparison of the dependence of single-chain LDDT on MSA depth is shown in Extended Data Fig. 7a).

## Predicted confidences track accuracy

**Extended Data Fig. 8:**
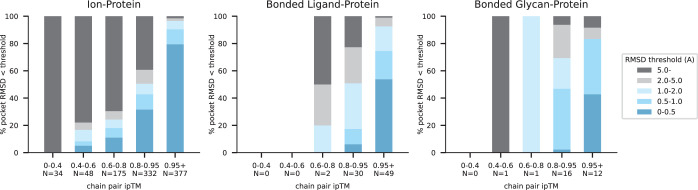
Relationship between confidence and accuracy for protein interactions with ions, bonded ligands and bonded glycans. Accuracy is given as the percentage of interface clusters under various pocket-aligned RMSD thresholds, as a function of the chain pair ipTM of the interface. The ions group includes both metals and nonmetals. N values report the number of clusters in each band. For a similar analysis on general ligand-protein interfaces, see Fig. 4 of main text.

As with AF2, AF3 confidence measures are well calibrated with accuracy. Our confidence analysis is performed on the recent PDB evaluation set, with no homology filtering and including peptides. The ligands category is filtered to high-quality experimental structures as described above, and considers standard non-bonded ligands only. See Extended Data Fig. 8 for a similar assessment on bonded ligand and other interfaces. All statistics are cluster-weighted (Methods) and consider the top-ranked prediction only (ranking details are provided in Supplementary Methods 5.9.3).

**Fig. 4:**
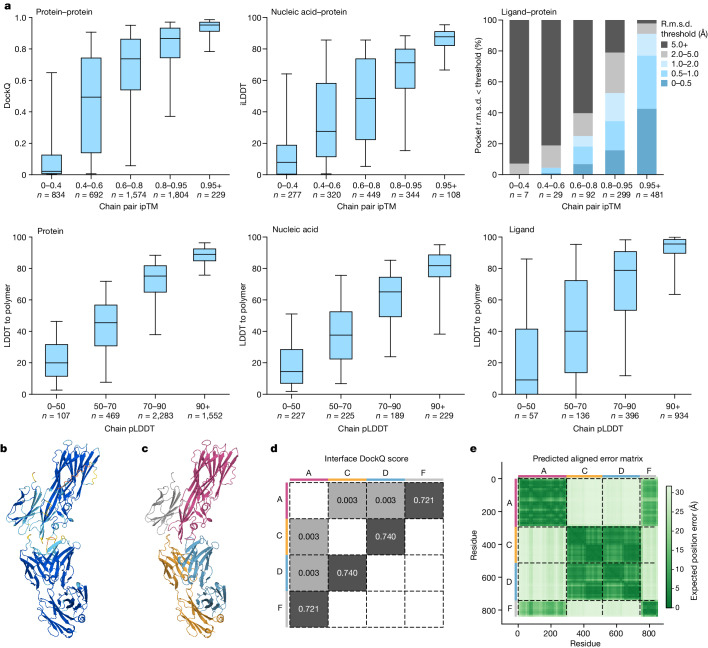
AF3 confidences track accuracy. **a**, The accuracy of protein-containing interfaces as a function of chain pair ipTM (top). Bottom, the LDDT-to-polymer accuracy was evaluated for various chain types as a function of chain-averaged pLDDT. The box plots show the 25–75% confidence intervals (box limits), the median (centre line) and the 5–95% confidence intervals (whiskers). *n* values report the number of clusters in each band. **b**, The predicted structure of PDB 7T82 coloured by pLDDT (orange, 0–50; yellow, 50–70; cyan, 70–90; and blue, 90–100). **c**, The same prediction coloured by chain. **d**, DockQ scores for protein–protein interfaces. **e**, PAE matrix of same prediction (darker is more confident), with chain colouring of **c** on the side bars. The dashed black lines indicate the chain boundaries.

In Fig. 4a (top row), we plot the chain pair interface-predicted TM (ipTM) score^41^ (Supplementary Methods 5.9.1) against interface accuracy measures: protein–protein DockQ, protein–nucleic interface LDDT (iLDDT) and protein–ligand success, with success defined as the percentage of examples under thresholded pocket-aligned r.m.s.d. values. In Fig. 4a (bottom row), we plot the average pLDDT per protein, nucleotide or ligand entity against our bespoke LDDT_to_polymer metric (metrics details are provided in the Methods), which is closely related to the training target of the pLDDT predictor.

In Fig. 4b–e, we highlight a single example prediction of 7T82, in which per-atom pLDDT colouring identifies unconfident chain tails, somewhat confident interfaces and otherwise confident secondary structure. In Fig. 4c, the same prediction is coloured by chain, along with DockQ interface scores in Fig. 4d and per-chain colouring displayed on the axes for reference. We see from Fig. 4e that PAE confidence is high for pink–grey and blue–orange residue pairs for which DockQ > 0.7, and least confident about pink–orange and pink–blue residue pairs that have DockQ ≈ 0. A similar PAE analysis of an example with protein and nucleic acid chains is shown in Extended Data Fig. 5c,d.

## Model limitations

We note model limitations of AF3 with respect to stereochemistry, hallucinations, dynamics and accuracy for certain targets.

**Fig. 5:**
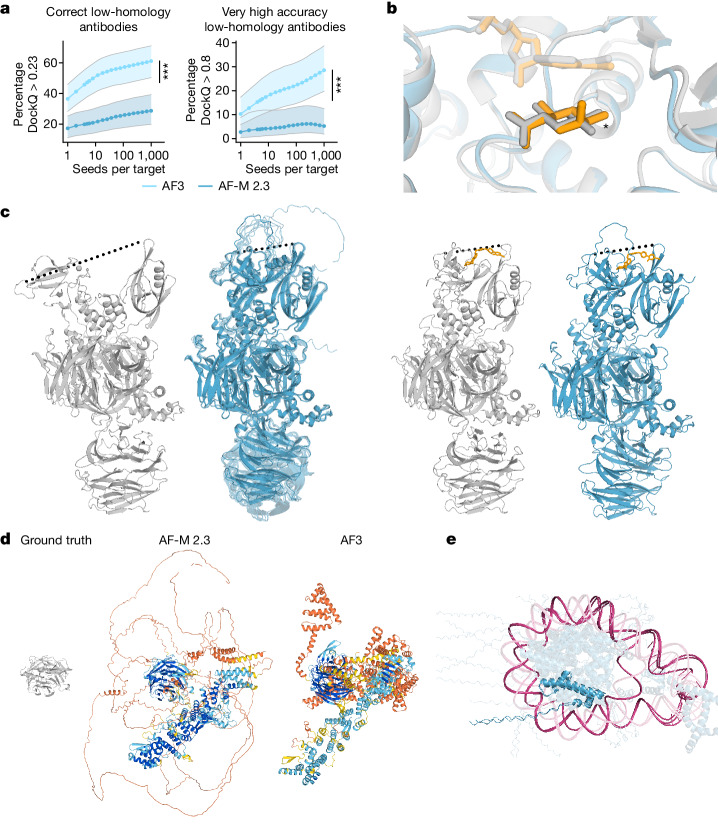
Model limitations. **a**, Antibody prediction quality increases with the number of model seeds. The quality of top-ranked, low-homology antibody–antigen interface predictions as a function of the number of seeds. Each datapoint shows the mean over 1,000 random samples (with replacement) of seeds to rank over, out of 1,200 seeds. Confidence intervals are 95% bootstraps over 10,000 resamples of cluster scores at each datapoint. Samples per interface are ranked by protein–protein ipTM. Significance tests were performed using by two-sided Wilcoxon signed-rank tests. *n* = 65 clusters. Exact *P* values were as follows: 2.0 × 10^−5^ (percentage correct) and *P* = 0.009 (percentage very high accuracy). **b**, Prediction (coloured) and ground-truth (grey) structures of *Thermotoga maritima* α-glucuronidase and beta-d-glucuronic acid—a target from the PoseBusters set (PDB: 7CTM). AF3 predicts alpha-d-glucuronic acid; the differing chiral centre is indicated by an asterisk. The prediction shown is top-ranked by ligand–protein ipTM and with a chirality and clash penalty. **c**, Conformation coverage is limited. Ground-truth structures (grey) of cereblon in open (apo, PDB: 8CVP; left) and closed (holo mezigdomide-bound, PDB: 8D7U; right) conformations. Predictions (blue) of both apo (with 10 overlaid samples) and holo structures are in the closed conformation. The dashed lines indicate the distance between the N-terminal Lon protease-like and C-terminal thalidomide-binding domain. **d**, A nuclear pore complex with 1,854 unresolved residues (PDB: 7F60). The ground truth (left) and predictions from AlphaFold-Multimer v.2.3 (middle) and AF3 (right) are shown. **e**, Prediction of a trinucleosome with overlapping DNA (pink) and protein (blue) chains (PDB: 7PEU); highlighted are overlapping protein chains B and J and self-overlapping DNA chain AA. Unless otherwise stated, predictions are top-ranked by our global complex ranking metric with chiral mismatch and steric clash penalties (Supplementary Methods 5.9.1).

On stereochemistry, we note two main classes of violations. The first is that the model outputs do not always respect chirality (Fig. 5b), despite the model receiving reference structures with correct chirality as input features. To address this in the PoseBusters benchmark, we included a penalty for chirality violation in our ranking formula for model predictions. Despite this, we still observe a chirality violation rate of 4.4% in the benchmark. The second class of stereochemical violations is a tendency of the model to occasionally produce overlapping (clashing) atoms in the predictions. This sometimes manifests as extreme violations in homomers in which entire chains have been observed to overlap (Fig. 5e). Penalizing clashes during ranking (Supplementary Methods 5.9.3) reduces the occurrence of this failure mode but does not eliminate them. Almost all remaining clashes occur for protein–nucleic complexes with both greater than 100 nucleotides and greater than 2,000 residues in total.

We note that the switch from the non-generative AF2 model to the diffusion-based AF3 model introduces the challenge of spurious structural order (hallucinations) in disordered regions (Fig. 5d and Extended Data Fig. 1). Although hallucinated regions are typically marked as very low confidence, they can lack the distinctive ribbon-like appearance that AF2 produces in disordered regions. To encourage ribbon-like predictions in AF3, we use distillation training from AF2 predictions, and we add a ranking term to encourage results with more solvent accessible surface area^36^.

A key limitation of protein structure prediction models is that they typically predict static structures as seen in the PDB, not the dynamical behaviour of biomolecular systems in solution. This limitation persists for AF3, in which multiple random seeds for either the diffusion head or the overall network do not produce an approximation of the solution ensemble.

In some cases, the modelled conformational state may not be correct or comprehensive given the specified ligands and other inputs. For example, E3 ubiquitin ligases natively adopt an open conformation in an apo state and have been observed only in a closed state when bound to ligands, but AF3 exclusively predicts the closed state for both holo and apo systems^42^ (Fig. 5c). Many methods have been developed, particularly around MSA resampling, that assist in generating diversity from previous AlphaFold models^43–45^ and may also assist in multistate prediction with AF3.

Despite the large advance in modelling accuracy in AF3, there are still many targets for which accurate modelling can be challenging. To obtain the highest accuracy, it may be necessary to generate a large number of predictions and rank them, which incurs an extra computational cost. A class of targets in which we observe this effect strongly is antibody–antigen complexes, similar to other recent work^46^. Figure 5a shows that, for AF3, top-ranked predictions keep improving with more model seeds, even at as many as 1,000 (Wilcoxon signed-rank test between 5 and 1,000 seeds, *P* = 2.0 × 10^−5^ for percentage correct and *P* = 0.009 for percentage very high accuracy; ranking by protein–protein interface ipTM). This large improvement with many seeds is not observed in general for other classes of molecules (Extended Data Fig. 7b). Using only one diffusion sample per model seed for the AF3 predictions rather than five (not illustrated) does not change the results significantly, indicating that running more model seeds is necessary for antibody score improvements, rather than just more diffusion samples.

## Discussion

The core challenge of molecular biology is to understand and ultimately regulate the complex atomic interactions of biological systems. The AF3 model takes a large step in this direction, demonstrating that it is possible to accurately predict the structure of a wide range of biomolecular systems in a unified framework. Although there are still substantial challenges to achieve highly accurate predictions across all interaction types, we demonstrate that it is possible to build a deep-learning system that shows strong coverage and generalization for all of these interactions. We also demonstrate that the lack of cross-entity evolutionary information is not a substantial blocker to progress in predicting these interactions and, moreover, substantial improvement in antibody results suggests AlphaFold-derived methods are able to model the chemistry and physics of classes of molecular interactions without dependence on MSAs. Finally, the large improvement in protein–ligand structure prediction shows that it is possible to handle the wide diversity of chemical space within a general deep-learning framework and without resorting to an artificial separation between protein structure prediction and ligand docking.

The development of bottom-up modelling of cellular components is a key step in unravelling the complexity of molecular regulation within the cell, and the performance of AF3 shows that developing the right deep-learning frameworks can massively reduce the amount of data required to obtain biologically relevant performance on these tasks and amplify the impact of the data already collected. We expect that structural modelling will continue to improve not only due to advances in deep learning but also because continuing methodological advances in experimental structure determination, such as the substantial improvements in cryo-electron microscopy and tomography, will provide a wealth of new training data to further the improve the generalization ability of such models. The parallel developments of experimental and computational methods promise to propel us further into an era of structurally informed biological understanding and therapeutic development.

## Methods

### Full algorithm details

Extensive explanations of the components are available in Supplementary Methods 2–5. Moreover, pseudocode is available in Supplementary Algorithms 1–31, network diagrams in Figs. 1d and  2a–c and Supplementary Fig. 2, input features in Supplementary Table 5 and additional hyperparameters for training in Supplementary Tables 3, 4 and 7.

### Training regime

No structural data used during training were released after 30 September 2021 and, for the model used in PoseBusters evaluations, we filtered out PDB^32^ structures released after 30 September 2021. One optimizer step uses a mini batch of 256 input data samples and during initial training 256 × 48 = 12,288 diffusion samples. For fine-tuning, the number of diffusion samples is reduced to 256 × 32 = 8,192. The model is trained in three stages—the initial training with a crop size of 384 tokens and two sequential fine tuning stages with crop sizes of 640 and 768 tokens. Further details are provided in Supplementary Methods 5.2.

### Inference regime

No inference time templates or reference ligand position features were released after 30 September 2021, and in the case of PoseBusters evaluation, an earlier cut-off date of 30 September 2019 was used. The model can be run with different random seeds to generate alternative results, with a batch of diffusion samples per seed. Unless otherwise stated, all results are generated by selecting the top confidence sample from running 5 seeds of the same trained model, with 5 diffusion samples per model seed, for a total of 25 samples to choose from. Standard crystallization aids are excluded from predictions (Supplementary Table 8).

Results are shown for the top-ranked sample and sample ranking depends on whether trying to select the overall best output globally, or the best output for some chain, interface or modified residue. Global ranking uses a mix of pTM and ipTM along with terms to reduce cases with large numbers of clashes and increase rates of disorder; individual chain ranking uses a chain specific pTM measure; interface ranking uses a bespoke ipTM measure for the relevant chain pair; and modified residue ranking uses average pLDDT over the residue of interest (Supplementary Methods 5.9.3).

### Metrics

Evaluation compares a predicted structure to the corresponding ground-truth structure. If the complex contains multiple identical entities, assignment of the predicted units to the ground-truth units is found by maximizing LDDT. Assignment in local symmetry groups of atoms in ligands is solved by exhaustive search over the first 1,000 per-residue symmetries as given by RDKit.

**Extended Data Fig. 9:**
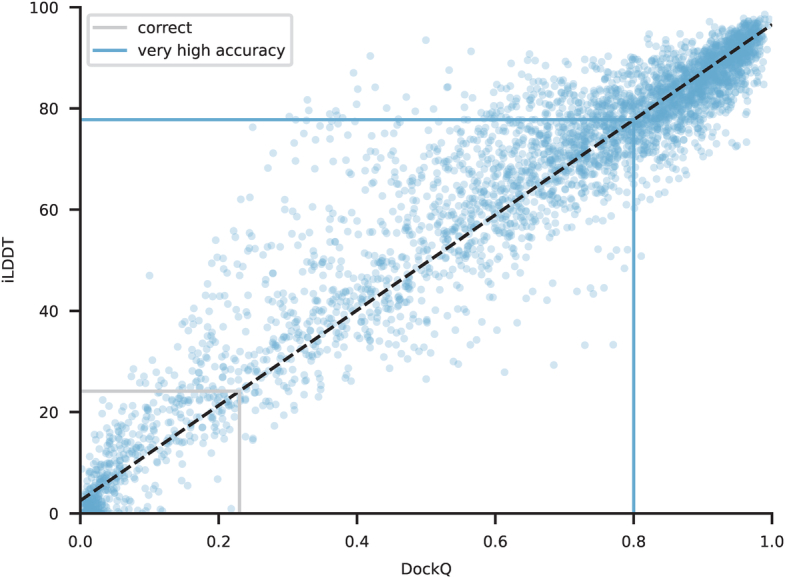
Correlation of DockQ and iLDDT for protein-protein interfaces. One data point per cluster, 4,182 clusters shown. Line of best fit with a Huber regressor with epsilon 1. DockQ categories correct (>0.23), and very high accuracy (>0.8) correspond to iLDDTs of 23.6 and 77.6 respectively.

We measure the quality of the predictions with DockQ, LDDT or pocket-aligned r.m.s.d. For nucleic–protein interfaces, we measure interface accuracy through iLDDT, which is calculated from distances between atoms across different chains in the interface. DockQ and iLDDT are highly correlated (Extended Data Fig. 9), so the standard cut-offs for DockQ can be translated to equivalent iLDDT cut-offs. Nucleic acid LDDTs (intrachains and interface) were calculated with an inclusion radius of 30 Å compared with the usual 15 Å used for proteins, owing to their larger scale. For confidence calibration assessment, we use a bespoke LDDT (LDDT_to_polymer) metric that considers differences from each atom of a given entity to any C^α^ or C1′ polymer atom within its inclusion radius. This is closely related to how the confidence prediction is trained (Supplementary Methods 4.3.1).

Pocket-aligned r.m.s.d. is computed as follows: the pocket is defined as all heavy atoms within 10 Å of any heavy atom of the ligand, restricted to the primary polymer chain for the ligand or modified residue being scored, and further restricted to only backbone atoms for proteins. The primary polymer chain is defined variously: for PoseBusters, it is the protein chain with the most atoms within 10 Å of the ligand; for bonded ligand scores, it is the bonded polymer chain; and for modified residues, it is the chain in which the residue is contained (minus that residue). The pocket is used to align the predicted structure to the ground-truth structure with least-squares rigid alignment and then the r.m.s.d. is computed on all heavy atoms of the ligand.

### Recent PDB evaluation set

General model evaluation was performed on our recent PDB set consisting of 8,856 PDB complexes released between 1 May 2022 and 12 January 2023. The set contains almost all PDB complexes released during that period that are less than 5,120 model tokens in size (Supplementary Methods 6.1). Single chains and interfaces within each structure were scored separately rather than only looking at full complex scores, and clustering was then applied to chains and interfaces so that scores could be aggregated first within clusters and then across clusters for mean scores, or using a weighting of inverse cluster size for distributional statistics (Supplementary Methods 6.2 and 6.4).

Evaluation on ligands excludes standard crystallization aids (Supplementary Table 8), our ligand exclusion list (Supplementary Table 9) and glycans (Supplementary Table 10). Bonded and non-bonded ligands are evaluated separately. Ions are only included when specifically mentioned (Supplementary Table 11).

The recent PDB set is filtered to a low homology subset (Supplementary Methods 6.1) for some results where stated. Homology is defined as sequence identity to sequences in the training set and is measured by template search (Supplementary Methods 2.4). Individual polymer chains in evaluation complexes are filtered out if the maximum sequence identity to chains in the training set is greater than 40%, where sequence identity is the percentage of residues in the evaluation set chain that are identical to the training set chain. Individual peptide chains (protein chains with less than 16 residues) are always filtered out. For polymer–polymer interfaces, if both polymers have greater than 40% sequence identity to two chains in the same complex in the training set, then the interface is filtered out. For interfaces to a peptide, the interface is filtered out if the non-peptide entity has greater than 40% sequence identity to any chain in the training set.

To compare the quality of prediction of protein–protein interfaces and protein monomers against that of AlphaFold-Multimer (v.2.3)^8^, and to compare the dependence of single-protein-chain prediction quality on MSA depth, we restrict the low-homology recent PDB set to complexes with fewer than 20 protein chains and fewer than 2,560 tokens. We compare against unrelaxed AlphaFold-Multimer v.2.3 predictions.

To study antibody-antigen interface prediction, we filter the low homology recent PDB set to complexes that contain at least one protein–protein interface where one of the protein chains is in one of the two largest PDB chain clusters (these clusters are representative of antibodies). We further filter to complexes with at most 2,560 tokens and with no unknown amino acids in the PDB to allow extensive comparison against relaxed predictions of AlphaFold-Multimer v2.3. That leaves 71 antibody–antigen complexes, containing 166 antibody–antigen interfaces spanning 65 interface clusters.

MSA depth analysis (Extended Data Fig. 7a) was based on computing the normalized number of effective sequences (*N*_eff_) for each position of a query sequence. Per-residue *N*_eff_ values were obtained by counting the number of non-gap residues in the MSA for this position and weighting the sequences using the *N*_eff_ scheme^49^ with a threshold of 80% sequence identity measured on the region that is non-gap in either sequence.

### Nucleic acid prediction baseline

For benchmarking performance on nucleic acid structure prediction, we report baseline comparisons to an existing machine learning system for protein–nucleic acid and RNA tertiary structure prediction, RoseTTAFold2NA^18^. We run the open source RF2NA^50^ with the same MSAs as those that were used for AF3 predictions. For comparison between AF3 and RF2NA, a subset of our recent PDB set was chosen to meet the RF2NA criteria (<1,000 total residues and nucleotides). As RF2NA was not trained to predict systems with DNA and RNA, analysis is limited to targets with only one nucleic acid type. No system was publicly available at time of writing for baseline comparisons on data with arbitrary combinations of biomolecular types in PDB.

As an additional baseline for RNA tertiary structure prediction, we evaluate AF3 performance on CASP15 RNA targets that were publicly available as of 1 December 2023 (R1116/8S95, R1117/8FZA, R1126 (downloaded from the CASP15 website https://predictioncenter.org/casp15/TARGETS_PDB/R1126.pdb), R1128/8BTZ, R1136/7ZJ4, R1138/[7PTK/7PTL], R1189/7YR7 and R1190/7YR6). We compare the top-1 ranked predictions and, where multiple ground-truth structures exist (R1136), the prediction is scored against the closest state. We display comparisons to RF2NA as a representative machine learning system; AIchemy_RNA2 as the top performing entrant with human intervention; and AIchemy_RNA as the top performing machine learning system. All entrants’ predictions were downloaded from the CASP website and scored internally.

### PoseBusters

While other analyses used an AlphaFold model trained on PDB data released before a cut-off of 30 September 2021, our PoseBusters analysis was conducted on a model (with identical architecture and similar training schedule) differing only in the use of an earlier 30 September 2019 cut-off. This analysis therefore did not include training data, inference time templates or ‘ref_pos’ features released after this date.

Inference was performed on the asymmetric unit from specified PDBs, with the following minor modifications. In several PDB files, chains clashing with the ligand of interest were removed (7O1T, 7PUV, 7SCW, 7WJB, 7ZXV, 8AIE). Another PDB entry (8F4J) was too large to inference the entire system (over 5,120 tokens), so we included only protein chains within 20 Å of the ligand of interest. Five model seeds, each with five diffusion samples, were produced per target, resulting in 25 predictions, which were ranked by quality and predicted accuracy: the ranking score was calculated from an ipTM aggregate (Supplementary Methods 5.9.3 (point 3)), then further divided by 100 if the ligand had chirality errors or had clashes with the protein.

For pocket-aligned r.m.s.d., first alignment between the predicted and ground-truth structures was conducted by aligning to the ground-truth pocket backbone atoms (CA, C or N atoms within 10 Å of the ligand of interest) from the primary protein chain (the chain with the greatest number of contacts within 10 Å of the ligand). The PoseBusters Python package v.0.2.7^51^ was used to score r.m.s.d. and violations from the pocket-aligned predictions.

While AlphaFold models are ‘blind’ to the protein pocket, docking is often performed with knowledge of the protein pocket residues. For example, Uni-Mol specifies the pocket as any residue within 6 Å of the heavy atoms in the ligand of interest^26^. To evaluate the ability of AF3 to dock ligands accurately when given pocket information, we fine-tuned a 30 September 2019 cut-off AF3 model with an additional token feature specifying pocket–ligand pairs (Supplementary Methods 2.8). Specifically, an additional token feature was introduced, set to true for a ligand entity of interest and any pocket residues with heavy atoms within 6 Å of the ligand entity. At training time, a single random ligand entity is chosen to use in this feature. Note that multiple ligand chains with the same entity (CCD code) may be selected. At inference time, the ligand entity was chosen based on the ligand of interest’s CCD code, so again multiple ligand chains were occasionally chosen. The results of this analysis are shown in Extended Data Fig. 4.

### Model performance analysis and visualization

Data analysis used Python v.3.11.7 (https://www.python.org/), NumPy v.1.26.3 (https://github.com/numpy/numpy), SciPy v.1.9.3 (https://www.scipy.org/), seaborn v.0.12.2 (https://github.com/mwaskom/seaborn), Matplotlib v.3.6.1 (https://github.com/matplotlib/matplotlib), pandas v.2.0.3 (https://github.com/pandas-dev/pandas), statsmodels v.0.12.2 (https://github.com/statsmodels/statsmodels), RDKit v.4.3.0 (https://github.com/rdkit/rdkit) and Colab (https://research.google.com/colaboratory). TM-align v.20190822 (https://zhanglab.dcmb.med.umich.edu/TM-align/) was used for computing TM-scores. Structure visualizations were created in Pymol v.2.55.5 (https://github.com/schrodinger/pymol-open-source).

### Reporting summary

Further information on research design is available in the Nature Portfolio Reporting Summary linked to this article.

## Online content

Any methods, additional references, Nature Portfolio reporting summaries, source data, extended data, supplementary information, acknowledgements, peer review information; details of author contributions and competing interests; and statements of data and code availability are available at 10.1038/s41586-024-07487-w.

## Supplementary information

Supplementary Information Supplementary Information 1 (notation), 2 (data pipeline), 3 (model architecture), 4 (auxiliary heads), 5 (training and inference), 6 (evaluation), 7 (differences to AlphaFold2 and AlphaFold-Multimer), 8 (Supplementary Results) and 9 (Appendix, including CCD Code and PDB ID tables).

Reporting Summary

Supplementary Data AF3 predictions for the Posebusters benchmark and outputs of the Posebusters checks for those predictions.

## Figures and Tables

**Extended Data Table 1:**
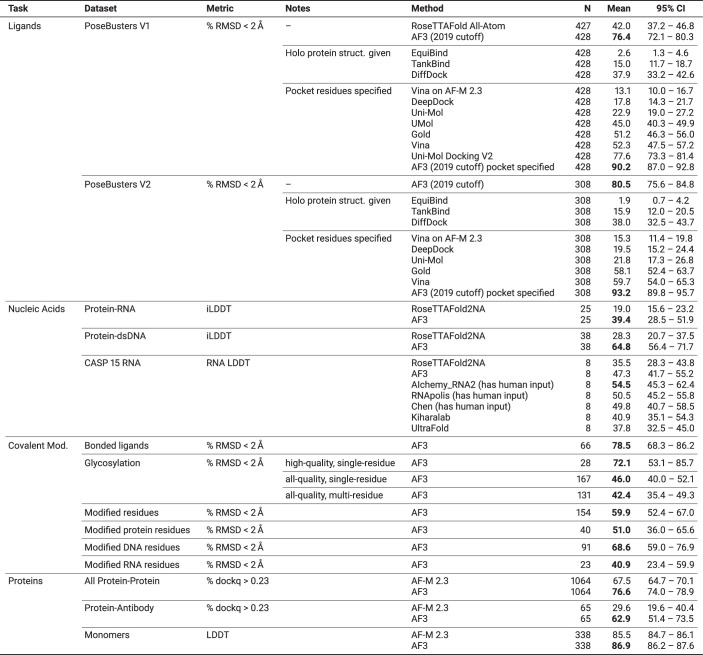
Prediction accuracy across biomolecular complexes

AlphaFold 3 Performance on PoseBusters V1 (August 2023 release), PoseBusters V2 (November 6th 2023 release), and our Recent PDB evaluation set. For ligands and nucleic acids N indicates number of structures; for covalent modifications and proteins N indicates number of clusters.

## Data Availability

All scientific datasets used to create training and evaluation inputs are freely available from public sources. Structures from the PDB were used for training and as templates (https://files.wwpdb.org/pub/pdb/data/assemblies/mmCIF/; sequence clusters are available at https://cdn.rcsb.org/resources/sequence/clusters/clusters-by-entity-40.txt; sequence data are available at https://files.wwpdb.org/pub/pdb/derived_data/). Training used a version of the PDB downloaded 12 January 2023, while template search used a version downloaded 28 September 2022. We also used the Chemical Components Dictionary downloaded on 19 October 2023 (https://www.wwpdb.org/data/ccd). We show experimental structures from the PDB under accession numbers 7PZB (ref. ^52^), 7PNM (ref. ^53^), 7TQL (ref. ^54^), 7AU2 (ref. ^55^), 7U8C (ref. ^56^), 7URD (ref. ^57^), 7WUX (ref. ^58^), 7QIE (ref. ^59^), 7T82 (ref. ^60^), 7CTM (ref. ^61^), 8CVP (ref. ^42^), 8D7U (ref. ^42^), 7F60 (ref. ^62^), 8BTI (ref. ^63^), 7KZ9 (ref. ^64^), 7XFA (ref. ^65^), 7PEU (ref. ^66^), 7SDW (ref. ^67^), 7TNZ (ref. ^68^), 7R6R (ref. ^69^), 7USR (ref. ^70^) and 7Z1K (ref. ^71^). We also used the following publicly available databases for training or evaluation. Detailed usage is described in Supplementary Methods 2.2 and 2.5.2. UniRef90 v.2020_01 (https://ftp.ebi.ac.uk/pub/databases/uniprot/previous_releases/release-2020_01/uniref/), UniRef90 v.2020_03 (https://ftp.ebi.ac.uk/pub/databases/uniprot/previous_releases/release-2020_03/uniref/), UniRef90 v.2022_05 (https://ftp.ebi.ac.uk/pub/databases/uniprot/previous_releases/release-2022_05/uniref/), Uniclust30 v.2018_08 (https://wwwuser.gwdg.de/~compbiol/uniclust/2018_08/), Uniclust30 v.2021_03 (https://wwwuser.gwdg.de/~compbiol/uniclust/2021_03/), MGnify clusters v.2018_12 (https://ftp.ebi.ac.uk/pub/databases/metagenomics/peptide_database/2018_12/), MGnify clusters v.2022_05 (https://ftp.ebi.ac.uk/pub/databases/metagenomics/peptide_database/2022_05/), BFD (https://bfd.mmseqs.com), RFam v.14.9 (https://ftp.ebi.ac.uk/pub/databases/Rfam/14.9/), RNAcentral v.21.0 (https://ftp.ebi.ac.uk/pub/databases/RNAcentral/releases/21.0/), Nucleotide Database (as of 23 February 2023) (https://ftp.ncbi.nlm.nih.gov/blast/db/FASTA/nt.gz), JASPAR 2022 (https://jaspar.elixir.no/downloads/; see https://jaspar.elixir.no/profile-versions for version information), SELEX protein sequences from the supplementary tables of ref. ^72^ and SELEX protein sequences from the supplementary tables of ref. ^73^.

## Code availability

AlphaFold 3 will be available as a non-commercial usage only server at https://www.alphafoldserver.com, with restrictions on allowed ligands and covalent modifications. Pseudocode describing the algorithms is available in the Supplementary Information. Code is not provided.
