# Addendum: Accurate structure prediction of biomolecular interactions with AlphaFold 3

**DOI:** 10.1038/s41586-024-08416-7

**Published:** 2024-11-27

**Authors:** Josh Abramson, Jonas Adler, Jack Dunger, Richard Evans, Tim Green, Alexander Pritzel, Olaf Ronneberger, Lindsay Willmore, Andrew J. Ballard, Joshua Bambrick, Sebastian W. Bodenstein, David A. Evans, Chia-Chun Hung, Michael O’Neill, David Reiman, Kathryn Tunyasuvunakool, Zachary Wu, Akvilė Žemgulytė, Eirini Arvaniti, Charles Beattie, Ottavia Bertolli, Alex Bridgland, Alexey Cherepanov, Miles Congreve, Alexander I. Cowen-Rivers, Andrew Cowie, Michael Figurnov, Fabian B. Fuchs, Hannah Gladman, Rishub Jain, Yousuf A. Khan, Caroline M. R. Low, Kuba Perlin, Anna Potapenko, Pascal Savy, Sukhdeep Singh, Adrian Stecula, Ashok Thillaisundaram, Catherine Tong, Sergei Yakneen, Ellen D. Zhong, Michal Zielinski, Augustin Žídek, Victor Bapst, Pushmeet Kohli, Max Jaderberg, Demis Hassabis, John M. Jumper

**Affiliations:** 1 Core Contributor, Google DeepMind, London, UK; 2 Core Contributor, Isomorphic Labs, London, UK; 3 Google DeepMind, London, UK; 4 Isomorphic Labs, London, UK; 5 Department of Molecular and Cellular Physiology, Stanford University, Stanford, CA USA; 6 Department of Computer Science, Princeton University, Princeton, NJ USA

**Keywords:** Protein structure predictions, Structural biology, Machine learning, Drug discovery

Addendum to: *Nature* 10.1038/s41586-024-07487-w Published online 8 May 2024

In our original article, we provided an extensive description in the Supplementary Information of pseudocode underlying the functioning of our AlphaFold3 model, which allows prediction of the structure of complexes including proteins, nucleic acids, small molecules, ions and modified residues. Accompanying release of the paper we provided free non-commercial access to the AlphaFold3 server (https://alphafoldserver.com/welcome). Google DeepMind has now released the underlying inference code, which can be accessed here: https://github.com/google-deepmind/alphafold3.

