# Supplementary material for: Accurate structure prediction of biomolecular interactions with AlphaFold 3
Source: Nature. 2024 May 8;630(8016):493–500. doi: 10.1038/s41586-024-07487-w (PMC11168924; doi:10.1038/s41586-024-07487-w)
Supplement: Supplementary file 1 — Supplementary Information 1 (notation), 2 (data pipeline), 3 (model architecture), 4 (auxiliary heads), 5 (training and inference), 6 (evaluation), 7 (differences to AlphaFold2 and AlphaFold-Multimer), 8 (Supplementary Results) and 9 (Appendix, including CCD Code and PDB ID tables). [file 41586_2024_7487_MOESM1_ESM.pdf]

nature portfolio

https://doi.org/10.1038/s41586-024-07487-w

Supplementary information

Accurate structure prediction of biomolecular interactions with AlphaFold 3

In the format provided by the
authors and unedited

Accurate structure prediction of biomolecular interactions with AlphaFold 3

Josh Abramson[*1], Jonas Adler[*1], Jack Dunger[*1], Richard Evans[*1], Tim Green[*1], Alexander Pritzel[*1], Olaf Ronneberger[*1], Lindsay Willmore[*1], Andrew J Ballard[1], Joshua Bambrick[2], Sebastian W Bodenstein[1], David A Evans[1], Chia-Chun Hung[2], Michael O'Neill[1], David Reiman[1], Kathryn Tunyasuvunakool[1], Zachary Wu[1], Akvilė Žemgulytė[1], Eirini Arvaniti[3], Charles Beattie[3], Ottavia Bertolli[3], Alex Bridgland[3], Alexey Cherepanov[4], Miles Congreve[4], Alexander I Cowen-Rivers[3], Andrew Cowie[3], Michael Figurnov[3], Fabian B Fuchs[3], Hannah Gladman[3], Rishub Jain[3], Yousuf A Khan[3], Caroline M R Low[4], Kuba Perlin[3], Anna Potapenko[3], Pascal Savy[4], Sukhdeep Singh[3], Adrian Stecula[4], Ashok Thillaisundaram[3], Catherine Tong[4], Sergei Yakneen[4], Ellen D Zhong[3], Michal Zielinski[3], Augustin Žídek[3], Victor Bapst[†1], Pushmeet Kohli[†1], Max Jaderberg[†2], Demis Hassabis[†1,2], John M Jumper[†1]

[*]Contributed equally
[1]Core Contributor, Google DeepMind, London, UK
[2]Core Contributor, Isomorphic Labs, London, UK
[3]Google DeepMind, London, UK
[4]Isomorphic Labs, London, UK
[†] Jointly supervised

Corresponding author emails:
J. J. - *jumper@google.com*; D.H. - *dhcontact@google.com;* M.J. - *jaderberg@isomorphiclabs.com*

Contents

1 Notation 1

2 Data pipeline 1
- 2.1 Parsing . . . 1
- 2.2 Genetic search . . . 1
- 2.3 MSA processing . . . 2
- 2.4 Template search . . . 2
- 2.5 Training data . . . 3
  - 2.5.1 Weighted PDB dataset . . . 3
  - 2.5.2 Distillation datasets . . . 4
  - 2.5.3 Training set clustering . . . 5
  - 2.5.4 Filtering . . . 5
- 2.6 Tokenization . . . 6
- 2.7 Cropping . . . 6
  - 2.7.1 Contiguous cropping . . . 6
  - 2.7.2 Spatial Cropping . . . 6
  - 2.7.3 Spatial Interface Cropping . . . 6
- 2.8 Featurisation and model inputs . . . 7

3 Model architecture 8
- 3.1 Input embeddings . . . 9
  - 3.1.1 Input embedder . . . 9
  - 3.1.2 Relative position encoding . . . 10
- 3.2 Sequence-local atom attention . . . 11
- 3.3 MSA Module . . . 14
- 3.4 Triangle updates of the pair representation . . . 16
- 3.5 Template embedding . . . 18
- 3.6 Pairformer stack . . . 18
- 3.7 Diffusion Module . . . 19
  - 3.7.1 Diffusion Training . . . 23

4 Auxiliary heads 25
- 4.1 Mini diffusion rollout . . . 25
- 4.2 Chain permutation and symmetry resolution . . . 25
- 4.3 Model confidence prediction . . . 25
  - 4.3.1 Predicted local distance difference test (pLDDT) . . . 26
  - 4.3.2 Predicted aligned error (PAE) . . . 26
  - 4.3.3 Predicted distance error (PDE) . . . 27
  - 4.3.4 Experimentally resolved prediction . . . 28
  - 4.3.5 Confidence head architecture . . . 28
- 4.4 Distogram prediction . . . 28

5 Training and inference 29
- 5.1 Structure filters . . . 29
- 5.2 Training stages . . . 29
- 5.3 Loss . . . 29
- 5.4 Optimization . . . 29
- 5.5 Dropout . . . 30
- 5.6 Inference Setup . . . 30
- 5.7 Model selection . . . 30
- 5.8 Validation set . . . 31
- 5.9 Confidence measures and sample ranking . . . 31
  - 5.9.1 Alignment-based confidence measures . . . 31
  - 5.9.2 Clashes . . . 32
  - 5.9.3 Sample ranking . . . 32
- 5.10 Inference Time . . . 33

6 Evaluation **33**
6.1 Recent PDB evaluation set . . . 33
6.2 Evaluation set clustering . . . 34
6.3 Evaluation metrics . . . 34
6.4 Aggregation of scores . . . 34
6.5 Baselines . . . 34

7 Differences to AlphaFold 2 and AlphaFold-Multimer **35**

8 Supplemental Results **36**
8.1 Selected examples . . . 36

9 Appendix: CCD code and PDB ID tables **37**

List of Supplementary Figures

1 Sequence-local atom attention . . . 11
2 **MSA Module.** . . . 14

List of Supplementary Tables

1 Genetics databases for protein chains . . . 2
2 Genetics databases for RNA chains . . . 2
3 Sampling of training examples . . . 3
4 Weights for sampling cropping strategies . . . 6
5 Input features to the model . . . 7
6 Training stages . . . 29
7 Weighting of accuracy metrics for model selection . . . 30
8 Inference time in seconds by complex size, using 16 A100 . . . 33
9 Crystallization aids . . . 37
10 Ligand exclusion list . . . 37
11 CCD codes defining glycans . . . 37
12 Ions . . . 37
13 Standard residues . . . 38
14 Recent PDB test set with nucleic acid complexes . . . 38
15 PoseBusters V2 Common Natural Ligands . . . 38

List of Algorithms

1 `MainInferenceLoop` Main Inference Loop . . . 9
2 `InputFeatureEmbedder` Construct an initial single (1D) embedding . . . 10
3 `RelativePositionEncoding` Relative position encoding . . . 10
4 `one_hot` One-hot encoding with nearest bin . . . 11
5 `AtomAttentionEncoder` Atom attention encoder . . . 12
6 `AtomAttentionDecoder` Atom attention decoder . . . 13
7 `AtomTransformer` Atom Transformer . . . 13
8 `MsaModule` MSA Module . . . 15
9 `OuterProductMean` Outer product mean . . . 15
10 `MSAPairWeightedAveraging` MSA row-wise gated self-attention using only pair bias . . . 15
11 `Transition` Transition layer . . . 16
12 `TriangleMultiplicationOutgoing` Triangular multiplicative update using “outgoing” edges . . . 16
13 `TriangleMultiplicationIncoming` Triangular multiplicative update using “incoming” edges . . . 16
14 `TriangleAttentionStartingNode` Triangular gated self-attention around starting node . . . 17
15 `TriangleAttentionEndingNode` Triangular gated self-attention around ending node . . . 17
16 `TemplateEmbedder` Template embedder . . . 18
17 `PairformerStack` Pairformer stack . . . 19

18 SampleDiffusion Sample Diffusion . . . 20
19 CentreRandomAugmentation Centre and Random Augmentation . . . 20
20 DiffusionModule Diffusion Module . . . 21
21 DiffusionConditioning Diffusion Conditioning . . . 21
22 FourierEmbedding Fourier Embedding . . . 22
23 DiffusionTransformer Diffusion Transformer . . . 22
24 AttentionPairBias Attention with pair bias . . . 22
25 ConditionedTransitionBlock Conditioned Transition Block . . . 23
26 AdaLN Adaptive LayerNorm . . . 23
27 SmoothLDDTLoss Smooth LDDT Loss . . . 24
28 weighted_rigid_align Weighted Rigid Align . . . 25
29 expressCoordinatesInFrame Express coordinates in frame . . . 27
30 computeAlignmentError Compute alignment error . . . 27
31 ConfidenceHead Confidence head . . . 28

1 Notation

The notation used below largely follows the convention from the AlphaFold 2 paper [1], which we summarize here for convenience to the reader.

We denote the number of tokens – the fundamental sequential unit entering the Pairformer – by N_{token} (cropped during training), the number of templates used in the model by N_{templ}, the number of MSA rows used in the model by N_{msa}. Concrete values for these parameters are given in the data pipeline (section 2) and training details (section 5). On the model side, we also denote the number of blocks in Pairformer-like stacks by N_{block} (subsection 3.6), and the number of recycling iterations by N_{cycle}.

We present architectural details in Algorithms, where we use the following conventions. We use capitalized operator names when they encapsulate learnt parameters, e.g. we use Linear for a linear transformation with a weights matrix W and a bias vector $\mathbf{b}$, and LinearNoBias for the linear transformation without the bias vector. Note that when we have multiple outputs from the Linear operator at the same line of an algorithm, we imply different trainable weights for each output. A subscript index at the linear operator, e.g., LinearNoBias_a indicates also set of different trainable weights, where a selects the weight matrix to be used for that sample (used in Algorithm 31). We use LayerNorm for the layer normalization [2] operating on the channel dimensions with learnable per-channel gains and biases. We also use capitalized names for random operators, such as generators for random augmentations. For functions without parameters we use lower case operator names, e.g. sigmoid, softmax, stopgrad. We use $\odot$ for the element-wise multiplication, $\otimes$ for the outer product, $\oplus$ for the outer sum, and $\mathbf{a}^\top\mathbf{b}$ for the dot product of two vectors. Indices i, j, k always operate on the token dimension, indices l, m on the flat atom dimension, indices s, t on the sequence dimension (e.g. indexing MSA sequences or diffusion's time dimension), and index h on the attention heads dimension. The channel dimension is implicit and we type the channel-wise vectors in bold, e.g. $\mathbf{z}_{ij}$. Algorithms operate on sets of such vectors, e.g. we use $\{\mathbf{z}_{ij}\}$ to denote all pair representations. Atomic positions (i.e. vectors in Euclidean space) are specified as $\vec{\mathbf{x}} \in \mathbb{R}^3$. A full atomic structure is represented as a flat atom list $\{\vec{\mathbf{x}}_l\}$ where l indexes the atom. The mapping between tokens and their corresponding atoms is denoted $\text{i}(l) \in \mathbb{N}$ which maps the flat-atom index l to the token index i and $a = \text{token\_atom\_idx}(l)$ that maps the flat-atom index l to the within-token atom index $a \in \{1, \ldots, N_{\text{max\_atoms\_per\_token}}\}$.

2 Data pipeline

The data pipeline is the first step when running AlphaFold. It takes an input an mmCIF file and produces input features for the model.

2.1 Parsing

The data pipeline operates on mmCIF format files. In addition to parsing the `_atom_site` section, we extract basic metadata (resolution, release date, method) and some non-coordinate information (bioassembly details, chemical component details, chain names and sequences, and covalent bond information).

To simplify later code, the parser performs some basic structure cleanup. Alternative locations for atoms/residues are resolved by taking the one with the largest occupancy, MSE residues are converted to MET residues, waters are removed, and arginine naming ambiguities are fixed (ensuring NH1 is always closer to CD than NH2). The first bioassembly is then expanded, to encourage the model to predict biologically relevant complexes.

During inference a dummy mmCIF is used as input with all atom coordinates zeroed out. This can either be constructed from an mmCIF deposited in PDB[3], or from a collection of CCD code sequences and SMILES strings provided by the user.

2.2 Genetic search

We have 5 databases available to search for protein chains (see Table 1)

Jackhmmer searches use the following additional flags: -N 1 -E 0.0001 –incE 0.0001 –F1 0.0005 –F2 0.00005 –F3 0.0000005.

HHBlits searches use: -n 3 -e 0.001 -realign_max 100000 -maxfilt 100000 -min_prefilter_hits 1000 -p 20 -Z 500.

A further 3 databases are available for RNA chains (see Table 2). These databases are pre-processed by filtering to RNA entries only if necessary, then clustering with settings: --min-seq-id 0.9 -c 0.8. We search the cluster representative sequences.

Nhmmer searches use flags: -E 0.001 --incE 0.001 --rna --watson --F3 0.00005 (--F3 0.02 for sequences shorter than 50 nucleotides). RNA hit sequences are realigned to the query with hmmalign.

Table 1 | Genetics databases for protein chains

Database	Search tool	Database-specific flags	Max sequences
UniRef90 [4]	jackhmmer [5]*	--seq_limit 100000	10,000
UniProt [6]	jackhmmer	--seq_limit 500000	50,000
Uniclust30 [7] + BFD [8]	HHBlits v3.0-beta.3 [9]		None
Reduced BFD [10]	jackhmmer	--seq_limit 50000	5,000
MGnify [11]	jackhmmer	--seq_limit 50000	5,000

*All programs from the HMMER suite [12] are v3.3.

Table 2 | Genetics databases for RNA chains

Database	Clustering tool	Search tool	Max sequences
Rfam [13]	mmseqs easy-cluster [14]	nhmmer [15]	10,000
RNACentral [16]	mmseqs easy-linclust	nhmmer	10,000
Nucleotide collection [17]	mmseqs easy-cluster	nhmmer	10,000

The databases are used as follows:

- For training the models, we search UniRef90 (up to v2022_05), UniProt (v2020_05) with flag -Z 119222328, Reduced BFD or the combination Uniclust30 (v2018_08) + BFD, MGnify (up to v2022_05), Rfam (v14.9), RNACentral (v21.0), and Nucleotide collection (2023-02-23).
- For inference on recent PDB, we search UniRef90 (v2022_05), UniProt (v2021_04) with flag -Z 138515945, Reduced BFD, MGnify (v2022_05), Rfam (v14.9), RNACentral (v21.0), and Nucleotide collection (2023-02-23).
- For inference on the PoseBusters set, we search UniRef90 (v2020_01), UniProt (v2020_05), Uniclust30 (v2018_08) + BFD, and MGnify (v2018_12).

Individual MSA results are cropped to the maximum number of sequences stated above. If no hits are found, a length-1 MSA containing only the query sequence is returned.

The UniProt search result is kept separate and used to provide cross-chain genetic information, as in [18]. All other results are stacked in the order listed and deduplicated to form the main MSA. During training, the main MSA for each sequence is subsampled from size n to size $k = \mathrm{Uniform}[1, n]$ by selecting sequences to keep at random.

2.3 MSA processing

We construct an MSA with up to 16,384 rows ($N_{\mathrm{msa}} \leq$ 16,384). The first row is the query sequence, the next rows (up to 8,191) are constructed by pairing the UniProt MSA based on species, as described in the AlphaFold-Multimer paper [18]. Unlike in AlphaFold-Multimer, the rest of the MSA is constructed in a dense fashion, so if there are n paired rows then, for each chain, the last $(16384 - n)$ rows in the output MSA are the first rows from the original MSA constructed in subsection 2.2.

2.4 Template search

Template search is performed only for individual protein chains, we do not provide any multi-chain templates. The template search takes as input the deduplicated UniRef90 MSA described in subsection 2.2, which in training is cropped to the first 300 sequences. We then use hmmbuild to convert the MSA into an HMM, followed by hmmsearch with flags --noali --F1 0.1 --F2 0.1 --F3 0.1 --E 100 --incE 100 --domE 100 --incdomE 100. Hmmsearch retrieves template hits from a fasta file of all protein PDB sequences.

Template hits are filtered based on their release date. For PDB-based training datasets, the template release date must be no less than 60 days before that of the example, while for distillation sets the max template date is 2018-04-30. At inference time the max template date is our training cutoff of 2021-09-30 unless otherwise stated. We also remove templates that contain the exact query sequence with greater than 95% coverage as well as short templates less than 10 residues or covering less than 10% of the query.

Template structures are retrieved from the corresponding PDB mmCIF file, and we attempt to locate the template sequence reported by hmmsearch. This should be a subsequence of the chain returned by hmmsearch, but we fall back to checking other chains for a match, and if necessary realigning the query to the reported template chain with Kalign v.0.2.0 [19]. Once the relevant residues have been identified, that part of the template structure can be featurized similarly to the training example itself (see subsection 2.8).

Templates are sorted by e-value. At most 20 templates can be returned by our search, and the model uses up to 4 ($N_{\text{templ}} \leq 4$). At inference time we take the first 4. At training time we choose k random templates out of the available n, where $k \sim \min(\text{Uniform}[0, n], 4)$. This reduces the efficacy of simply copying the template.

Note that templates are strictly single chain; when predicting a complex, we make no attempt to select templates from the same PDB file in order to gain information about inter-chain interactions.

2.5 Training data

We train on a mixture of five structural datasets, as summarized in Table 3 below. Selecting an example for the model to train on proceeds as follows:

1. Sample a dataset according to a set of dataset weights (See the weights column of Table 3).

2. Draw an example from the selected dataset. The examples from PDB-based datasets are drawn according to our weighting sampling procedure (described in subsubsection 2.5.1 below); examples from other datasets are drawn uniformly.

3. Sample a structural crop from the selected example (described in subsection 2.7 below).

Table 3 | Sampling of training examples: Examples are drawn from a mixture of datasets with associated weight fractions (weight column). Within a dataset, examples are drawn either according to our weighted procedure or uniformly (Sampling strategy column).

Name	Description	Sampl. strategy	Weight
Weighted PDB	Ground truth PDB structures	weighted	0.5
Disordered protein PDB distillation	Proteins with unresolved residues	weighted	0.02
Protein monomer distillation	Protein monomer predictions from MGnify	uniform	0.495
Short protein monomer distillation	Protein short monomer predictions from MGnify	uniform	0.005
RNA distillation	RNA monomer predictions from Rfam	uniform	0.05
Transcription factor negatives	MGnify protein + random DNA	uniform	0.01[1]
Transcription factor positives	DNA+protein predictions from JASPAR	uniform	0.02[1]

2.5.1 Weighted PDB dataset

For training on PDB data, we sample single chains and chain-pair interfaces with a bespoke weighting meant to both redundancy-reduce (via clustering) and stratify molecule types that exist in the underlying data (via weight factors). First, a list of chains and interfaces are collected from our filtered PDB training set (filtering described in subsubsection 2.5.4), with interfaces defined as pairs of chains with minimum heavy atom (i.e. non-hydrogen) separation less than 5 Å. Each item from this dataset is then either a chain or interface and is sampled with a weight w dependent upon its item type $r \in \{\text{chain}, \text{interface}\}$, chain count per type $n_{\{\text{prot,nuc,ligand}\}}$[2], and cluster size N_{clust}:

$$w \propto \frac{\beta_r}{N_{\text{clust}}}(\alpha_{\text{prot}} * n_{\text{prot}} + \alpha_{\text{nuc}} * n_{\text{nuc}} + \alpha_{\text{ligand}} * n_{\text{ligand}}). \tag{1}$$

We chose parameters $\beta_{\text{chain}} = 0.5$, $\beta_{\text{interface}} = 1$, $\alpha_{\text{prot}} = 3$, $\alpha_{\text{nuc}} = 3$, $\alpha_{\text{ligand}} = 1$. The cluster size N_{clust} is determined from our clustering procedure, described in subsubsection 2.5.3 below.

The selected chain or interface is then used to bias the cropping procedure to select crops near the selected chain or interface (see subsection 2.7).

[1]Transcription factor training is only applied during fine tuning.
[2]For example, for a DNA or RNA chain item we would have $n_{\text{prot}} = 0, n_{\text{nuc}} = 1, n_{\text{ligand}} = 0$, and for a protein-protein interface item we would have $n_{\text{prot}} = 2, n_{\text{nuc}} = 0, n_{\text{ligand}} = 0$.

2.5.2 Distillation datasets

Other than PDB ground truth structures, our four other datasets were obtained via AlphaFold distillation, three of which coming from AlphaFold 2 predictions and one from AlphaFold 3 predictions.

1. *Protein monomer distillation*: AlphaFold 2 predictions of MGnify sequences with greater than 200 residues (same as used in AlphaFold-Multimer).

2. *Short protein monomer distillation*: AlphaFold 2 predictions of MGnify sequences between 4 and 200 residues (same inference run as for protein monomer distillation). N≈41,000,000 structures between both the long and short protein monomer distillation sets.

3. *Disordered protein PDB distillation*: AlphaFold-Multimer v2.3 predictions of PDB proteins from the training set with ground truth nucleic acids and small molecules inserted after the prediction is aligned to the ground truth protein. Predictions are filtered to having at least 40 unresolved residues, at least 60 GDT with respect to the ground truth and no atom clashes after inserting nucleic acids and small molecules. The resulting distillation structures therefore include all resolved entities in the ground truth PDB structure, with predicted coordinates for all protein residues (including unresolved ones) and ground truth coordinates for all non-protein entities. This distillation set was used to ensure predictions of unstructured regions are consistent with disordered strands seen in AlphaFoldDB predictions. Only the first bioassembly was used for this distillation set. N≈25,000 structures.

4. *RNA distillation*: We clustered Rfam (v14.9) using MMseqs2 with 90% sequence identity and 80% coverage, taking one sequence per cluster (the cluster representative). AlphaFold 3 predictions of sequences from this set were then used as an RNA distillation training set with a minimum of 10 residues and maximum average predicted distance error (PDE) of 2. N≈65,000 structures after filtering.

5. *Transcription factor positive distillation*: We constructed a dataset of positive protein-DNA examples in the following way. We first find transcription factors profiles from the JASPAR 9 database [20] which have gene ids matching those used in two high-throughput SELEX datasets [21, 22]. Next, for each profile in this filtered dataset we assign a protein sequence in two ways: 1) by taking the canonical sequence under the profile's Uniprot ID; 2) by searching across sequences used in at least one of two SELEX datasets mentioned above for the sequence with highest similarity to the Uniprot sequence and matching gene id. Sequence similarity is computed as the number of non-gap matches between aligned sequences (aligned via Kalign v2.0 [19]), divided by the minimum length of the two pre-aligned sequences. We next clustered the transcription factor sequences with 10% sequence identity and 60% coverage and randomly select 50% of clusters for training. Next we generate positive DNA sequences which should bind each transcription factor. Specifically, for each protein sequence, we sample 10 random single-stranded motifs from corresponding JASPAR profile's position frequency matrix (PFM) and remove any identical samples. For each random motif, the protein sequence, the DNA strand, and the DNA strand's reverse complement are combined to form a single distillation example. This gives a total of 16,439 protein-DNA complexes containing 1,165 unique protein sequences. Predictions on this set were made with AlphaFold 3 (trained without positive transcription factor distillation). These predictions were further filtered to only those with a minimum protein-DNA predicted distance error (PDE) of 5 Å, the majority of the predictions have much lower PDE (99% less than 3 Å).

   During training the DNA helices in this set are randomly extended with idealized DNA helices, this is done with the following steps:

   a) Remove DNA leaving atoms (OP3) from the original prediction.
   b) Sample $N_{\text{new}} \sim U\{0, 100 - N_{\text{orig}}\}$, where N_{orig} is the number of base pairs in the original prediction.
   c) Sample $N_{\text{start}} \sim U\{0, N_{\text{new}}\}$ and set $N_{\text{end}} \leftarrow N_{\text{new}} - N_{\text{start}}$.
   d) Construct two idealized B-DNA helices with random complementary base pairs of length N_{start} and N_{end}.
   e) Align the last base pairs of one of the random helices to the first base pair in the original prediction, and align the first base pair in the other random helix to the last base pair in the original prediction.
   f) If the new extended structure has any new clashes or violations, then we return the original structure without modifications. A clash is defined as having two heavy atoms within 1.5 Å, excluding neighbouring residues. A violation is defined if the bond angle differs by more than 20 degrees to the RDKit reference conformer, or if the bond lengths differ by more than 0.2 Å to the reference conformer.

6. *Transcription factor negatives distillation*: We constructed a dataset of negative protein-DNA examples. It was generated with the following steps:

a) Sample a DNA length $N \sim U\{60, 100\}$ with 0.3 probability, and from the empirical distribution of DNA lengths in PDB with 0.7 probability.

b) Randomly generate a DNA sequence with N bases and its reverse complement.

c) Generate a prediction with AlphaFold 3 for the dsDNA.

d) Sample a random protein prediction from the protein monomer distillation set.

e) Centre and randomly rotate the protein and DNA, then translate them such that the minimum distance between the two is at least $25\,\text{Å} + \mathcal{N}(2, 3)\,\text{Å}$.

2.5.3 Training set clustering

In order to reduce bias in the training and evaluation sets, clustering was performed on PDB chains and interfaces, as follows.

- Chain-based clustering occur at 40% sequence homology for proteins, 100% homology for nucleic acids, 100% homology for peptides (<10 residues) and according to CCD identity for small molecules (i.e. only identical molecules share a cluster).

- Chain-based clustering of polymers with modified residues is first done by mapping the modified residues to a standard residue using SCOP [23, 24] convention (`https://github.com/biopython/biopython/blob/5ee5e69e649dbe17baefe3919e56e60b54f8e08f/Bio/Data/SCOPData.py`). If the modified residue could not be found as a mapping key or was mapped to a value longer than a single character, it was mapped to type unknown.

- Interface-based clustering is a join on the cluster IDs of the constituent chains, such that interfaces I and J are in the same interface cluster $\mathcal{C}^{\text{interface}}$ only if their constituent chain pairs $\{I_1, I_2\}, \{J_1, J_2\}$ have the same chain cluster pairs $\{\mathcal{C}_1^{\text{chain}}, \mathcal{C}_2^{\text{chain}}\}$.

2.5.4 Filtering

The PDB data is filtered with the following constraints.

Filtering of targets:

- The structure must have been released to the PDB before the cutoff date of 2021-09-30.

- The structure must have a reported resolution of 9 Å or less.

- The maximum number of polymer chains in a considered structure is 300 for training and 1000 for evaluation.

- Any polymer chain containing fewer than 4 resolved residues is filtered out.

Filtering of bioassemblies:

- Hydrogens are removed.

- Polymer chains with all unknown residues are removed.

- Clashing chains are removed. Clashing chains are defined as those with >30% of atoms within 1.7 Å of an atom in another chain. If two chains are clashing with each other, the chain with the greater percentage of clashing atoms will be removed. If the same fraction of atoms are clashing, the chain with fewer total atoms is removed. If the chains have the same number of atoms, then the chain with the larger chain id is removed.

- For residues or small molecules with CCD codes, atoms outside of the CCD code's defined set of atom names are removed.

- Leaving atoms (ligand atom or groups of atoms that detach when bonds form) for covalent ligands are filtered out.

- Protein chains with consecutive C^{α} atoms >10 Å apart are filtered out.

- For bioassemblies with greater than 20 chains, we select a random interface token (with a centre atom <15 Å to the centre atom of a token in another chain) and select the closest 20 chains to this token based on minimum distance between any tokens centre atom.

- Crystallization aids are removed if the mmCIF method information indicates that crystallography was used (see Table 9).

2.6 Tokenization

In AlphaFold 2, the protein residue was the fundamental sequential unit entering the Evoformer single and pair stacks. In this work we have generalized the tokenization scheme so as to accommodate a wider variety of molecular types. We used the following procedure.

- A standard amino acid residue (Table 13) is represented as a single token.
- A standard nucleotide residue (Table 13) is represented as a single token.
- A modified amino acid or nucleotide residue is tokenized per-atom (i.e. N tokens for an N-atom residue)
- All ligands are tokenized per-atom

For each token we also designate a token centre atom, used in various places below:

- C^{α} for standard amino acids
- $C1'$ for standard nucleotides
- For other cases take the first and only atom as they are tokenized per-atom.

2.7 Cropping

Our cropping procedure is largely equivalent to that found in AlphaFold-Multimer [18], with the caveat that before the cropping was applied to protein residues and now it is applied to tokens. We have three main cropping strategies, described below, that are randomly selected from, with dataset-specific selection weights (see Table 4).

Table 4 | Weights for sampling cropping strategies

Dataset	Cropping weight		
	Contiguous	Spatial	Spatial interface
Weighted PDB	0.20	0.40	0.40
Disordered protein PDB complex	0.20	0.40	0.40
Monomer distillation	0.25	0.75	0.00
Short protein distillation	0.25	0.75	0.00
RNA distillation	0.25	0.75	0.00

2.7.1 Contiguous cropping

Here contiguous sequences of polymer residues and/or ligand atoms are selected for each chain. For details see section 7.2.1 and Algorithm 1 from the AlphaFold-Multimer paper [18].

2.7.2 Spatial Cropping

In this procedure, polymer residues and ligand atoms are selected that are within close spatial distance of a reference atom. The reference atom is selected at random from the set of token centre atoms (defined in subsection 2.6). For examples coming out of the Weighted PDB or Disordered protein PDB complex datasets, where a preferred chain or interface is provided (subsection 2.5), the reference atom is selected at random from token centre atoms that exist within this chain or interface.

Once the reference atom is selected, the final crop is determined via Algorithm 2 of AlphaFold-Multimer [18].

2.7.3 Spatial Interface Cropping

In this procedure, polymer residues and ligand atoms are selected that are within close spatial distance of an interface atom. The interface atom is selected at random from the set of token centre atoms (defined in subsection 2.6) with a distance under 15 Å to another chain's token centre atom. For examples coming out of the Weighted PDB or Disordered protein PDB complex datasets, where a preferred chain or interface is provided (subsection 2.5), the reference atom is selected at random from interfacial token centre atoms that exist within this chain or interface.

Once the interface atom is selected, the final crop is determined via Algorithm 2 of AlphaFold-Multimer [18].

2.8 Featurisation and model inputs

Table 5 lists the main model input features, which fall into the following categories:

- **Token features**. Composed of per-token features, such as position indexes (token_index); chain identifiers (asym_id); masks (is_protein).
- **Reference features**. Features derived from a residue, nucleotide or ligand's reference conformer. Given an input CCD code or SMILES string, the conformer is typically generated with RDKit v.2023_03_3 [25] using ETKDGv3 [26]. On error, we fall back to using the CCD ideal coordinates, or finally the representative coordinates if they are from before our training date cut-off (2021-09-30 unless otherwise stated). At the end, any atom coordinates still missing are set to zeros.
- **Msa features**. Features derived from genetics search, e.g. the MSA itself, deletion matrix, and profile.
- **Template features**. Features derived from template search, e.g. the template atom positions.
- **Bond features**. Features providing bond information, e.g. the expected locations of polymer-ligand bonds, and of intra-/inter-ligand bonds.

In the algorithms, features are referred as "**f**" with the feature name in superscript and the element indices in subscript, e.g. the one-hot encoded `msa` feature with shape $[N_{\text{msa}}, N_{\text{token}}, 32]$ is denoted as $\mathbf{f}_{si}^{\text{msa}} \in \mathbb{N}^{32}$

Table 5 | Input features to the model. Feature dimensions: N_{token} is the number of tokens, N_{msa} is the number of MSA sequences, N_{templ} is the number of templates, N_{atom} is the number of atoms, N_{chains} is the number of chains, N_{bonds} is the number of bonds, N_{perm} the number of atom permutations.

Feature & Shape	Description
residue_index $[N_{\text{token}}]$	Residue number in the token's original input chain.
token_index $[N_{\text{token}}]$	Token number. Increases monotonically; does not restart at 1 for new chains.
asym_id $[N_{\text{token}}]$	Unique integer for each distinct chain.
entity_id $[N_{\text{token}}]$	Unique integer for each distinct sequence.
sym_id $[N_{\text{token}}]$	Unique integer within chains of this sequence. E.g. if chains A, B and C share a sequence but D does not, their sym_ids would be [0, 1, 2, 0].
restype $[N_{\text{token}}, 32]$	One-hot encoding of the sequence. 32 possible values: 20 amino acids + unknown, 4 RNA nucleotides + unknown, 4 DNA nucleotides + unknown, and gap. Ligands represented as "unknown amino acid".
is_protein / rna / dna / ligand $[N_{\text{token}}]$	4 masks indicating the molecule type of a particular token.
ref_pos $[N_{\text{atom}}, 3]$	Atom positions in the reference conformer, with a random rotation and translation applied. Atom positions are given in Å.
ref_mask $[N_{\text{atom}}]$	Mask indicating which atom slots are used in the reference conformer.
ref_element $[N_{\text{atom}}, 128]$	One-hot encoding of the element atomic number for each atom in the reference conformer, up to atomic number 128.
ref_charge $[N_{\text{atom}}]$	Charge for each atom in the reference conformer.

Feature & Shape	Description
ref_atom_name_chars $[N_{\text{atom}}, 4, 64]$	One-hot encoding of the unique atom names in the reference conformer. Each character is encoded as $\text{ord}(c) - 32$, and names are padded to length 4.
ref_space_uid $[N_{\text{atom}}]$	Numerical encoding of the chain id and residue index associated with this reference conformer. Each (chain id, residue index) tuple is assigned an integer on first appearance.
msa $[N_{\text{msa}}, N_{\text{token}}, 32]$	One-hot encoding of the processed MSA, using the same classes as restype.
has_deletion $[N_{\text{msa}}, N_{\text{token}}]$	Binary feature indicating if there is a deletion to the left of each position in the MSA.
deletion_value $[N_{\text{msa}}, N_{\text{token}}]$	Raw deletion counts (the number of deletions to the left of each MSA position) are transformed to $[0, 1]$ using $\frac{2}{\pi} \arctan \frac{d}{3}$.
profile $[N_{\text{token}}, 32]$	Distribution across restypes in the main MSA. Computed before MSA processing (subsection 2.3).
deletion_mean $[N_{\text{token}}]$	Mean number of deletions at each position in the main MSA. Computed before MSA processing (subsection 2.3).
template_restype $[N_{\text{templ}}, N_{\text{token}}]$	One-hot encoding of the template sequence, see restype.
template_pseudo_beta_mask $[N_{\text{templ}}, N_{\text{token}}]$	Mask indicating if the C^{β} (C^{α} for glycine) has coordinates for the template at this residue.
template_backbone_frame_mask $[N_{\text{templ}}, N_{\text{token}}]$	Mask indicating if coordinates exist for all atoms required to compute the backbone frame (used in the template_unit_vector feature).
template_distogram $[N_{\text{templ}}, N_{\text{token}}, N_{\text{token}}, 39]$	A one-hot pairwise feature indicating the distance between C^{β} atoms (C^{α} for glycine). Pairwise distances are discretized into 38 bins of equal width between 3.25 Å and 50.75 Å; one more bin contains any larger distances.
template_unit_vector $[N_{\text{templ}}, N_{\text{token}}, N_{\text{token}}, 3]$	The unit vector of the displacement of the C^{α} atom of all residues within the local frame of each residue. Local frames are computed as in [1].
token_bonds $[N_{\text{token}}, N_{\text{token}}]$	A 2D matrix indicating if there is a bond between any atom in token i and token j, restricted to just polymer-ligand and ligand-ligand bonds and bonds less than 2.4 Å during training.

3 Model architecture

The model architecture is broadly based on AlphaFold 2; however, we made a number of changes to enable the model to predict a wider range of molecules than proteins and also to further increase the accuracy in protein structure predictions.

The model is a conditional diffusion model, where unlike most other diffusion models most of the computation is happening in the conditioning. The conditioning part of the model is similar in overall architecture to the trunk (Template Module, MSA Module, and Pairformer) of AlphaFold 2, with a number of key differences. The algorithm corresponding to the architecture is shown in Algorithm 1 and in **Main Article Fig. 1d**. We introduce a more general tokenization scheme, where each amino acid residue corresponds to one token as in AlphaFold 2 and each nucleotide corresponds to one token, while for other molecules we encode each heavy atom as its own token. In order to get the initial trunk input feature we have a more sophisticated input feature embedder that performs attention over all atoms

in order to encode the information about the chemical structure of all the molecules, leading to a single representation representing all the tokens.

Given the input features, we build a pair representation in a manner similar to AlphaFold 2. This pair representation and the single representation are then fed into the main part of the conditioning network, which is recycled multiple times.

The main part of the conditioning network consists of a TemplateEmbedder in a similar style to AlphaFold 2 which encodes information about provided templates into the pair representation. This is followed by an MSA Module that extracts information from the MSA and encodes it into the pair representation. Here we use MSAs for both protein sequences as well as RNA sequences. The resulting pair representation is then used as an input for the main PairformerStack, which also receives the single representation as an input and processes the representation further forming the main loop of the model.

The resulting single and pair embeddings are then used to condition a diffusion process. Here the diffusion is parametrized by a Diffusion Module, which is a considerably cheaper sub-network that encodes a single denoising step. Notably it scales quadratically in the number of tokens rather than cubically. The resulting output structure from the Diffusion Module is then passed to a confidence head, which uses the pair and single representation together with the structure to provide confidence measures. The components of the model are described in the subsequent sections.

Algorithm 1 Main Inference Loop

def $\mathrm{MainInferenceLoop}(\{\mathbf{f}^*\}, N_{\text{cycle}} = 4, c_s = 384, c_z = 128):$

$\{\mathbf{s}_i^{\text{inputs}}\} = \mathrm{InputFeatureEmbedder}(\{\mathbf{f}^*\})$

$\mathbf{s}_i^{\text{init}} = \mathrm{LinearNoBias}(\mathbf{s}_i^{\text{inputs}})$ $\qquad \mathbf{s}_i^{\text{init}} \in \mathbb{R}^{c_s}$

$\mathbf{z}_{ij}^{\text{init}} = \mathrm{LinearNoBias}(\mathbf{s}_i^{\text{inputs}}) + \mathrm{LinearNoBias}(\mathbf{s}_j^{\text{inputs}})$ $\qquad \mathbf{z}_{ij}^{\text{init}} \in \mathbb{R}^{c_z}$

$\mathbf{z}_{ij}^{\text{init}} \mathrel{+}= \mathrm{RelativePositionEncoding}(\{\mathbf{f}^*\})$

$\mathbf{z}_{ij}^{\text{init}} \mathrel{+}= \mathrm{LinearNoBias}(\mathrm{f}_{ij}^{\text{token\_bonds}})$

$\{\hat{\mathbf{z}}_{ij}\}, \{\hat{\mathbf{s}}_i\} = \mathbf{0}, \mathbf{0}$

for all $c \in [1, \ldots, N_{\text{cycle}}]$ **do**

  $\mathbf{z}_{ij} = \mathbf{z}_{ij}^{\text{init}} + \mathrm{LinearNoBias}(\mathrm{LayerNorm}(\hat{\mathbf{z}}_{ij}))$ $\qquad \mathbf{z}_{ij} \in \mathbb{R}^{c_z}$

  $\{\mathbf{z}_{ij}\} \mathrel{+}= \mathrm{TemplateEmbedder}(\{\mathbf{f}^*\}, \{\mathbf{z}_{ij}\})$

  $\{\mathbf{z}_{ij}\} \mathrel{+}= \mathrm{MsaModule}(\{\mathbf{f}_{Si}^{\text{msa}}\}, \{\mathbf{z}_{ij}\}, \{\mathbf{s}_i^{\text{inputs}}\})$

  $\mathbf{s}_i = \mathbf{s}_i^{\text{init}} + \mathrm{LinearNoBias}(\mathrm{LayerNorm}(\hat{\mathbf{s}}_i))$ $\qquad \mathbf{s}_i \in \mathbb{R}^{c_s}$

  $\{\mathbf{s}_i\}, \{\mathbf{z}_{ij}\} = \mathrm{PairformerStack}(\{\mathbf{s}_i\}, \{\mathbf{z}_{ij}\})$

  $\{\hat{\mathbf{s}}_i\}, \{\hat{\mathbf{z}}_{ij}\} \leftarrow \{\mathbf{s}_i\}, \{\mathbf{z}_{ij}\}$

end for

$\{\vec{\mathbf{x}}_l^{\text{pred}}\} = \mathrm{SampleDiffusion}(\{\mathbf{f}^*\}, \{\mathbf{s}_i^{\text{inputs}}\}, \{\mathbf{s}_i\}, \{\mathbf{z}_{ij}\})$

$\{\mathbf{p}_l^{\text{plddt}}\}, \{\mathbf{p}_{ij}^{\text{pae}}\}, \{\mathbf{p}_{ij}^{\text{pde}}\}, \{\mathbf{p}_l^{\text{resolved}}\} = \mathrm{ConfidenceHead}(\{\mathbf{s}_i^{\text{inputs}}\}, \{\mathbf{s}_i\}, \{\mathbf{z}_{ij}\}, \{\vec{\mathbf{x}}_l^{\text{pred}}\})$

$\mathbf{p}_{ij}^{\text{distogram}} = \mathrm{DistogramHead}(\mathbf{z}_{ij})$

return $\{\vec{\mathbf{x}}_l^{\text{pred}}\}, \{\mathbf{p}_l^{\text{plddt}}\}, \{\mathbf{p}_{ij}^{\text{pae}}\}, \{\mathbf{p}_{ij}^{\text{pde}}\}, \{\mathbf{p}_l^{\text{resolved}}\}, \{\mathbf{p}_{ij}^{\text{distogram}}\}$

3.1 Input embeddings

Any bonds provided by the user (via the token_bonds feature) are linearly embedded in Algorithm 1, the embedding of the other user inputs, along with the RDKit reference conformer are described below.

3.1.1 Input embedder

The residue type, reference conformer and MSA summary features (profile and deletion_mean) are embedded in Algorithm 2. The reference conformer is embedded in a permutation invariant way using the AtomAttentionEncoder (Algorithm 5).

Algorithm 2 Construct an initial 1D embedding

def InputFeatureEmbedder($\{\mathbf{f}^*\}$) :

# Embed per-atom features.

$\{\mathbf{a}_i\}, \_, \_, \_ = \text{AtomAttentionEncoder}(\{\mathbf{f}^*\}, \emptyset, \emptyset, \emptyset, c_{\text{atom}} = 128, c_{\text{atompair}} = 16, c_{\text{token}} = 384)$

# Concatenate the per-token features.

$\mathbf{s}_i = \text{concat}(\mathbf{a}_i, \mathbf{f}_i^{\text{restype}}, \mathbf{f}_i^{\text{profile}}, \text{f}_i^{\text{deletion\_mean}})$

return $\{\mathbf{s}_i\}$

3.1.2 Relative position encoding

As in AlphaFold 2 and AlphaFold-Multimer, relative encodings are used to break symmetries across identical residues ($\mathbf{a}_{ij}^{\text{rel\_pos}}$) and chains ($\mathbf{a}_{ij}^{\text{rel\_chain}}$). In AlphaFold 3 we introduce a relative token encoding, applied to tokens within the same residue (see Algorithm 3). The relative position and token indices are clipped between $[r_{\text{min}}, r_{\text{max}}]$, with $r_{\text{max}} = 32$. Relative chain indices are clipped between $[s_{\text{min}}, s_{\text{max}}]$, with $s_{\text{max}} = 2$.

Algorithm 3 Relative position encoding

def RelativePositionEncoding($\{\mathbf{f}^*\}, r_{\text{max}}, s_{\text{max}}, c_z = 128$) :

$b_{ij}^{\text{same\_chain}} = (\text{f}_i^{\text{asym\_id}} == \text{f}_j^{\text{asym\_id}})$

$b_{ij}^{\text{same\_residue}} = (\text{f}_i^{\text{residue\_index}} == \text{f}_j^{\text{residue\_index}})$

$b_{ij}^{\text{same\_entity}} = (\text{f}_i^{\text{entity\_id}} == \text{f}_j^{\text{entity\_id}})$

$$d_{ij}^{\text{residue}} = \begin{cases} \text{clip}(\text{f}_i^{\text{residue\_index}} - \text{f}_j^{\text{residue\_index}} + r_{\text{max}}, 0, 2 \cdot r_{\text{max}}) & \text{if } b_{ij}^{\text{same\_chain}} \\ 2 \cdot r_{\text{max}} + 1 & \text{else} \end{cases} \qquad d_{ij}^{\text{residue}} \in \mathbb{N}$$

$\mathbf{a}_{ij}^{\text{rel\_pos}} = \text{one\_hot}(d_{ij}^{\text{residue}}, [0, \ldots, 2 \cdot r_{\text{max}} + 1])$

$$d_{ij}^{\text{token}} = \begin{cases} \text{clip}(\text{f}_i^{\text{token\_index}} - \text{f}_j^{\text{token\_index}} + r_{\text{max}}, 0, 2 \cdot r_{\text{max}}) & \text{if } b_{ij}^{\text{same\_chain}} \text{ and } b_{ij}^{\text{same\_residue}} \\ 2 \cdot r_{\text{max}} + 1 & \text{else} \end{cases} \qquad d_{ij}^{\text{token}} \in \mathbb{N}$$

$\mathbf{a}_{ij}^{\text{rel\_token}} = \text{one\_hot}(d_{ij}, [0, \ldots, 2 \cdot r_{\text{max}} + 1])$

$$d_{ij}^{\text{chain}} = \begin{cases} \text{clip}(\text{f}_i^{\text{sym\_id}} - \text{f}_j^{\text{sym\_id}} + s_{\text{max}}, 0, 2 \cdot s_{\text{max}}) & \text{if not } b_{ij}^{\text{same\_chain}} \\ 2 \cdot s_{\text{max}} + 1 & \text{else} \end{cases} \qquad d_{ij}^{\text{chain}} \in \mathbb{N}$$

$\mathbf{a}_{ij}^{\text{rel\_chain}} = \text{one\_hot}(d_{ij}^{\text{chain}}, [0, \ldots, 2 \cdot s_{\text{max}} + 1])$

$\mathbf{p}_{ij} = \text{LinearNoBias}(\text{concat}([\mathbf{a}_{ij}^{\text{rel\_pos}}, \mathbf{a}_{ij}^{\text{rel\_token}}, b_{ij}^{\text{same\_entity}}, \mathbf{a}_{ij}^{\text{rel\_chain}}]))$ $\qquad \mathbf{p}_{ij} \in \mathbb{R}^{c_z}$

return $\{\mathbf{p}_{ij}\}$

Algorithm 4 One-hot encoding with nearest bin

def one_hot$(x, \mathbf{v}_{\text{bins}})$: $x \in \mathbb{R}, \quad \mathbf{v}_{\text{bins}} \in \mathbb{R}^{N_{\text{bins}}}$

1: $\mathbf{p} = \mathbf{0}$ $\mathbf{p} \in \mathbb{R}^{N_{\text{bins}}}$

2: $b = \arg\min(|x - \mathbf{v}_{\text{bins}}|)$

3: $p_b = 1$

4: **return** $\mathbf{p}$

3.2 Sequence-local atom attention

The "sequence-local atom attention" represents the whole structure as a flat list of atoms and allows all atoms to "talk" directly to each other within a certain sequence neighbourhood. E.g., each subset of 32 atoms attends to the subset of the nearby 128 atoms (nearby in the sequence space). This gives the network the capacity to learn general rules about local atom constellations, independently from the coarse-grained tokenization where each standard residue is represented with a single token only.

The restriction to a certain sequence neighbourhood is sub-optimal, but was necessary to keep the memory and compute costs within reasonable bounds. The resulting attention pattern is equivalent to computing the full affinity matrix and applying a neighbourhood mask that contains the rectangular blocks along the diagonal (see Suppl. Fig. 1).

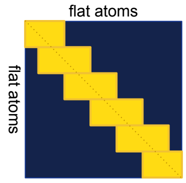

Supplementary Figure 1 | Sequence-local atom attention. Each subset of atoms (rows) attends to a larger subset of atoms (columns). The blue area depicts the theoretical full $N_{\text{atoms}} \times N_{\text{atoms}}$ attention matrix. The yellow rectangles represent the attentions that are realized.

The conversion of the atom indexing by token index $i \in N_{\text{tokens}}$ and atom name $a \in \mathcal{S}_{\text{atom names}}$, e. g. $\{\vec{\mathbf{x}}_i^a\}$ to a flat indexing with atom index $l \in N_{\text{atoms}}$ is denoted by the mapping of flat atom index to the token index $i = \text{tok\_idx}(l)$.

Algorithm 5 Atom attention encoder

def $\text{AtomAttentionEncoder}(\{\mathbf{f}^*\}, \{\mathbf{r}_l\}, \{\mathbf{s}_i^{\text{trunk}}\}, \{\mathbf{z}_{ij}\}, c_{\text{atom}}, c_{\text{atompair}}, c_{\text{token}}):$

# Create the atom single conditioning: Embed per-atom meta data

$\mathbf{c}_l = \text{LinearNoBias}(\text{concat}(\vec{\mathbf{f}}_l^{\text{ref\_pos}}, \text{f}_l^{\text{ref\_charge}}, \text{f}_l^{\text{ref\_mask}}, \mathbf{f}_l^{\text{ref\_element}}, \mathbf{f}_l^{\text{ref\_atom\_name\_chars}}))$ $\quad \mathbf{c}_l \in \mathbb{R}^{c_{\text{atom}}}$, $l \in \{1, \ldots, N_{\text{atoms}}\}$

# Embed offsets between atom reference positions

$\vec{\mathbf{d}}_{lm} = \vec{\mathbf{f}}_l^{\text{ref\_pos}} - \vec{\mathbf{f}}_m^{\text{ref\_pos}}$ $\quad \vec{\mathbf{d}}_{lm} \in \mathbb{R}^3$

$v_{lm} = (\text{f}_l^{\text{ref\_space\_uid}} == \text{f}_m^{\text{ref\_space\_uid}})$ $\quad v_{lm} \in \mathbb{R}$

$\mathbf{p}_{lm} = \text{LinearNoBias}(\vec{\mathbf{d}}_{lm}) \cdot v_{lm}$ $\quad \mathbf{p}_{lm} \in \mathbb{R}^{c_{\text{atompair}}}$

# Embed pairwise inverse squared distances, and the valid mask.

$\mathbf{p}_{lm} \mathrel{+}= \text{LinearNoBias}\left(1/\left(1 + \|\vec{\mathbf{d}}_{lm}\|^2\right)\right) \cdot v_{lm}$

$\mathbf{p}_{lm} \mathrel{+}= \text{LinearNoBias}(v_{lm}) \cdot v_{lm}$

# Initialise the atom single representation as the single conditioning.

$\mathbf{q}_l = \mathbf{c}_l$ $\quad \mathbf{q}_l \in \mathbb{R}^{c_{\text{atom}}}$

# If provided, add trunk embeddings and noisy positions.

if $\{\mathbf{r}_l\} \neq \emptyset$ **then**

  # Broadcast the single and pair embedding from the trunk.

  $\mathbf{c}_l \mathrel{+}= \text{LinearNoBias}(\text{LayerNorm}(\mathbf{s}^{\text{trunk}}_{\text{tok\_idx}(l)}))$

  $\mathbf{p}_{lm} \mathrel{+}= \text{LinearNoBias}(\text{LayerNorm}(\mathbf{z}_{\text{tok\_idx}(l)\,\text{tok\_idx}(m)}))$

  # Add the noisy positions.

  $\mathbf{q}_l \mathrel{+}= \text{LinearNoBias}\,(\mathbf{r}_l)$

end if

# Add the combined single conditioning to the pair representation.

$\mathbf{p}_{lm} \mathrel{+}= \text{LinearNoBias}(\text{relu}(\mathbf{c}_l)) + \text{LinearNoBias}(\text{relu}(\mathbf{c}_m))$

# Run a small MLP on the pair activations.

$\mathbf{p}_{lm} \mathrel{+}= \text{LinearNoBias}(\text{relu}(\text{LinearNoBias}(\text{relu}(\text{LinearNoBias}(\text{relu}(\mathbf{p}_{lm}))))))$

# Cross attention transformer.

$\{\mathbf{q}_l\} = \text{AtomTransformer}(\{\mathbf{q}_l\}, \{\mathbf{c}_l\}, \{\mathbf{p}_{lm}\}, N_{\text{block}} = 3, N_{\text{head}} = 4)$

# Aggregate per-atom representation to per-token representation

$\mathbf{a}_i = \underset{\substack{l \in \{1,\ldots,N_{\text{atoms}}\} \\ \text{tok\_idx}(l) = i}}{\text{mean}} (\text{relu}(\text{LinearNoBias}(\mathbf{q}_l)))$ $\quad \mathbf{a}_i \in \mathbb{R}^{c_{\text{token}}}$

$\mathbf{q}_l^{\text{skip}}, \mathbf{c}_l^{\text{skip}}, \mathbf{p}_{lm}^{\text{skip}} = \mathbf{q}_l, \mathbf{c}_l, \mathbf{p}_{lm}$

return $\{\mathbf{a}_i\}, \{\mathbf{q}_l^{\text{skip}}\}, \{\mathbf{c}_l^{\text{skip}}\}, \{\mathbf{p}_{lm}^{\text{skip}}\}$

Algorithm 6 Atom attention decoder

def $\text{AtomAttentionDecoder}(\{\mathbf{a}_i\}, \{\mathbf{q}_l^{\text{skip}}\}, \{\mathbf{c}_l^{\text{skip}}\}, \{\mathbf{p}_{lm}^{\text{skip}}\})$:

# Broadcast per-token activiations to per-atom activations and add the skip connection

1: $\mathbf{q}_l = \text{LinearNoBias}(\mathbf{a}_{\text{tok\_idx}(l)}) + \mathbf{q}_l^{\text{skip}}$

# Cross attention transformer.

2: $\{\mathbf{q}_l\} = \text{AtomTransformer}(\{\mathbf{q}_l\}, \{\mathbf{c}_l^{\text{skip}}\}, \{\mathbf{p}_{lm}^{\text{skip}}\}, N_{\text{block}} = 3, N_{\text{head}} = 4)$

# Map to positions update.

3: $\mathbf{r}_l^{\text{update}} = \text{LinearNoBias}(\text{LayerNorm}(\mathbf{q}_l))$

4: **return** $\{\mathbf{r}_l^{\text{update}}\}$

Algorithm 7 Atom Transformer

def $\text{AtomTransformer}(\{\mathbf{q}_l\}, \{\mathbf{c}_l\}, \{\mathbf{p}_{lm}\}, N_{\text{block}} = 3, N_{\text{head}}, N_{\text{queries}} = 32, N_{\text{keys}} = 128, \mathcal{S}_{\text{subset centres}} = \{15.5, 47.5, 79.5, \ldots\})$:

# sequence-local atom attention is equivalent to self attention within rectangular blocks along the diagonal.

1: $\beta_{lm} = \begin{cases} 0 & \text{if } |l-c| < N_{\text{queries}}/2 \wedge |m-c| < N_{\text{keys}}/2 \quad \forall\, c \in \mathcal{S}_{\text{subset centres}} \\ -10^{10} & \text{else} \end{cases}$

2: $\{\mathbf{q}_l\} = \text{DiffusionTransformer}(\{\mathbf{q}_l\}, \{\mathbf{c}_l\}, \{\mathbf{p}_{lm}\}, \{\beta_{lm}\}, N_{\text{block}}, N_{\text{head}})$

3: **return** $\{\mathbf{q}_l\}$

3.3 MSA Module

The MSA Module (Suppl. Fig. 2, Algorithm 8) in AlphaFold 3 fullfills a similar role to the Extra MSA Stack in AlphaFold 2 and hence has a fairly similar network architecture to AlphaFold-Multimer in the block. It samples a new iid random subset of the MSA for each recycling iteration, the MSA sequences and input features then get embedded into representation $\mathbf{m}_{si}$ for each token in each sequence in the msa. The Network architecture of the MSA Module then consists of 4 homogeneous blocks which repeatedly process and combine the pair representation $\mathbf{z}_{ij}$ and the msa. The final pair representation then gets passed on to the Pairformer Stack.

The overall structure of the block is very similar to the Pairformer Stack, where the MSA representation fullfills a role similar to the single representation. The individual blocks here are similar to the extra MSA stack in AlphaFold 2, the difference here is that the attention is independently performed for each row of the MSA and that attention weights are entirely projected from the pair representation, i.e. there is no key-query based attention. This also means that each row of the MSA combines information via attention in the same way, this reduces computation and memory usage in the attention. The MSA attention layer employs the same gating mechanism as the other attention layers. Otherwise this part of the model works the same as AlphaFold 2, meaning the pair representation gets passed through Triangular Multiplicative Update and Triangular self-attention layers and a transition block. In all the transition blocks we use SwiGLU instead of ReLU.

Conceptually a difference to AlphaFold 2 is that here we do not combine information across different rows of the MSA directly, but rather all information has to flow via the pair representation. The motivation behind this is that the pair representation should contain as much information as possible about the proteins or nucleic acids as it forms the backbone for the rest of the network.

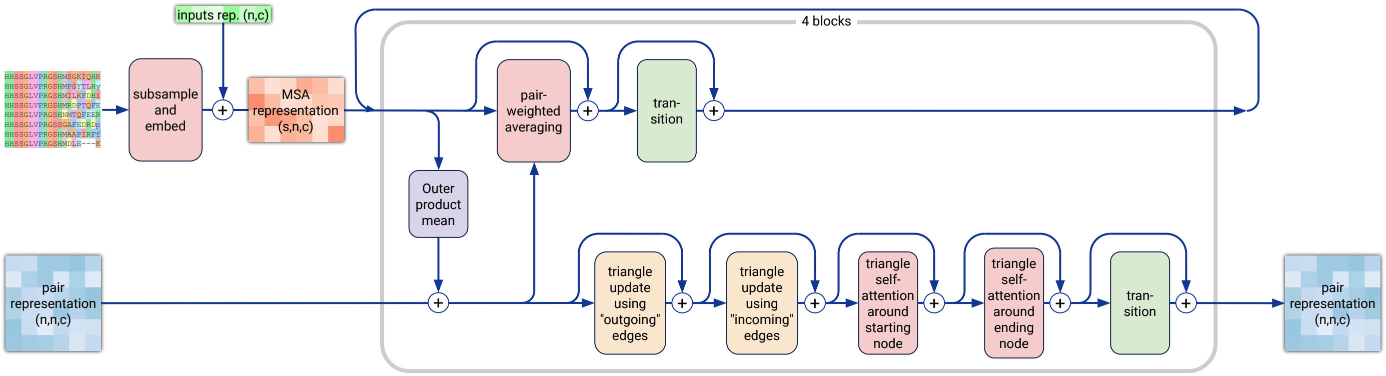

Supplementary Figure 2 | MSA Module.

Algorithm 8 MSA Module

def $\mathrm{MsaModule}(\{\mathbf{f}^*\}, \{\mathbf{z}_{ij}\}, \{\mathbf{s}_i^{\text{inputs}}\}, N_{\text{block}} = 4, c_{\text{m}} = 64):$

$\mathbf{m}_{Si} = \mathrm{concat}(\mathbf{f}_{Si}^{\text{msa}}, \mathrm{f}_{Si}^{\text{has\_deletion}}, \mathrm{f}_{Si}^{\text{deletion\_value}})$

$\{s\} = \mathrm{SampleRandomWithoutReplacement}(\{S\})$

$\mathbf{m}_{si} \leftarrow \mathrm{LinearNoBias}(\mathbf{m}_{si})$ $\qquad \mathbf{m}_{si} \in \mathbb{R}^{c_{\text{m}}}$

$\mathbf{m}_{si} \mathrel{+}= \mathrm{LinearNoBias}(\{\mathbf{s}_i^{\text{inputs}}\})$

for all $l \in [1, \ldots, N_{\text{block}}]$ **do**

# Communication

$\quad \{\mathbf{z}_{ij}\} \mathrel{+}= \mathrm{OuterProductMean}(\{\mathbf{m}_{si}\})$

# MSA stack

$\quad \{\mathbf{m}_{si}\} \mathrel{+}= \mathrm{DropoutRowwise}_{0.15}(\mathrm{MSAPairWeightedAveraging}(\{\mathbf{m}_{si}\}, \{\mathbf{z}_{ij}\}, c = 8))$

$\quad \{\mathbf{m}_{si}\} \mathrel{+}= \mathrm{Transition}(\{\mathbf{m}_{si}\})$

# Pair stack

$\quad \{\mathbf{z}_{ij}\} \mathrel{+}= \mathrm{DropoutRowwise}_{0.25}(\mathrm{TriangleMultiplicationOutgoing}(\{\mathbf{z}_{ij}\}))$

$\quad \{\mathbf{z}_{ij}\} \mathrel{+}= \mathrm{DropoutRowwise}_{0.25}(\mathrm{TriangleMultiplicationIncoming}(\{\mathbf{z}_{ij}\}))$

$\quad \{\mathbf{z}_{ij}\} \mathrel{+}= \mathrm{DropoutRowwise}_{0.25}(\mathrm{TriangleAttentionStartingNode}(\{\mathbf{z}_{ij}\}))$

$\quad \{\mathbf{z}_{ij}\} \mathrel{+}= \mathrm{DropoutColumnwise}_{0.25}(\mathrm{TriangleAttentionEndingNode}(\{\mathbf{z}_{ij}\}))$

$\quad \{\mathbf{z}_{ij}\} \mathrel{+}= \mathrm{Transition}(\{\mathbf{z}_{ij}\})$

end for

return $\{\mathbf{z}_{ij}\}$

Algorithm 9 Outer product mean

def $\mathrm{OuterProductMean}(\{\mathbf{m}_{si}\}, c = 32, c_{\text{z}} = 128):$

$\mathbf{m}_{si} \leftarrow \mathrm{LayerNorm}(\mathbf{m}_{si})$

$\mathbf{a}_{si}, \mathbf{b}_{si} = \mathrm{LinearNoBias}(\mathbf{m}_{si})$ $\qquad \mathbf{a}_{si}, \mathbf{b}_{si} \in \mathbb{R}^{c}$

$\mathbf{o}_{ij} = \mathrm{flatten}\left(\mathrm{mean}_s(\mathbf{a}_{si} \otimes \mathbf{b}_{sj})\right)$ $\qquad \mathbf{o}_{ij} \in \mathbb{R}^{c \cdot c}$

$\mathbf{z}_{ij} = \mathrm{Linear}(\mathbf{o}_{ij})$ $\qquad \mathbf{z}_{ij} \in \mathbb{R}^{c_{\text{z}}}$

return $\{\mathbf{z}_{ij}\}$

Algorithm 10 MSA pair weighted averaging with gating

def $\mathrm{MSAPairWeightedAveraging}(\{\mathbf{m}_{si}\}, \{\mathbf{z}_{ij}\}, c = 32, N_{\text{head}} = 8):$ $\qquad \mathbf{m}_{si} \in \mathbb{R}^{c_{\text{m}}}$

# Input projections

$\mathbf{m}_{si} \leftarrow \mathrm{LayerNorm}(\mathbf{m}_{si})$

$\mathbf{v}_{si}^h = \mathrm{LinearNoBias}(\mathbf{m}_{si})$ $\qquad \mathbf{v}_{si}^h \in \mathbb{R}^{c},\ h \in \{1, \ldots, N_{\text{head}}\}$

$b_{ij}^h = \mathrm{LinearNoBias}(\mathrm{LayerNorm}(\mathbf{z}_{ij}))$

$\mathbf{g}_{si}^h = \mathrm{sigmoid}\left(\mathrm{LinearNoBias}(\mathbf{m}_{si})\right)$ $\qquad \mathbf{g}_{si}^h \in \mathbb{R}^{c}$

# Weighted average with gating

$w_{ij}^h = \mathrm{softmax}_j\left(b_{ij}^h\right)$

$\mathbf{o}_{si}^h = \mathbf{g}_{si}^h \odot \sum_j w_{ij}^h \mathbf{v}_{sj}^h$

# Output projection

$\tilde{\mathbf{m}}_{si} = \mathrm{LinearNoBias}\left(\mathrm{concat}_h(\mathbf{o}_{si}^h)\right)$ $\qquad \tilde{\mathbf{m}}_{si} \in \mathbb{R}^{c_{\text{m}}}$

return $\{\tilde{\mathbf{m}}_{si}\}$

Algorithm 11 Transition layer

def Transition($\mathbf{x}, n = 4$) : $\mathbf{x} \in \mathbb{R}^c$

$\mathbf{x} \leftarrow \text{LayerNorm}(\mathbf{x})$

$\mathbf{a} = \text{LinearNoBias}(\mathbf{x})$ $\mathbf{a} \in \mathbb{R}^{n \cdot c}$

$\mathbf{b} = \text{LinearNoBias}(\mathbf{x})$ $\mathbf{b} \in \mathbb{R}^{n \cdot c}$

$\mathbf{x} \leftarrow \text{LinearNoBias}(\text{swish}(\mathbf{a}) \odot \mathbf{b})$ $\mathbf{x} \in \mathbb{R}^c$

return $\mathbf{x}$

3.4 Triangle updates of the pair representation

AlphaFold 3 uses the same update / attention scheme for the pair representation as AlphaFold 2 which establishes a direct communication between the edges that connect 3 nodes (interpreting the pair representation as edge features of a fully connected graph where the tokens are the nodes). This allows the network to easily detect inconsistencies in its current belief about the spatial relationship and to update them accordingly. For more details, see [1].

Algorithm 12 Triangular multiplicative update using "outgoing" edges

def TriangleMultiplicationOutgoing($\{\mathbf{z}_{ij}\}, c = 128$) : $\mathbf{z}_{ij} \in c_z$

$\mathbf{z}_{ij} \leftarrow \text{LayerNorm}(\mathbf{z}_{ij})$

$\mathbf{a}_{ij}, \mathbf{b}_{ij} = \text{sigmoid}\left(\text{LinearNoBias}(\mathbf{z}_{ij})\right) \odot \text{LinearNoBias}(\mathbf{z}_{ij})$ $\mathbf{a}_{ij}, \mathbf{b}_{ij} \in \mathbb{R}^c$

$\mathbf{g}_{ij} = \text{sigmoid}\left(\text{LinearNoBias}(\mathbf{z}_{ij})\right)$ $\mathbf{g}_{ij} \in \mathbb{R}^{c_z}$

$\tilde{\mathbf{z}}_{ij} = \mathbf{g}_{ij} \odot \text{LinearNoBias}(\text{LayerNorm}(\sum_k \mathbf{a}_{ik} \odot \mathbf{b}_{jk}))$ $\tilde{\mathbf{z}}_{ij} \in \mathbb{R}^{c_z}$

return $\{\tilde{\mathbf{z}}_{ij}\}$

Algorithm 13 Triangular multiplicative update using "incoming" edges (differences to Algorithm 12 highlighted)

def TriangleMultiplicationIncoming($\{\mathbf{z}_{ij}\}, c = 128$) : $\mathbf{z}_{ij} \in c_z$

$\mathbf{z}_{ij} \leftarrow \text{LayerNorm}(\mathbf{z}_{ij})$

$\mathbf{a}_{ij}, \mathbf{b}_{ij} = \text{sigmoid}\left(\text{LinearNoBias}(\mathbf{z}_{ij})\right) \odot \text{LinearNoBias}(\mathbf{z}_{ij})$ $\mathbf{a}_{ij}, \mathbf{b}_{ij} \in \mathbb{R}^c$

$\mathbf{g}_{ij} = \text{sigmoid}\left(\text{LinearNoBias}(\mathbf{z}_{ij})\right)$ $\mathbf{g}_{ij} \in \mathbb{R}^{c_z}$

$\tilde{\mathbf{z}}_{ij} = \mathbf{g}_{ij} \odot \text{LinearNoBias}(\text{LayerNorm}(\sum_k \mathbf{a}_{ki} \odot \mathbf{b}_{kj}))$ $\tilde{\mathbf{z}}_{ij} \in \mathbb{R}^{c_z}$

return $\{\tilde{\mathbf{z}}_{ij}\}$

Algorithm 14 Triangular gated self-attention around starting node

def TriangleAttentionStartingNode($\{\mathbf{z}_{ij}\}, c = 32, N_{\text{head}} = 4$) : $\qquad \mathbf{z}_{ij} \in c_z$

# Input projections

$\mathbf{z}_{ij} \leftarrow \text{LayerNorm}(\mathbf{z}_{ij})$

$\mathbf{q}_{ij}^h, \mathbf{k}_{ij}^h, \mathbf{v}_{ij}^h = \text{LinearNoBias}(\mathbf{z}_{ij})$ $\qquad \mathbf{q}_{ij}^h, \mathbf{k}_{ij}^h, \mathbf{v}_{ij}^h \in \mathbb{R}^c,\ h \in \{1, \ldots, N_{\text{head}}\}$

$b_{ij}^h = \text{LinearNoBias}(\mathbf{z}_{ij})$

$\mathbf{g}_{ij}^h = \text{sigmoid}\left(\text{LinearNoBias}(\mathbf{z}_{ij})\right)$ $\qquad \mathbf{g}_{ij}^h \in \mathbb{R}^c$

# Attention

$a_{ijk}^h = \text{softmax}_k \left(\frac{1}{\sqrt{c}} \mathbf{q}_{ij}^{h\top} \mathbf{k}_{ik}^h + b_{jk}^h \right)$

$\mathbf{o}_{ij}^h = \mathbf{g}_{ij}^h \odot \sum_k a_{ijk}^h \mathbf{v}_{ik}^h$

# Output projection

$\tilde{\mathbf{z}}_{ij} = \text{LinearNoBias}\left(\text{concat}_h(\mathbf{o}_{ij}^h)\right)$ $\qquad \tilde{\mathbf{z}}_{ij} \in \mathbb{R}^{c_z}$

return $\{\tilde{\mathbf{z}}_{ij}\}$

Algorithm 15 Triangular gated self-attention around ending node (differences to Algorithm 14 highlighted)

def TriangleAttentionEndingNode($\{\mathbf{z}_{ij}\}, c = 32, N_{\text{head}} = 4$) : $\qquad \mathbf{z}_{ij} \in c_z$

# Input projections

$\mathbf{z}_{ij} \leftarrow \text{LayerNorm}(\mathbf{z}_{ij})$

$\mathbf{q}_{ij}^h, \mathbf{k}_{ij}^h, \mathbf{v}_{ij}^h = \text{LinearNoBias}(\mathbf{z}_{ij})$ $\qquad \mathbf{q}_{ij}^h, \mathbf{k}_{ij}^h, \mathbf{v}_{ij}^h \in \mathbb{R}^c,\ h \in \{1, \ldots, N_{\text{head}}\}$

$b_{ij}^h = \text{LinearNoBias}(\mathbf{z}_{ij})$

$\mathbf{g}_{ij}^h = \text{sigmoid}\left(\text{LinearNoBias}(\mathbf{z}_{ij})\right)$ $\qquad \mathbf{g}_{ij}^h \in \mathbb{R}^c$

# Attention

$a_{ijk}^h = \text{softmax}_k \left(\frac{1}{\sqrt{c}} \mathbf{q}_{ij}^{h\top} \mathbf{k}_{kj}^h + b_{ki}^h \right)$

$\mathbf{o}_{ij}^h = \mathbf{g}_{ij}^h \odot \sum_k a_{ijk}^h \mathbf{v}_{kj}^h$

# Output projection

$\tilde{\mathbf{z}}_{ij} = \text{LinearNoBias}\left(\text{concat}_h\left(\mathbf{o}_{ij}^h\right)\right)$ $\qquad \tilde{\mathbf{z}}_{ij} \in \mathbb{R}^{c_z}$

return $\{\tilde{\mathbf{z}}_{ij}\}$

3.5 Template embedding

The template embedding (Algorithm 16) combines all raw template features to a pair representation, and processes it together with the given pair representation $\mathbf{z}_{ij}$ (produced in the previous recyling iteration). This allows the network to attend to specific regions in the template based on its current belief about the structure.

The template_backbone_frame_mask, template_distogram, template_restype, template_pseudo_beta_mask and template_unit_vector features from Table 5 are concatenated into a 2D feature. In the main recycling loop (Algorithm 1) the pairwise embeddings $\mathbf{z}_{ij}$ are passed into the template embedder (Algorithm 16). Each template is processed independently with a PairformerStack, and the resulting activations are averaged to obtain a pairwise embedding of all templates.

Algorithm 16 Template embedder

def TemplateEmbedder$(\{\mathbf{f}^*\}, \{\mathbf{z}_{ij}\}, N_\text{block} = 2, c = 64)$:

$b_{ij}^\text{template\_backbone\_frame\_mask} = \mathrm{f}_{ti}^\text{template\_backbone\_frame\_mask} \cdot \mathrm{f}_{tj}^\text{template\_backbone\_frame\_mask}$

$b_{ij}^\text{template\_pseudo\_beta\_mask} = \mathrm{f}_{ti}^\text{template\_pseudo\_beta\_mask} \cdot \mathrm{f}_{tj}^\text{template\_pseudo\_beta\_mask}$

$\mathbf{a}_{tij} = \mathrm{concat}(\mathbf{f}_{tij}^\text{template\_distogram}, b_{ij}^\text{template\_backbone\_frame\_mask}, \mathbf{f}_{tij}^\text{template\_unit\_vector}, b_{ij}^\text{template\_pseudo\_beta\_mask})$

$\mathbf{a}_{tij} \leftarrow \mathbf{a}_{tij} \odot (\mathrm{f}_i^\text{asym\_id} == \mathrm{f}_j^\text{asym\_id})$

$\mathbf{a}_{tij} \leftarrow \mathrm{concat}(\mathbf{a}_{tij}, \mathbf{f}_{ti}^\text{template\_restype}, \mathbf{f}_{tj}^\text{template\_restype})$

$\mathbf{u}_{ij} = \mathbf{0}$ $\qquad \mathbf{u}_{ij} \in \mathbb{R}^c$

for all $t \in [1, \ldots, N_\text{templates}]$ **do**

  $\mathbf{v}_{ij} = \mathrm{LinearNoBias}(\mathrm{LayerNorm}(\mathbf{z}_{ij})) + \mathrm{LinearNoBias}(\mathbf{a}_{tij})$ $\qquad \mathbf{v}_{ij} \in \mathbb{R}^c$

  $\{\mathbf{v}_{ij}\} \mathrel{+}= \mathrm{PairformerStack}(\{\mathbf{v}_{ij}\}, N_\text{block})$

  $\mathbf{u}_{ij} \mathrel{+}= \mathrm{LayerNorm}(\mathbf{v}_{ij})$

end for

$\mathbf{u}_{ij} \leftarrow \frac{\mathbf{u}_{ij}}{N_\text{templates}}$

$\mathbf{u}_{ij} \leftarrow \mathrm{LinearNoBias}(\mathrm{ReLU}(\mathbf{u}_{ij}))$ $\qquad \mathbf{u}_{ij} \in \mathbb{R}^c$

return $\{\mathbf{u}_{ij}\}$

3.6 Pairformer stack

The Pairformer Stack (Algorithm 17, **Main Article Fig. 2a**) fulfills a similar role to the Evoformer stack in AlphaFold 2, the Pairformer stack uses just a single representation $\mathbf{s}_i$, rather than a representation for a subset of the MSA. Here the single representation plays a role similar to the privileged first row in the Evoformer in AlphaFold 2.

As a consequence of this change there is no column-wise attention in the stack. The single attention with pair bias is the same as the row-wise attention used in AlphaFold 2, but only applied to a single sequence, which corresponds to the single represenation $\mathbf{s}_i$.

Furthermore unlike in AlphaFold 2 the single representation does not influence the pair representation, the pair representation is used to control information flow in the single representation by biasing the Attention logits.

All Transition blocks use SwiGLU.

Algorithm 17 Pairformer stack

def $\text{PairformerStack}(\{\mathbf{s}_i\}, \{\mathbf{z}_{ij}\}, N_\text{block} = 48):$

for all $l \in [1, \ldots, N_\text{block}]$ **do**

# Pair stack

$\{\mathbf{z}_{ij}\} \mathrel{+}= \text{DropoutRowwise}_{0.25}(\text{TriangleMultiplicationOutgoing}(\{\mathbf{z}_{ij}\}))$

$\{\mathbf{z}_{ij}\} \mathrel{+}= \text{DropoutRowwise}_{0.25}(\text{TriangleMultiplicationIncoming}(\{\mathbf{z}_{ij}\}))$

$\{\mathbf{z}_{ij}\} \mathrel{+}= \text{DropoutRowwise}_{0.25}(\text{TriangleAttentionStartingNode}(\{\mathbf{z}_{ij}\}))$

$\{\mathbf{z}_{ij}\} \mathrel{+}= \text{DropoutColumnwise}_{0.25}(\text{TriangleAttentionEndingNode}(\{\mathbf{z}_{ij}\}))$

$\{\mathbf{z}_{ij}\} \mathrel{+}= \text{Transition}(\{\mathbf{z}_{ij}\})$

$\{\mathbf{s}_i\} \mathrel{+}= \text{AttentionPairBias}(\{\mathbf{s}_i\}, \emptyset, \{\mathbf{z}_{ij}\}, \beta_{ij} = 0, N_\text{head} = 16)$

$\{\mathbf{s}_i\} \mathrel{+}= \text{Transition}(\{\mathbf{s}_i\})$

end for

return $\{\mathbf{s}_i\}, \{\mathbf{z}_{ij}\}$

3.7 Diffusion Module

In AlphaFold 2 the final structure was realised using a Structure Module using invariant point attention. For AlphaFold 3 we replaced it with a relatively standard non-equivariant point-cloud diffusion model over all atoms (Algorithm 18 and **Main Article Fig. 2b**). During training, we train a denoiser to remove Gaussian noise from the positions of all heavy atoms conditioned on the features from the main trunk. The denoiser is based on a modern transformer, but with several modifications to make it more amenable to the task. The main changes are:

- We incorporate conditioning from the trunk in several ways: we initialise the activations from the single embedding, use a variant of Adaptive Layernorm [27] for the single conditioning and logit biasing for the pair conditioning.
- We use standard modern transformer tricks (e.g. SwiGLU [28]) and methods used in AlphaFold 2 (gating).
- We use a two-level architecture, working first on atoms, then tokens, then atoms again.

Notably, the transformer only uses a single linear layer to embed all atom positions and and a single linear layer to project the updates at the end, there are no geometric biases involved (e.g. locality or SE(3) invariance). This is in contrast to contemporary trends of using stronger domain specific inductive biases.

The details of the architecture are outlined in Algorithm 20.

Algorithm 18 Sample Diffusion

def SampleDiffusion($\{\mathbf{f}^*\}, \{\mathbf{s}_i^{\text{inputs}}\}, \{\mathbf{s}_i^{\text{trunk}}\}, \{\mathbf{z}_{ij}^{\text{trunk}}\}$, Noise Schedule $[c_0, c_1, \ldots, c_T]$,
$\gamma_0 = 0.8, \ \gamma_{\text{min}} = 1.0$, noise scale $\lambda = 1.003$, step scale $\eta = 1.5$):

1: $\vec{\mathbf{x}}_l \sim c_0 \cdot \mathcal{N}(\vec{\mathbf{0}}, \mathbf{I}_3)$ $\vec{\mathbf{x}}_l \in \mathbb{R}^3$
2: **for all** $c_\tau \in [c_1, \ldots, c_T]$ **do**
3: $\{\vec{\mathbf{x}}_l\} \leftarrow \text{CentreRandomAugmentation}(\{\vec{\mathbf{x}}_l\})$
4: $\gamma = \gamma_0$ if $c_\tau > \gamma_{\text{min}}$ else 0
5: $\hat{t} = c_{\tau-1}(\gamma + 1)$
6: $\vec{\xi}_l = \lambda \sqrt{\hat{t}^2 - c_{\tau-1}^2} \cdot \mathcal{N}(\vec{\mathbf{0}}, \mathbf{I}_3)$ $\vec{\xi}_l \in \mathbb{R}^3$
7: $\vec{\mathbf{x}}_l^{\text{noisy}} = \vec{\mathbf{x}}_l + \vec{\xi}_l$
8: $\{\vec{\mathbf{x}}_l^{\text{denoised}}\} = \text{DiffusionModule}(\{\vec{\mathbf{x}}_l^{\text{noisy}}\}, \hat{t}, \{\mathbf{f}^*\}, \{\mathbf{s}_i^{\text{inputs}}\}, \{\mathbf{s}_i^{\text{trunk}}\}, \{\mathbf{z}_{ij}^{\text{trunk}}\})$
9: $\vec{\delta}_l = (\vec{\mathbf{x}}_l - \vec{\mathbf{x}}_l^{\text{denoised}})/\hat{t}$
10: $dt = c_\tau - \hat{t}$
11: $\vec{\mathbf{x}}_l \leftarrow \vec{\mathbf{x}}_l^{\text{noisy}} + \eta \cdot dt \cdot \vec{\delta}_l$
12: **end for**
13: **return** $\{\vec{\mathbf{x}}_l\}$

Algorithm 19 CentreRandomAugmentation

def CentreRandomAugmentation($\{\vec{\mathbf{x}}_l\}, s_{\text{trans}} = 1\,\text{Å}$) :

1: $\vec{\mathbf{x}}_l \leftarrow \vec{\mathbf{x}}_l - \operatorname{mean}_l \vec{\mathbf{x}}_l$
2: $R = \text{UniformRandomRotation}()$
3: $\vec{\mathbf{t}} \sim s_{\text{trans}} \cdot \mathcal{N}(\vec{\mathbf{0}}, \mathbf{I}_3)$
4: $\vec{\mathbf{x}}_l \leftarrow R \cdot \vec{\mathbf{x}}_l + \vec{\mathbf{t}}$
5: **return** $\{\vec{\mathbf{x}}_l\}$

Algorithm 20 Diffusion Module

def $\text{DiffusionModule}(\{\vec{\mathbf{x}}_l^{\text{noisy}}\}, \hat{t}, \{\mathbf{f}^*\}, \{\mathbf{s}_i^{\text{inputs}}\}, \{\mathbf{s}_i^{\text{trunk}}\}, \{\mathbf{z}_{ij}^{\text{trunk}}\}, \sigma_{\text{data}} = 16, c_{\text{atom}} = 128, c_{\text{atompair}} = 16, c_{\text{token}} = 768):$

# Conditioning

$\{\mathbf{s}_i\}, \{\mathbf{z}_{ij}\} = \text{DiffusionConditioning}(\hat{t}, \{\mathbf{f}^*\}, \{\mathbf{s}_i^{\text{inputs}}\}, \{\mathbf{s}_i^{\text{trunk}}\}, \{\mathbf{z}_{ij}^{\text{trunk}}\}, \sigma_{\text{data}})$

# Scale positions to dimensionless vectors with approximately unit variance.

$\mathbf{r}_l^{\text{noisy}} = \vec{\mathbf{x}}_l^{\text{noisy}} / \sqrt{\hat{t}^2 + \sigma_{\text{data}}^2}$ $\qquad \mathbf{r}_l^{\text{noisy}} \in \mathbb{R}^3$

# Sequence-local Atom Attention and aggregation to coarse-grained tokens

$\{\mathbf{a}_i\}, \{\mathbf{q}_l^{\text{skip}}\}, \{\mathbf{c}_l^{\text{skip}}\}, \{\mathbf{p}_{lm}^{\text{skip}}\} = \text{AtomAttentionEncoder}(\{\mathbf{f}^*\}, \{\mathbf{r}_l^{\text{noisy}}\}, \{\mathbf{s}_i^{\text{trunk}}\}, \{\mathbf{z}_{ij}\}, c_{\text{atom}}, c_{\text{atompair}}, c_{\text{token}})$ $\qquad \mathbf{a}_i \in \mathbb{R}^{c_{\text{token}}}$

# Full self-attention on token level.

$\mathbf{a}_i \mathrel{+}= \text{LinearNoBias}(\text{LayerNorm}(\mathbf{s}_i))$

$\{\mathbf{a}_i\} \leftarrow \text{DiffusionTransformer}(\{\mathbf{a}_i\}, \{\mathbf{s}_i\}, \{\mathbf{z}_{ij}\}, \beta_{ij} = 0, N_{\text{block}} = 24, N_{\text{head}} = 16)$

$\mathbf{a}_i \leftarrow \text{LayerNorm}(\mathbf{a}_i)$

# Broadcast token activations to atoms and run Sequence-local Atom Attention

$\{\mathbf{r}_l^{\text{update}}\} = \text{AtomAttentionDecoder}(\{\mathbf{a}_i\}, \{\mathbf{q}_l^{\text{skip}}\}, \{\mathbf{c}_l^{\text{skip}}\}, \{\mathbf{p}_{lm}^{\text{skip}}\})$

# Rescale updates to positions and combine with input positions

$\vec{\mathbf{x}}_l^{\text{out}} = \sigma_{\text{data}}^2 / (\sigma_{\text{data}}^2 + \hat{t}^2) \cdot \vec{\mathbf{x}}_l^{\text{noisy}} + \sigma_{\text{data}} \cdot \hat{t} / \sqrt{\sigma_{\text{data}}^2 + \hat{t}^2} \cdot \mathbf{r}_l^{\text{update}}$

return $\{\vec{\mathbf{x}}_l^{\text{out}}\}$

Algorithm 21 Diffusion Conditioning

def $\text{DiffusionConditioning}(\hat{t}, \{\mathbf{f}^*\}, \{\mathbf{s}_i^{\text{inputs}}\}, \{\mathbf{s}_i^{\text{trunk}}\}, \{\mathbf{z}_{ij}^{\text{trunk}}\}, \sigma_{\text{data}}, c_{\text{z}} = 128, c_{\text{s}} = 384):$

# Pair conditioning

$\mathbf{z}_{ij} = \text{concat}([\mathbf{z}_{ij}^{\text{trunk}}, \text{RelativePositionEncoding}(\{\mathbf{f}^*\})])$

$\mathbf{z}_{ij} \leftarrow \text{LinearNoBias}(\text{LayerNorm}(\mathbf{z}_{ij}))$ $\qquad \mathbf{z}_{ij} \in \mathbb{R}^{c_{\text{z}}}$

for all $b \in [1, 2]$ **do**

$\quad \mathbf{z}_{ij} \mathrel{+}= \text{Transition}(\mathbf{z}_{ij},\ n = 2)$

end for

# Single conditioning

$\mathbf{s}_i = \text{concat}([\mathbf{s}_i^{\text{trunk}}, \mathbf{s}_i^{\text{inputs}}])$

$\mathbf{s}_i \leftarrow \text{LinearNoBias}(\text{LayerNorm}(\mathbf{s}_i))$ $\qquad \mathbf{s}_i \in \mathbb{R}^{c_{\text{s}}}$

$\mathbf{n} = \text{FourierEmbedding}(\frac{1}{4} \log(\hat{t}/\sigma_{\text{data}}), 256)$

$\mathbf{s}_i \mathrel{+}= \text{LinearNoBias}(\text{LayerNorm}(\mathbf{n}))$

for all $b \in [1, 2]$ **do**

$\quad \mathbf{s}_i \mathrel{+}= \text{Transition}(\mathbf{s}_i,\ n = 2)$

end for

return $\{\mathbf{s}_i\}, \{\mathbf{z}_{ij}\}$

Algorithm 22 Fourier Embedding

def FourierEmbedding($\hat{t}, c$)

# Randomly generate weight/bias once before training

$\mathbf{w}, \mathbf{b} \sim \mathcal{N}(\vec{\mathbf{0}}, \mathbf{I}_c)$ $\qquad \mathbf{w}, \mathbf{b} \in \mathbb{R}^c$

# Compute embeddings

return $\cos(2\pi(\hat{t}\mathbf{w} + \mathbf{b}))$

Algorithm 23 Diffusion Transformer

def DiffusionTransformer($\{\mathbf{a}_i\}, \{\mathbf{s}_i\}, \{\mathbf{z}_{ij}\}, \{\beta_{ij}\}, N_{\text{block}}, N_{\text{head}}$):

for all $n \in [1, \ldots, N_{\text{block}}]$ **do**
  $\{\mathbf{b}_i\} = \text{AttentionPairBias}(\{\mathbf{a}_i\}, \{\mathbf{s}_i\}, \{\mathbf{z}_{ij}\}, \{\beta_{ij}\}, N_{\text{head}})$
  $\mathbf{a}_i \leftarrow \mathbf{b}_i + \text{ConditionedTransitionBlock}(\mathbf{a}_i, \mathbf{s}_i)$
end for
return $\{\mathbf{a}_i\}$

Algorithm 24 DiffusionAttention with pair bias and mask

def AttentionPairBias($\{\mathbf{a}_i\}, \{\mathbf{s}_i\}, \{\mathbf{z}_{ij}\}, \{\beta_{ij}\}, N_{\text{head}}$) : $\qquad \mathbf{a}_i \in \mathbb{R}^{c_\text{a}}, c = c_\text{a}/N_{\text{head}}$

# Input projections

if $\{\mathbf{s}_i\} \neq \emptyset$ **then**
  $\mathbf{a}_i \leftarrow \text{AdaLN}(\mathbf{a}_i, \mathbf{s}_i)$
else
  $\mathbf{a}_i \leftarrow \text{LayerNorm}(\mathbf{a}_i)$
end if
$\mathbf{q}_i^h = \text{Linear}(\mathbf{a}_i)$ $\qquad \mathbf{q}_i^h \in \mathbb{R}^c,\ h \in \{1, \ldots, N_{\text{head}}\}$
$\mathbf{k}_i^h, \mathbf{v}_i^h = \text{LinearNoBias}(\mathbf{a}_i)$ $\qquad \mathbf{k}_i^h, \mathbf{v}_i^h \in \mathbb{R}^c,\ h \in \{1, \ldots, N_{\text{head}}\}$
$b_{ij}^h \leftarrow \text{LinearNoBias}(\text{LayerNorm}(\mathbf{z}_{ij})) + \beta_{ij}$
$\mathbf{g}_i^h \leftarrow \text{sigmoid}\left(\text{LinearNoBias}(\mathbf{a}_i)\right)$ $\qquad \mathbf{g}_i^h \in \mathbb{R}^c$

# Attention

$A_{ij}^h \leftarrow \text{softmax}_j\left(\frac{1}{\sqrt{c}}\,\mathbf{q}_i^{h\top}\mathbf{k}_j^h + b_{ij}^h\right)$
$\mathbf{a}_i \leftarrow \text{LinearNoBias}\left(\text{concat}_h\left(\mathbf{g}_i^h \odot \sum_j A_{ij}^h \mathbf{v}_j^h\right)\right)$ $\qquad \mathbf{a}_i \in \mathbb{R}^{c_\text{a}}$

# Output projection (from adaLN-Zero [27])

if $\{\mathbf{s}_i\} \neq \emptyset$ **then**
  $\mathbf{a}_i \leftarrow \text{sigmoid}(\text{Linear}(\mathbf{s}_i, \text{biasinit=-2.0})) \odot \mathbf{a}_i$
end if
return $\{\mathbf{a}_i\}$

Algorithm 25 Conditioned Transition Block

# SwiGLU transition block with adaptive layernorm

def $\mathrm{ConditionedTransitionBlock}(\mathbf{a}, \mathbf{s}, n = 2)$: $\mathbf{a} \in \mathbb{R}^{c}$

$\mathbf{a} \leftarrow \mathrm{AdaLN}(\mathbf{a}, \mathbf{s})$

$\mathbf{b} \leftarrow \mathrm{swish}(\mathrm{LinearNoBias}(\mathbf{a})) \odot \mathrm{LinearNoBias}(\mathbf{a})$ $\mathbf{b} \in \mathbb{R}^{n \cdot c}$

# Output projection (from adaLN-Zero [27])

$\mathbf{a} \leftarrow \mathrm{sigmoid}(\mathrm{Linear}(\mathbf{s}, \text{biasinit=-2.0})) \odot \mathrm{LinearNoBias}(\mathbf{b})$ $\mathbf{a} \in \mathbb{R}^{c}$

return $\mathbf{a}$

Algorithm 26 Adaptive LayerNorm

def $\mathrm{AdaLN}(\mathbf{a}, \mathbf{s})$:

$\mathbf{a} \leftarrow \mathrm{LayerNorm}(\mathbf{a}, \text{ scale=False, offset=False})$

$\mathbf{s} \leftarrow \mathrm{LayerNorm}(\mathbf{s}, \text{ offset=False})$

$\mathbf{a} \leftarrow \mathrm{sigmoid}(\mathrm{Linear}(\mathbf{s})) \odot \mathbf{a} + \mathrm{LinearNoBias}(\mathbf{s})$

return $\mathbf{a}$

3.7.1 Diffusion Training

Our diffusion training methodology largely follows [29]. With some notable differences, mostly to the loss.

To improve training efficiency we train the Diffusion Module with a larger batch size than the trunk (see **Main Article Fig. 2c**). To realise this, we run the trunk once and then create 48 versions of the input structure by randomly rotating and translating according to Algorithm 19 and adding independent noise to each structure. We then train the Diffusion Module over all of them in parallel. This is efficient since the Diffusion Module is much cheaper than the model trunk.

We apply a weighted aligned MSE loss to the denoised structure output from the Diffusion Module. We first perform a rigid alignment of the ground truth $\vec{\mathbf{x}}_l^{\text{GT}}$ on to the denoised structure $\vec{\mathbf{x}}_l$ as

$$\{\vec{\mathbf{x}}_l^{\text{GT-aligned}}\} = \text{weighted\_rigid\_align}(\{\vec{\mathbf{x}}_l^{\text{GT}}\}, \{\vec{\mathbf{x}}_l\}\}, \{w_l\}) \tag{2}$$

with weights w_l provided in Equation 4. We then compute a weighted MSE

$$\mathcal{L}_{\text{MSE}} = \frac{1}{3} \operatorname*{mean}_l \left(w_l ||\vec{\mathbf{x}}_l - \vec{\mathbf{x}}_l^{\text{GT-aligned}}||^2 \right), \tag{3}$$

with upweighting of nucleotide and ligand atoms as

$$w_l = 1 + \mathrm{f}_l^{\text{is\_dna}} \alpha^{\text{dna}} + \mathrm{f}_l^{\text{is\_rna}} \alpha^{\text{rna}} + \mathrm{f}_l^{\text{is\_ligand}} \alpha^{\text{ligand}} \tag{4}$$

and hyperparameters $\alpha^{\text{dna}} = \alpha^{\text{rna}} = 5$, and $\alpha^{\text{ligand}} = 10$.

To ensure that the bonds for bonded ligands (including bonded glycans) have the correct length, we introduce an auxiliary loss during fine tuning as

$$\mathcal{L}_{\text{bond}} = \operatorname*{mean}_{(l,m) \in \mathcal{B}} \left(\left\| \vec{\mathbf{x}}_l - \vec{\mathbf{x}}_m \right\| - \left\| \vec{\mathbf{x}}_l^{\text{GT}} - \vec{\mathbf{x}}_m^{\text{GT}} \right\| \right)^2, \tag{5}$$

where $\mathcal{B}$ is the set of tuples (start atom index, end atom index) defining the bond between the bonded ligand and its parent chain.

We also apply an auxiliary structure-based loss based on smooth LDDT, as described in Algorithm 27. The final loss from the Diffusion Module is then:

$$\mathcal{L}_{\text{diffusion}} = (\hat{t}^2 + \sigma_{\text{data}}^2)/(\hat{t} + \sigma_{\text{data}})^2 \cdot (\mathcal{L}_{\text{MSE}} + \alpha_{bond} \cdot \mathcal{L}_{\text{bond}}) + \mathcal{L}_{\text{smooth\_lddt}} \tag{6}$$

Where $\hat{t}$ is the sampled noise level, σ_{data} is a constant determined by the variance of the data (set to 16) and α_{bond} is 0 for regular training, and 1 for both fine tuning stages. Prior to computing these losses, we apply an optimal ground truth chain assignment as described in subsection 4.2.

During training the noise level is sampled from $\sigma_{\text{data}} \cdot \exp(-1.2 + 1.5 \cdot \mathcal{N}(0, 1))$, during inference the noise schedule is defined as

$$\hat{t} = \sigma_{\text{data}} \cdot (s_{\text{max}}^{1/p} + t \cdot (s_{\text{min}}^{1/p} - s_{\text{max}}^{1/p}))^p \tag{7}$$

where $s_{\text{max}} = 160$, $s_{\text{min}} = 4 \cdot 10^{-4}$, $p = 7$ and t is distributed uniformly between [0, 1] with a step size of $\frac{1}{200}$.

Algorithm 27 Smooth LDDT Loss

def $\text{SmoothLDDTLoss}(\{\vec{\mathbf{x}}_l\}, \{\vec{\mathbf{x}}_l^{\text{GT}}\}, \{\text{f}_l^{\text{is\_dna}}\}, \{\text{f}_l^{\text{is\_rna}}\})$:

# Compute distances between all pairs of atoms

$\delta x_{lm} \leftarrow ||\vec{\mathbf{x}}_l - \vec{\mathbf{x}}_m||$

$\delta x_{lm}^{\text{GT}} \leftarrow ||\vec{\mathbf{x}}_l^{\text{GT}} - \vec{\mathbf{x}}_m^{\text{GT}}||$

# Compute distance difference for all pairs of atoms

$\delta_{lm} \leftarrow \text{abs}(\delta x_{lm}^{\text{GT}} - \delta x_{lm})$

$\epsilon_{lm} \leftarrow \frac{1}{4}\left[\text{sigmoid}(\frac{1}{2} - \delta_{lm}) + \text{sigmoid}(1 - \delta_{lm})) + \text{sigmoid}(2 - \delta_{lm}) + \text{sigmoid}(4 - \delta_{lm})\right]$

# Restrict to bespoke inclusion radius

$f_l^{\text{is\_nucleotide}} \leftarrow \text{f}_l^{\text{is\_dna}} + \text{f}_l^{\text{is\_rna}}$

$c_{lm} \leftarrow (\delta x_{lm}^{\text{GT}} < 30\,\text{Å}) f_l^{\text{is\_nucleotide}} +$
$(\delta x_{lm}^{\text{GT}} < 15\,\text{Å})(1 - f_l^{\text{is\_nucleotide}})$

# Compute mean, avoiding self term

$\text{lddt} = \underset{l \neq m}{\text{mean}}(c_{lm}\epsilon_{lm}) / \underset{l \neq m}{\text{mean}}(c_{lm})$

return $1 - \text{lddt}$

Algorithm 28 Weighted Rigid Align

def weighted_rigid_align($\{\vec{\mathbf{x}}_l\}, \{\vec{\mathbf{x}}_l^{\text{GT}}\}, \{w_l\}$) :

# Mean-centre positions

$\vec{\mu} \leftarrow \operatorname{mean}_l(w_l \vec{\mathbf{x}}_l) / \operatorname{mean}_l(w_l)$

$\vec{\mu}^{\text{GT}} \leftarrow \operatorname{mean}_l(w_l \vec{\mathbf{x}}_l^{\text{GT}}) / \operatorname{mean}_l(w_l)$

$\vec{\mathbf{x}}_l \leftarrow \vec{\mathbf{x}}_l - \vec{\mu}$

$\vec{\mathbf{x}}_l^{\text{GT}} \leftarrow \vec{\mathbf{x}}_l^{\text{GT}} - \vec{\mu}^{\text{GT}}$

# Find optimal rotation from singular value decomposition

$U, V \leftarrow \text{svd}(\sum_l w_l \vec{\mathbf{x}}_l^{\text{GT}} \otimes \vec{\mathbf{x}}_l)$

$R \leftarrow UV$

# Remove reflection

if $\det(R) < 0$ **then**

$\quad F \leftarrow \begin{pmatrix} 1 & 0 & 0 \\ 0 & 1 & 0 \\ 0 & 0 & -1 \end{pmatrix}$

$\quad R \leftarrow UFV$

end if

# Apply alignment

$\vec{\mathbf{x}}_l^{\text{align}} = R\vec{\mathbf{x}}_l + \vec{\mu}$

return stop_gradient($\vec{\mathbf{x}}_l^{\text{align}}$)

4 Auxiliary heads

4.1 Mini diffusion rollout

Several of the heads require predicted coordinates, therefore at training time we do a short rollout of the Diffusion Module from pure noise with 20 steps (**Main Article Fig. 2c**). No gradients are applied to this mini-rollout.

4.2 Chain permutation and symmetry resolution

The assignment of names (chain-ids, like ‘A’, ‘B’, ‘C’, ...) to chains with identical sequences in a physical structure is arbitrary, and every permutation of these names is equally correct. The same applies to multiple ligands of the same type. It is very likely that AlphaFold will use a different permutation than the authors who deposited the ground truth in the PDB. To compare a predicted structure to the ground truth in the confidence head (subsubsection 4.3.1) or to create the noised version of the ground truth for diffusion training (subsection 3.7), the ground truth chains must be renamed such that they match the predicted structure. We use the method described in [18] section 7.3 “Multi-Chain Permutation Alignment” to find a good assignment of the predicted chains to the ground truth chains. The predicted structure used for this alignment is the output of the mini-diffusion rollout (subsection 4.2). The same method is applied to ligands as well, with the extension that ligands covalently bonded to polymer chains will be permuted in sync with the corresponding chains. This is achieved by grouping all covalently bonded components together and assigning them the same entity id.

Finally, we resolve atom naming ambiguities within each chemical component (ligand/residue): we generate permutations (up to 1000) using RDKit and permute the ground truth such that it has minimal RMSD to the prediction.

4.3 Model confidence prediction

The model is trained to predict three confidence metrics, a per-atom confidence, predicted local distance difference (pLDDT), a pairwise atom-atom aligned error (PAE), and a pairwise atom-atom distance confidence, predicted distance error (PDE). It is also trained to predict whether an atom is experimentally resolved. The confidence losses are only

applied for the PDB training set (i.e. it is not applied for any of the distillation sets), with a further filter that the resolution of the ground truth structure is between 0.1 and 4. The details of the confidence head and losses are outlined below.

4.3.1 Predicted local distance difference test (pLDDT)

The per atom confidence is trained to predict a version of LDDT that takes into account distances from all atoms to polymer residues (e.g. for a ligand atom the confidence only takes into account interactions between the ligand atom and proteins/nucleic acids, no intra-ligand terms are included), it is defined for atom l as:

$$\text{lddt}_l = \sum_{m \in R} \frac{1}{4} \sum_{c \in \{0.5,1,2,4\}} d_{lm} < c \tag{8}$$

Where d_{lm} is the distance between atom l and atom m in the mini-rollout prediction (subsection 4.1), l encompasses all atoms and the set of atoms $m \in R$ is defined as:

- Atoms such that the distance in the ground truth between atom l and atom m is less than 15 Å if m is a protein atom or less than 30 Å if m is a nucleic acid atom.
- Only atoms in polymer chains.
- One atom per token - C^α for standard protein residues and $\text{C1}'$ for standard nucleic acid residues.

The single embedding ($\mathbf{s}_i$) in the confidence head (Algorithm 31) is linearly projected into $[N_{\text{max\_atoms\_per\_token}}, 50]$ values, where $N_{\text{max\_atoms\_per\_token}}$ is the maximum number of atoms per token. This gives per atom confidences with 50 bins between 0 and 1 and probabilities p_l^b obtained via a softmax. The loss is then defined as:

$$\mathcal{L}_{\text{plddt}} = -\frac{1}{N_{\text{atom}}} \sum_l \sum_{b=1}^{50} \text{lddt}_l^b \log p_l^b \tag{9}$$

A single value of pLDDT per-atom is obtained by taking the expectation across the 50 binned probabilities. Here lddt_l^b denotes a binned version of lddt_l, i.e. lddt_l^b is 1 if lddt_l falls within bin b and 0 otherwise.

4.3.2 Predicted aligned error (PAE)

It is useful to have a pairwise confidence between atoms, to determine confidence of interfaces or specific interactions between atoms. To this end we follow AlphaFold 2 and predict a pairwise alignment error (PAE), an estimate of the error of one token when aligned according to the frame of another.

We associate a reference frame to each token i using a set of three atoms which we denote as (a_i, b_i, c_i). Let $\Phi_i = (\vec{\mathbf{a}}_i, \vec{\mathbf{b}}_i, \vec{\mathbf{c}}_i)$ denote the coordinates of these frame atoms. An atom position $\vec{\mathbf{x}}$ is expressed in a basis defined by Φ_i according to Algorithm 29, which takes $\vec{\mathbf{b}}_i$ as its origin and applies to $\vec{\mathbf{x}}$ a rotation defined by Φ_i. To compute an aligned error e_{ij} between frame i and token j, Φ_i and Φ_i^{true} are built from the predicted and ground truth frame atom coordinates for token i, and representative token atom coordinates from the prediction $\vec{\mathbf{x}}_j$ and ground truth $\vec{\mathbf{x}}_j^{\text{true}}$ are expressed in the bases of Φ_i and Φ_i^{true}, respectively. The error e_{ij} is then defined as the Euclidean distance between these two alignments. See Algorithm 30 for pseudocode.

The atoms (a_i, b_i, c_i) used to construct token i's frame depend on the chain type of i: Protein tokens use their residue's backbone $(\text{N}, \text{C}^\alpha, \text{C})$, while DNA and RNA tokens use $(\text{C1}', \text{C3}', \text{C4}')$ atoms of their residue. All other tokens (small molecules, glycans, ions) contain only one atom per token. The token atom is assigned to b_i, the closest atom to the token atom is a_i, and the second closest atom to the token atom is c_i. If this set of three atoms is close to colinear (less than 25 degree deviation), or if three atoms do not exist in the chain (e.g. a sodium ion), then the frame is marked as invalid. The PAE head does not train on invalid frames, nor do ranking and confidence metrics consider them.

When computing the alignment error we use the predicted coordinates taken from the mini-rollout structure.

Algorithm 29 Express coordinates in frame

def expressCoordinatesInFrame$(\vec{\mathbf{x}}, \mathbf{\Phi})$: $\quad \vec{\mathbf{x}} \in \mathbb{R}^3$

# Extract frame atoms

$(\vec{\mathbf{a}}, \vec{\mathbf{b}}, \vec{\mathbf{c}}) = \mathbf{\Phi}$ $\quad \vec{\mathbf{a}}, \vec{\mathbf{b}}, \vec{\mathbf{c}} \in \mathbb{R}^3$

$\vec{\mathbf{w}}_1 = (\vec{\mathbf{a}} - \vec{\mathbf{b}}) / \left\| \vec{\mathbf{a}} - \vec{\mathbf{b}} \right\|$

$\vec{\mathbf{w}}_2 = (\vec{\mathbf{c}} - \vec{\mathbf{b}}) / \left\| \vec{\mathbf{c}} - \vec{\mathbf{b}} \right\|$

# Build orthonormal basis

$\vec{\mathbf{e}}_1 = (\vec{\mathbf{w}}_1 + \vec{\mathbf{w}}_2) / \left\| \vec{\mathbf{w}}_1 + \vec{\mathbf{w}}_2 \right\|$

$\vec{\mathbf{e}}_2 = (\vec{\mathbf{w}}_2 - \vec{\mathbf{w}}_1) / \left\| \vec{\mathbf{w}}_2 - \vec{\mathbf{w}}_1 \right\|$

$\vec{\mathbf{e}}_3 = \vec{\mathbf{e}}_1 \times \vec{\mathbf{e}}_2$

# Project onto frame basis

$\vec{\mathbf{d}} = \vec{\mathbf{x}} - \vec{\mathbf{b}}$

$\vec{\mathbf{x}}^{\text{transformed}} = \text{concat}(\vec{\mathbf{d}} \cdot \vec{\mathbf{e}}_1, \vec{\mathbf{d}} \cdot \vec{\mathbf{e}}_2, \vec{\mathbf{d}} \cdot \vec{\mathbf{e}}_3)$

return $\vec{\mathbf{x}}^{\text{transformed}}$ $\quad \vec{\mathbf{x}}^{\text{transformed}} \in \mathbb{R}^3$

Algorithm 30 Compute alignment error

def computeAlignmentError$(\{\vec{\mathbf{x}}_i\}, \{\vec{\mathbf{x}}_i^{\text{true}}\}, \{\Phi_i\}, \{\Phi_i^{\text{true}}\}, \epsilon = 1e^{-8}\,\text{Å}^2)$:

$\hat{\vec{\mathbf{x}}}_{ij} = \text{expressCoordinatesInFrame}(\vec{\mathbf{x}}_j, \Phi_i)$

$\hat{\vec{\mathbf{x}}}_{ij}^{\text{true}} = \text{expressCoordinatesInFrame}(\vec{\mathbf{x}}_j^{\text{true}}, \Phi_i^{\text{true}})$

$e_{ij} = \sqrt{\|\hat{\vec{\mathbf{x}}}_{ij} - \hat{\vec{\mathbf{x}}}_{ij}^{\text{true}}\|^2 + \epsilon}$

return $\{e_{ij}\}$

To predict the alignment error, the pair embedding $(\mathbf{z}_{ij})$ in the confidence head (Algorithm 31) is linearly projected into 64 distance bins b, and probabilities p_{ij}^b are obtained via a softmax. There are 64 bins equally spaced from 0 Å to 32 Å in 0.5 Å increments. During training the final bin also captures larger errors. The prediction targets are the alignment errors e_{ij}. The loss is then defined as:

$$\mathcal{L}_{\text{pae}} = -\frac{1}{N_{\text{token}}^2} \sum_{i,j} \sum_{b=1}^{64} e_{ij}^b \log p_{ij}^b \tag{10}$$

Here e_{ij}^b denotes a binned version of e_{ij}, i.e. e_{ij}^b is 1 if e_{ij} falls within bin b and 0 otherwise.

A single value of PAE per token-pair is obtained by taking the expectation across the 64 binned probabilities:

$$\text{PAE}_{ij} = \sum_{b=1}^{64} \Delta_b \, p_{ij}^b \tag{11}$$

where Δ_b are the distance bin centers.

We also use the probabilities p_{ij}^b to compute pTM, an estimate of the TM-Score [30] as well as an ipTM, an interfacial variant that only considers (i, j) pairs from different chains. For full description of pTM and ipTM we refer the reader to Sec. 1.9.7 of [1] and Sec. 7.9 of [18], respectively. As previously, we find pTM and ipTM to be useful as confidence metrics (main text Figure 4) and for sample ranking (subsection 5.9).

4.3.3 Predicted distance error (PDE)

In addition to an alignment error, the model is also trained to predict the error in absolute distances between atoms.

The pair embedding $(\mathbf{z}_{ij})$ in the confidence head (Algorithm 31) is linearly projected into 64 distance bins b, and probabilities p_{ij}^b are obtained via a softmax. There are 64 bins equally spaced from 0 Å to 32 Å in 0.5 Å increments.

During training the final bin also captures larger errors. The prediction targets e_{ij} are defined as $e_{ij} = |d_{ij}^{\text{pred}} - d_{ij}^{gt}|$, where d_{ij}^{pred} is the distance between representative token atoms i and j in the mini-rollout prediction (subsection 4.1), and d_{ij}^{gt} is the corresponding distance in the ground truth. The loss is then defined as:

$$\mathcal{L}_{\text{pde}} = -\frac{1}{N_{\text{token}}^2} \sum_{i,j} \sum_{b=1}^{64} e_{ij}^b \log p_{ij}^b \tag{12}$$

Here e_{ij}^b denotes a binned version of e_{ij}, i.e. e_{ij}^b is 1 if e_{ij} falls within bin b and 0 otherwise.

A single value of PDE per token-pair is obtained by taking the expectation across the 64 binned probabilities.

$$\text{PDE}_{ij} = \sum_{b=1}^{64} \Delta_b \, p_{ij}^b \tag{13}$$

where Δ_b are the distance bin centers.

4.3.4 Experimentally resolved prediction

The single embedding ($\mathbf{s}_i$) in the confidence head is linearly projected into per-atom values (in the same way as subsubsection 4.3.1) with 2 bins. Then a softmax is used to predict whether the atom is resolved in the ground truth (y_l), and loss defined as:

$$\mathcal{L}_{\text{resolved}} = -\frac{1}{N_{\text{atom}}} \sum_{l} \sum_{b=1}^{2} y_l^b \log p_l^b \tag{14}$$

4.3.5 Confidence head architecture

The pLDDT, PAE, PDE and experimentally resolved outputs are projected from the same head, the architecture of this head is outlined in Algorithm 31. The pairwise distance between the representative atoms for each token are extracted, and combined with the single ($\{\mathbf{s}_i\}$) and pair ($\{\mathbf{z}_{ij}\}$) embeddings from the network trunk. $l_{\text{rep}}(i)$ provides the flat-atom index l of the representative atom for the given token i. During training, a stop gradient is applied to the single and pair embeddings and predicted structure.

Algorithm 31 Confidence head

def $\text{ConfidenceHead}(\{\mathbf{s}_i^{\text{inputs}}\}, \{\mathbf{s}_i\}, \{\mathbf{z}_{ij}\}, \{\vec{\mathbf{x}}_l^{\text{pred}}\}, N_{\text{block}} = 4)$:

$\mathbf{z}_{ij} \mathrel{+}= \text{LinearNoBias}(\mathbf{s}_i^{\text{inputs}}) + \text{LinearNoBias}(\mathbf{s}_j^{\text{inputs}})$

# Embed pair distances of representative atoms:

$d_{ij} = \left\| \vec{\mathbf{x}}_{l_{\text{rep}}(i)}^{\text{pred}} - \vec{\mathbf{x}}_{l_{\text{rep}}(j)}^{\text{pred}} \right\|$

$\mathbf{z}_{ij} \mathrel{+}= \text{LinearNoBias}(\text{one\_hot}(d_{ij}, \mathbf{v}_{\text{bins}} = [3\tfrac{3}{8}\text{ Å},\ 5\tfrac{1}{8}\text{ Å},\ \ldots, 21\tfrac{3}{8}\text{ Å}]))$

$\{\mathbf{s}_i\}, \{\mathbf{z}_{ij}\} \mathrel{+}= \text{PairformerStack}(\{\mathbf{s}_i\}, \{\mathbf{z}_{ij}\}, N_{\text{block}})$

$\mathbf{p}_{ij}^{\text{pae}} = \text{softmax}(\text{LinearNoBias}(\mathbf{z}_{ij}))$ $\qquad \mathbf{p}_{ij}^{\text{pae}} \in \mathbb{R}^{b_{\text{pae}}}$

$\mathbf{p}_{ij}^{\text{pde}} = \text{softmax}(\text{LinearNoBias}(\mathbf{z}_{ij} + \mathbf{z}_{ji}))$ $\qquad \mathbf{p}_{ij}^{\text{pde}} \in \mathbb{R}^{b_{\text{pde}}}$

$\mathbf{p}_l^{\text{plddt}} = \text{softmax}(\text{LinearNoBias}_{\text{token\_atom\_idx}(l)}(\mathbf{s}_{\text{i}(l)}))$ $\qquad \mathbf{p}_l^{\text{plddt}} \in \mathbb{R}^{b_{\text{plddt}}}$

$\mathbf{p}_l^{\text{resolved}} = \text{softmax}(\text{LinearNoBias}_{\text{token\_atom\_idx}(l)}(\mathbf{s}_{\text{i}(l)}))$ $\qquad \mathbf{p}_l^{\text{resolved}} \in \mathbb{R}^2$

return $\{\mathbf{p}_l^{\text{plddt}}\}, \{\mathbf{p}_{ij}^{\text{pae}}\}, \{\mathbf{p}_{ij}^{\text{pde}}\}, \{\mathbf{p}_l^{\text{resolved}}\}$

4.4 Distogram prediction

The model is also trained to predict binned distances between all pairs of tokens (a distogram). This head and loss are identical to AlphaFold 2 [1], where the pairwise token distances use the representative atom for each token: C^β for protein residues (C^α for glycine), C4 for purines and C2 for pyrimidines. All ligands already have a single atom per token.

5 Training and inference

5.1 Structure filters

For both training and inference, standard crystallization agents (Table 9) are removed from the structure. Bonds for structures with homomeric subcomplexes lacking the corresponding homomeric symmetry are also removed – e.g. if a certain bonded ligand only exists for some of the symmetric copies, but not for all, we remove the corresponding bond information from the input. In consequence the model has to learn to infer these bonds by itself.

5.2 Training stages

Training occurs in four stages with differences highlighted in Table 6. We train a randomly initialized model first with a sequence crop size of 384, and then fine tune from the stage one weights at a crop size of 640, followed by a second fine tuning stage with crop size 768. In the first stage of fine tuning, smooth lddt loss was turned off and the weight of the disordered protein PDB distillation set was reduced, but now with unmasked diffusion loss on non-protein chains in that distillation set. For the second stage of fine tuning, in addition, transcription factor distillation sets were turned on and the weight of the disordered protein PDB distillation set was increased back to its original level. Protein residues from the DNA motif distillation set with predicted probability of being experimentally resolved lower than 0.9 were treated as unresolved residues. In the third, final fine-tuning stage, we trained the PAE head while removing all structure-based losses (diffusion and distogram) from training and increased the maximum number of chains to 50. All other settings were kept as in the previous fine-tuning stage.

The model trained for PoseBusters was trained similarly to the primary model, but without PDB structures released after 2021-09-30, without RNA MSA or RNA distillation sets, and without the predicted experimentally resolved filter on the DNA motif distillation set during fine tuning.

Table 6 | Training stages

	**Initial training**	**Fine tuning 1**	**Fine tuning 2**	**Fine tuning 3**
Sequence crop size N_{token}	384	640	768	768
Parameters initialized from	Random	Initial training	Fine tuning 1	Fine tuning 2
Sampling weight for disorder PDB distillation	0.02	0.01	0.02	0.02
Train on transcription factor distillation sets	False	False	True	True
Masked diffusion loss for non-protein in disorder PDB distillation	True	False	False	False
Train structure and distogram	True	True	True	False
Train PAE head	False	False	False	True
Diffusion batch size	48	32	32	32
Training samples ($\cdot 10^6$)	$\approx$20	$\approx$1.5	$\approx$1.5	$\approx$1.8
Training times (days on 256 A100s)	$\approx$10	$\approx$3	$\approx$5	$\approx$2
Polymer-ligand bond loss weight	0	1	1	1
Max number of chains	20	20	20	50

5.3 Loss

The details of the losses from each module are given in the relevant sections, these are then combined into the final loss:

$$\mathcal{L}_{\text{loss}} = \alpha_{\text{confidence}} \cdot (\mathcal{L}_{\text{plddt}} + \mathcal{L}_{\text{pde}} + \mathcal{L}_{\text{resolved}} + \alpha_{\text{pae}} \cdot \mathcal{L}_{\text{pae}}) + \alpha_{\text{diffusion}} \cdot \mathcal{L}_{\text{diffusion}} + \alpha_{\text{distogram}} \cdot \mathcal{L}_{\text{distogram}} \tag{15}$$

Where $\alpha_{\text{confidence}} = 10^{-4}$, $\alpha_{\text{diffusion}} = 4$, $\alpha_{\text{distogram}} = 3 \cdot 10^{-2}$ and $\alpha_{\text{pae}} = 0$ for all except for the final training stage, where it is set to 1.

5.4 Optimization

For training we use the Adam [31] optimizer with parameters $\beta_1 = 0.9$, $\beta_2 = 0.95$, $\epsilon = 10^{-8}$. The base learning rate is $1.8 \cdot 10^{-3}$, which is linearly increased from 0 over the first 1,000 steps. The learning rate is then decreased by a factor of 0.95 every $5 \cdot 10^4$ steps.

The model is trained with a batch size of 256, and we apply gradient clipping if the global norm is greater than 10.

5.5 Dropout

Dropout is applied during training in the MsaModule (Algorithm 8), in the Pairformer stack (Algorithm 17) and in the template embedder (Algorithm 16). See the respective algorithms for the dropout rates.

5.6 Inference Setup

For inference, we use an exponential moving average of the trained parameters [32] with the decay rate of 0.999. Structures are inferenced without any cropping.

5.7 Model selection

For model selection during training stages, performance over a validation set was tracked during training, with all structures in the validation set released after 2021-09-30 and before 2023-01-13 (the maximum release date of training date structures was 2021-09-30). Details of the construction of the validation set are given in subsection 5.8.

A single model selection metric was computed from single chain, interface and full complex scores as follows:

1. Scores were computed for all five samples across all low homology chains and interfaces in the validation set structures, as well as certain full complex metrics.

2. Scores were aggregated across samples via arithmetic mean of top1 (top-ranked) and top5 (best-of-5) predictions, according to global PDE ranking:
$$\mathrm{gPDE} = \frac{\sum_{ij} p_{ij} \mathrm{PDE}_{ij}}{\sum_{ij} p_{ij}}, \tag{16}$$
where the weight p_{ij} is the probability of contact of token pair i, j under the distogram (i.e. the sum of distogram probabilities corresponding to distogram bins under 8 Å).

3. A final scalar was computed via a weighted mean across score types (see Table 7 for weights and description of metrics below), averaged over all targets.

For each interface (chain pair) type and single chain type, LDDTs (local distance difference test) [33] were aggregated. For each pair of low homology chains, interface LDDT was calculated from distances between atoms across different chains in the interface. Intra-chain LDDTs were calculated from distances within a single low homology chain. Nucleic acid LDDTs (intra-chains and interface) were calculated with an inclusion radius of 30 Å compared to the usual 15 Å used for proteins, due to the larger size of nucleotides compared to amino acids. An additional metric, unresolved protein RASA (relative solvent accessible surface area) [34] was calculated for unresolved protein residues in our evaluation set.

Table 7 | Weighting of accuracy metrics for model selection

Metric	Initial training	Fine tuning
Protein-protein interface LDDT	20	20
DNA-protein interface LDDT	10	10
Protein-RNA interface LDDT	10	2
DNA-ligand interface LDDT	5	5
Ligand-protein interface LDDT	10	10
Ligand-RNA interface LDDT	5	2
Protein intra-chain LDDT	20	20
DNA intra-chain LDDT	4	4
RNA intra-chain LDDT	16	16
Ligand intra-chain LDDT	20	20
Modified residue intra-chain LDDT	10	0
Unresolved protein RASA	10	10

5.8 Validation set

The validation set for model selection during training was composed of a all low homology chains and interfaces from a subset of all PDB targets released after 2021-09-30 and before 2023-01-13, with maximum length 2048 tokens.

The process for selecting these targets was broken up into two separate stages. The first was for selecting multimers, the second for selecting monomers. Multimer selection proceeded as follows:

1. Take all targets released after 2021-09-30 and before 2023-01-13 and remove targets with total number of tokens greater than 2560, more than one thousand chains or resolution greater than 4.5, then generate a list of all interface chain pairs for all remaining targets.

2. Filter to only low homology interfaces, which are defined as those where no target in the training set contains two chains with high homology to the chains involved in the interface, where high homology here means $>$ 40% sequence identity for polymers or > 0.85 tanimoto similarity for ligands. Additionally filter out interfaces involving a ligand with ranking model fit less than 0.5 or with multiple residues.

3. Assign interfaces to clusters as per subsubsection 2.5.3, other than for polymer-ligand interfaces which use cluster ID (polymer_cluster, CCD-code) and sample one interface per cluster.

4. Take the following interface types only, possibly reducing number of clusters by sampling a subset of clusters (number of samples given in brackets if reduced): protein-protein (600), protein-DNA (100), DNA-DNA (100), Protein-ligand (600), DNA-ligand (50), ligand-ligand (200), protein-RNA, RNA-RNA, DNA-RNA, RNA-ligand.

5. Take the set of all PDB targets containing the remaining interfaces with a final additional restriction of max total tokens 2048 and make the set of scored chains and interfaces equal to all low homology chains and interfaces in those targets.

6. Manually exclude a small set of targets (11 in our case) where alignment for scoring took too long to be practical for generating validation scores during experiments.

Monomer selection proceeded similarly:

1. Take all polymer monomer targets released after 2021-09-30 and before 2023-01-13 (can include monomer polymers with ligand chains) and remove targets with total number of tokens greater than 2560 or resolution greater than 4.5

2. Filter to only low homology polymers.

3. Assign polymers to clusters as per subsubsection 2.5.3.

4. Sample 40 protein monomers and take all DNA and RNA monomers.

5. Add a final additional restriction of max total tokens 2048 and make the set of scored chains and interfaces equal to all low homology chains and interfaces in the remaining targets.

6. Manually exclude a set of RNA monomers (8 in our case) that all come from one over represented cluster.

The end result was 1,220 PDB targets containing 2,333 low homology interfaces and 2,099 low homology chains.

5.9 Confidence measures and sample ranking

5.9.1 Alignment-based confidence measures

To capture confidence from an alignment perspective we employ pTM and ipTM, PAE-based predictors that have been developed previously [1, 18]. The pTM is a predictor of TM-Score [30], an alignment-based measure of structural accuracy (see Sec. 1.9.7 of [1] for original description). For a given set of tokens $\mathcal{D}$, the pTM is computed as

$$\mathrm{pTM}(\mathcal{D}) = \max_{\substack{i \in \mathcal{D} \\ \text{has\_frame(i)}}} \frac{1}{|\mathcal{D}|} \sum_{j \in \mathcal{D}} \sum_{b=1}^{N_{\text{bins}}} p_{ij}^b \left(\frac{1}{1 + \left(\frac{\Delta_b}{d_0(|\mathcal{D}|)} \right)^2} \right). \tag{17}$$

Here p_{ij}^b is the PAE probability of error bin b for token j aligned to frame i, Δ_b are the associated error bin centers, and N_{bins} is the number of bins (see subsubsection 4.3.2 for PAE description). The has_frame notation indicates that invalid

frames are not considered when computing pTM (see subsubsection 4.3.2 for description of invalid frames). Finally, the factor $d_0(N) = 1.24\sqrt[3]{\text{maximum}(N, 19) - 15} - 1.8$ is the TM-score normalization constant.

With the above equation we can compute multiple confidence measures: for a whole-structure confidence we compute a whole complex pTM, where $\mathcal{D}$ includes all tokens. For single chain confidence we compute a "chain pTM", where $\mathcal{D}$ is restricted to tokens in the chain of interest.

The ipTM (originally described in [18]) is an interface variant of pTM that only considers interactions between different chains,

$$\text{ipTM}(\mathcal{D}) = \max_{\substack{i \in \mathcal{D} \\ \text{has\_frame(i)}}} \frac{1}{|\mathcal{D}_{\text{-chain}(i)}|} \sum_{j \in \mathcal{D}_{\text{-chain}(i)}} \sum_{b=1}^{N_{\text{bins}}} p_{ij}^b \left(\frac{1}{1 + \left(\frac{\Delta_b}{d_0(|\mathcal{D}|)} \right)^2} \right), \tag{18}$$

where $\mathcal{D}_{\text{-chain}(i)}$ is the subset of $\mathcal{D}$ excluding all tokens in the chain of token i. We compute a full structure ipTM by evaluating Equation 18 on a set $\mathcal{D}$ containing all tokens. We also compute a "chain pair ipTM" confidence, where $\mathcal{D}$ is restricted to the set of tokens within a pair of chains defining the interface.

5.9.2 Clashes

We sometimes employ a clash penalty in ranking (see subsubsection 5.9.3), whereby structures with heavily clashing polymer chains are down-weighted. The number of clashes between two chains is defined as

$$\text{clashes(A, B)} = \sum_{i \in A} \sum_{j \in B} d_{ij} < 1.1\,\text{Å}$$

where d_{ij} is the distance between atom i and atom j. A structure is marked as having a clash (has_clash) if for any two polymer chains A, B in the prediction clashes(A, B) > 100 or clashes(A, B)$/\text{min}(N_A, N_B) > 0.5$ where N_A is the number of atoms in chain A. See subsubsection 5.9.3 for how this is used when ranking predictions.

5.9.3 Sample ranking

Sample ranking of the recent PDB evaluation set uses a combination of pTM, ipTM, and pLDDT-based summaries. The ranking depends on whether the metric is single-chain (e.g. protein intra) or an interface (e.g. DNA-protein), as well as on the chain types being scored:

1. Full complex ranking is similar to [18] according to a weighted average of the full-complex pTM and ipTM. We also include terms that penalize predictions with lots of clashes, and very slightly up weight predictions with more disorder (as defined by the relative solvent accessible surface area) to further reduce issues with hallucinations. (subsubsection 5.9.1)

$$0.8 \cdot \text{ipTM} + 0.2 \cdot \text{pTM} + 0.5 \cdot \text{disorder} \; - 100 \cdot \text{has\_clash}. \tag{19}$$

   Where disorder is defined as $\frac{1}{N_P} \sum_{i \in P} (\text{rasa}_i > 0.581)$, P being the set of all protein atoms. The rasa metric, and associated cutoff 0.581 were chosen based on previous work on predicting disorder with AlphaFold [34].

2. Ranking of single-chain metrics (e.g. protein intra) is according to the chain pTM (see subsubsection 5.9.1), where the token subset is restricted to members of the chain.

3. Interface metrics (e.g. protein-protein) are ranked according to a bespoke ipTM aggregate representing the two chains in the interface. We first compute a $[N_{\text{chains}}, N_{\text{chains}}]$ matrix M of chain pair ipTM values (see subsubsection 5.9.1) for all chain pairs in the prediction. For each chain $c \in [A, B]$ in our interface we then compute its average interaction with all other chains: $\mathcal{R}(c) = \underset{ij}{\text{mean}}(M_{ij})$ restricted to $i = c$ or $j = c$, $i \neq j$, and chain i having at least one valid frame. Finally, we rank according to

$$\frac{1}{2} \left[\mathcal{R}(A) + \mathcal{R}(B) \right] \tag{20}$$

   Interfaces containing a small molecule, ion, or bonded ligand chain are ranked differently. Denoting this chain as C^*, the interface is ranked according to $\mathcal{R}(C^*)$ only.

4. Modified residue scores are ranked according to the average pLDDT of the modified residue.

Sample ranking during the model selection phase was performed differently from above. See subsection 5.7 for details.

5.10 Inference Time

Example inference times using 10 trunk recycles on 16 NVIDIA A100 GPUs are shown in Table 8. This includes only GPU wallclock time, and MSA construction, data processing, compilation, and post-processing is not included.

Table 8 | Inference time in seconds by complex size, using 16 A100

Number of tokens	Inference time (seconds)
1024	22
2048	71
3072	126
4096	228
5120	347

6 Evaluation

6.1 Recent PDB evaluation set

The recent PDB evaluation set construction started by taking all 10,192 PDB entries released between 2022-05-01 and 2023-01-12, a date range falling after any data in our training set which had a maximum release date of 2021-09-30. Each entry in the date range was expanded from the asymmetric unit to Biological Assembly 1, then two filters were applied:

- Filtering to non-NMR entries with resolution better than 4.5 Å, leaving 9,636 complexes.
- Filtering to complexes with less than 5,120 tokens under our tokenization scheme (see subsection 2.6), leaving 8,856 complexes.

For each complex we generated a list of all individual entities and a list of all entity pairs where the minimal distance between heavy atoms in the two entities was less than 5 Å and at least one entity in the pair was a polymer. The procedures for determining homology and clusters for these entities and interfaces are described in later sections.

Predictions on the recent PDB set were made on the full post-assembly complex, but crystallization aids (Table 9) were removed from the complex for prediction and scoring, along with all bonds for structures with homomeric subcomplexes lacking the corresponding homomeric symmetry.

Not every entity and interface was included:

- Peptide-peptide interfaces, peptide monomers and modified residues within peptides (where a peptide here is defined as a protein with less than 16 residues) were not included in scoring as their homology to the training set was not determined.
- The system can predict other entities like DNA/RNA hybrids, Peptide Nucleic Acids (PNA) and (D) polypeptides, but these entities and interfaces involving them were not scored as they are too rare to get meaningful results on.
- Eight structures were removed from the test set as matching predicted chains to ground truth chains took too long, due to large numbers of individual entities in the structure.
- Four structures were removed from the set for technical reasons - three where all chains had fewer than 4 residues and one with bad metadata in the source file.
- Eleven ligand-protein or ion-protein interfaces failed to score due to RMSD calculation errors.

In addition to the full evaluation set described in the section above we create a “low homology” subset that is filtered on homology to this training set.

Evaluation is done either on individual chains, or on specific interfaces extracted from the full complex prediction. For intra-chain metrics, we keep polymers that have less than 40% sequence identity to the training set. Here we define sequence identity as the percent of residues in the evaluation set chain that are identical to the training set chain. For interface metrics the following filters are applied:

- Polymer-polymer interfaces: If both polymers have greater than 40% sequence identity to two chains in the same complex in the training set, then this interface is filtered out.
- Peptide-polymer: For interfaces to a peptide (<16 residues), the similarity of the non-peptide entity has to be novel (less than 40% sequence identity to anything in the training set).

6.2 Evaluation set clustering

The evaluation data was clustered to allow for redundancy reduction. Individual polymer chains were clustered at a 40% sequence similarity clustering for proteins with more than 9 residues and 100% similarity for nucleic acids and protein with less than or equal to 9 residues. Ligands, ions, and metal entities were clustered according to CCD identity (only used for **Main Article Fig. 4**). When assigning a cluster ID to an interface:

- Polymer-polymer interfaces are given a cluster ID of (polymer1_cluster, polymer2_cluster).
- Polymer-ligand interfaces are given a cluster ID of the polymer_cluster only.
- Polymer-modified_residue interfaces are given a cluster ID (polymer_cluster, CCD-code).

6.3 Evaluation metrics

The evaluation procedure compares a predicted structure to the corresponding ground truth structure. If the complex contains multiple identical entities, the optimal assignment (maximising LDDT) of the predicted units to the ground truth units is found by either an exhaustive search over all permutations (for groups up to 8 members) or a simulated annealing optimization (for larger groups). After the chain assignment is found, the assignment in local symmetry groups of atoms in ligands is solved by exhaustive search over the first 1000 per-residue symmetries as given by RDKit [25].

We measure the quality of the predictions with DockQ, LDDT (local distance difference test) [33] or pocket-aligned RMSD (root mean square deviation). For nucleic-protein interfaces we measure interface accuracy via interface LDDT (iLDDT), which is calculated from distances between atoms across different chains in the interface. Nucleic acid LDDTs (intra-chains and interface) were calculated with an inclusion radius of 30 Å compared to the usual 15 Å used for proteins, owing to their larger scale.

If not stated differently, the pocket-aligned RMSD is computed as follows: the pocket is defined as all heavy atoms within 10 Å of any heavy atom of the ligand in the ground truth structure. In ligand-protein interfaces the C^{α} atoms within the pocket are used to align the predicted structure to the ground truth structure by least squares rigid alignment, and then RMSD is computed on all heavy atoms of the ligand. In ligand-nucleic chain interfaces all heavy atoms are used for the alignment due to the larger spacing of the backbone atoms and the more rigid structure inside.

We used three categories of evaluation metrics:

1. Complex level metrics (e.g. full complex LDDT score). For these we used the prediction with the highest whole complex confidence over the 5 model seeds.
2. Individual entity metrics (e.g. intra-chain LDDT). For these we used the prediction with the highest entity confidence for the entity being evaluated.
3. Interface metrics (e.g. pocket-aligned RMSD for ligand-protein interfaces). For these we used the prediction with the highest interface confidence for the interface being evaluated.

6.4 Aggregation of scores

To avoid the overrepresentation of similar polymer chains or interfaces that were deposited under different PDB codes, we cluster those together (see subsection 6.2) and aggregate the individual scores such that each cluster gets the same weight. For mean values this is achieved by first computing the mean value per-cluster and then averaging over clusters. For median values and other percentiles we weight each sample by $1/(N_{c_i})$, where N_{c_i} is the size of the cluster the sample belongs to, and compute a weighted percentile.

6.5 Baselines

As part of our ligand accuracy evaluation, we ran docking onto AlphaFold-Multimer v2.3 structures with AutoDock Vina 1.1 [35, 36], using Gypsum-DL [37] and Propka [38] for ligand and protein preparation. The pocket used for docking onto the AlphaFold structures was determined after a rigid alignment with the ground truth structures. We

verified the performance of our docking protocol by recovering the published accuracy on ground truth structures from the PoseBusters set [39]. All other reported baseline numbers in the ligand section are from published literature.

For benchmarking performance on nucleic acid structure prediction, we report baseline comparisons to an existing machine learning system for protein-nucleic acid and RNA tertiary structure prediction, RoseTTAFold2NA [40]. We run the open source RF2NA [41] with the same multiple sequence alignments (MSAs) as were used for AlphaFold 3 predictions. For comparison between AlphaFold 3 and RF2NA, a subset of our recent PDB evaluation set targets are chosen to meet the RF2NA criteria (<1000 total residues and nucleotides). Also as RF2NA was not trained to predict systems with DNA and RNA, this analysis was limited to targets with only one nucleic acid type. Note, that AlphaFold 3 is capable of predicting systems with any combination of protein, DNA, RNA, and ligands.

As an additional baseline for RNA tertiary structure prediction, we evaluate AlphaFold 3 performance on CASP15 RNA targets that are currently publicly available (R1116/8S95, R1117/8FZA, R1126[3], R1128/8BTZ, R1136/7ZJ4, R1138/[7PTK/7PTL], R1189/7YR7, and R1190/7YR6). We compare top-1 ranked predictions, and where multiple ground truth structures exist (R1136) the prediction is scored against the closest state. We display comparisons to RF2NA as a representative machine learning system and AIchemy_RNA2 as the top performing entrant. Both the RF2NA (CASP15 entry BAKER) and AIchemy_RNA2 predictions were downloaded from the CASP website.

Independent work on RoseTTAFold All-Atom [42] was concurrently released that performs structure prediction across a wide range of biomolecular systems. This system is not available for baselining at the time of writing, but the RoseTTAFold All-Atom paper indicates their accuracy is below specialist predictors in almost all categories. The RoseTTAFold All-Atom PoseBusters benchmark score was included since it uses a comparable methodology.

7 Differences to AlphaFold 2 and AlphaFold-Multimer

AlphaFold 3 differs in a number of ways from the previous versions of AlphaFold. A non-exhaustive list of key differences is discussed below.

Centrally, AlphaFold 3 is capable of modeling general molecules; in order to achieve this, we changed the inputs to allow specifying e.g. ligands and modified residues and the way coordinates are represented in the model.

In AlphaFold 2, the protein structure was internally represented by associating a rigid body frame to each amino acid (relating the C^{α} atoms) and the side chains were parameterized by χ-angles. These representations together were used to deduce the coordinates of all the atoms in the amino acid.

That approach was handcrafted for proteins and does not generalize naturally to arbitrary molecules – therefore, in AlphaFold 3 we instead model the system as a collection of atoms, which each have independent global coordinates without any rigid constraints between them. The model learns to respect the chemical structure of the molecules as a result of training, not because the output space is parameterized to enforce it.

In order to allow for efficient computation, we group atoms into tokens. For standard nucleic acids and standard amino acids, tokens correspond to entire residues or nucleotides; for others, each token corresponds to a single heavy atom.

Further, in contrast to AlphaFold 2 and much other recent work, the structure prediction part of the model is not equivariant under spatial transformations. In fact, we employ very limited spatial inductive bias, simply embedding the positions with a single matrix multiplication.

To predict the structure, we employ a generative diffusion-based head, that proceeds by simply adding Gaussian noise to the atom coordinates and training the head to remove the noise, driven by a simple MSE loss. Other than a bond loss to enforce the locations of bonded ligands and glycans (see Equation 5), no violation or clash losses are applied. The relax postprocessing step is rarely needed when using AlphaFold 3.

While we no longer use rigid frames for the core prediction task, we do use frames for the PAE confidence head (subsubsection 4.3.2). This frame construction is largely the same as AlphaFold 2, with exceptions that now we have frames for non-protein entities as well and that the frames are constructed via a different combination of the atom position vectors (compare Algorithm 28 to Algorithm 21 of [1]).

Furthermore, we simplified the core pieces of the architecture. For the main trunk of the model, we moved to a new simplified architecture called “Pairformer” and we removed the MSA representation from the core part of the model and instead replace it with a single representation. As a direct consequence, there is also no column-wise attention.

In Pairformer, we removed the outer product mean that the Evoformer had, meaning there is no information flow from the single to the pair representation. We also simplified the Evoformer stack. The Evoformer stack from AlphaFold 2 operated over both the MSA and residue pairs, and is now replaced with a Pairformer stack that operates over just the token pairs.

[3]Downloaded from https://predictioncenter.org/casp15/TARGETS_PDB/R1126.pdb

In AlphaFold-Multimer, unpaired MSA sequences across chains were presented to the network in a block-diagonal fashion. In AlphaFold 3 these "unpaired" sequences are presented in a dense fashion - so that the network sees many more MSA sequences per-chain.

Lastly, we replaced the ReLU activation in the model's transition blocks function by SwiGLU, which has been observed to yield better results [28]. ReLU is present in the atom attention (Algorithm 5).

When it comes to data, we introduced new distillation sets, and the data gets clustered and re-balanced at the interface level, as opposed to just clustered at the individual chain level. For the protein monomer distillation set, we removed the restrictions on the pLDDT being above 80 and the length of the protein being above 200 residues.

8 Supplemental Results

8.1 Selected examples

In **Main Article Fig. 3** we highlight examples of predicted macromolecular complexes that are now possible with AF3, spanning a range of targets and molecule types, varying in size, complexity, and function.

- Protein-RNA complex: Human 40S ribosomal subunit is a large complex with >7,000 residues, critical to the initiation of human protein synthesis. eIF1A and eIF5B proteins remodel the complex to orient the Met-$\text{tRNA}_\text{i}^\text{Met}$ into a conformation compatible with ribosomal subunit joining. AF3 is able to predict the full $\approx$7,600 residue complex (**Main Article Fig. 3a**). The eIF1A protein interaction with the ribosomal subunit, as well as the eIF5B intra-residue interactions, are modelled correctly. The complex is composed of a set of 43 chains, each of which has high sequence identity to the training set, but represents a novel combination of them as a whole (PDB ID = 7TQL).

- PTM: Proteins harboring heparan sulfate (HS) chains play crucial roles in tissue homeostasis and signal transduction. Exostosin-like 3 (EXTL3) is a glycosyltransferase forming a homocomplex responsible for HS chain synthesis initiation. EXTL3 has 9.4% sequence identity to the training set. AF3 correctly predicts (**Main Article Fig. 3b**) the protein homocomplex and two N-glycans on each of the protomers (bonded glycan accuracy: 0.99 Å mean pocked-aligned RMSD, where the pocket is defined only using residues from the bonded protein chain, PDB ID = 7AU2).

- Antibody-antigen: Mesothelin (MSLN) is a cell-surface protein that is a popular target for antibody-based therapies. A new antibody binding to a section of the juxtamembrane region inhibits MSLN shedding and results in more active CAR-T therapies. No similar sequences could be found for the antigen (residues 584-598 of MSLN), the antibodies have 88% (Fab heavy chain) and 97% (Fab light chain) sequence identity to the training set. AF3 predicts (**Main Article Fig. 3c**) the antibody-antigen interaction to high accuracy (0.82 DockQ, PDB ID = 7U8C).

- Integral membrane protein: Wnt signalling is essential for adult tissue homeostasis, and requires palmitoleoylation by PORCN, an endoplasmic reticulum-resident membrane-bound O-acyltransferase. AF3 correctly predicts (**Main Article Fig. 3d**) the PORCN complex with LGK974 and the WNT3A peptide, providing a structural rationale for the mechanism of action of the clinical stage molecule (PDB ID = 7URD).

- Unique fold: Natural products containing an aziridine ring exhibit antitumor activities. The AziU3/U2 protein complex catalyses the formation of aziridine rings at a novel catalytic site formed by a complex with a novel fold. AF3 correctly predicts (**Main Article Fig. 3e**) this novel protein complex bound to the substrate (PDB ID = 7WUX).

- Allosteric site: PI5P4Kγ is a lipid kinase expressed to regulate cellular levels of the PI5P substrate and the PI(4,5)P2 product, implicated in cancer and immunological disorders. AF3 correctly predicts (**Main Article Fig. 3f**) the novel allosteric binding mode of a novel inhibitor (PDB ID = 7QIE).

In **Extended Data Fig. 6** we considered ligand docking cases studies chosen from the PoseBusters set. Here we compare AF3 ligand pose predictions to the best Vina or Gold docking poses reported in the PoseBusters paper [39], using the pocket-aligned ligand heavy atom RMSD (ligand RMSD). The three examples selected have AF3 ligand RMSD < 2 Å, and the best docking ligand RMSD > 3 Å. We measure ligand Tanimoto similarity using RDKit v.2023_03_3 Morgan fingerprints (radius 2,2048 bits)

- Notum is a serine hydrolase enzyme that catalyses the delipidation of Wnt proteins, and a well-established drug target. While Notum has 99% sequence identity to the training set, the ligand (ARUK3004556) is novel, originally

discovered by a virtual screen, not sharing the Murcko scaffold with any Notum ligand in the PDB, and maximum Tanimoto similarity to any ligand in the training set less than 0.4. AF3 predicts the docked ligand (**Extended Data Fig. 6a**) substantially better than both Gold and Vina docking tools as presented in the PoseBusters benchmark (AF3 ligand RMSD = 1.0 Å, best docking ligand RMSD = 5.2 Å, PDB ID = 8BTI).

- An ethanolamine derivative, HEHEAA, is a potent effector of PipR-mediated gene regulation in plant-associated bacteria. The first step in the response to HEHEAA is the binding to the AapF protein. It is a relatively novel protein (28% sequence identity to the training set), while the maximum Tanimoto similarity of HEHEAA to any ligand in the training set is 0.43. AF3 predicts the docked ligand (**Extended Data Fig. 6b**) substantially better than the best docking algorithm (AF3 ligand RMSD = 1.4 Å, best docking ligand RMSD = 5.4 Å, PDB ID = 7KZ9).

- Galectin-3 is a protein that binds β-galactosides through carbohydrate-recognition domains, and a well-known human drug target. This protein has 91% sequence identity to the training set, and while the ligand (compound 22) shares the core with a known scaffold, the phenyl-1,2,4 triazole side chain is novel. AF3 predicts a more accurate ligand pose (**Extended Data Fig. 6c**) than the best docking algorithm (AF3 ligand RMSD = 0.30 Å, Best docking ligand RMSD = 7.2 Å, PDB ID = 7XFA).

9 Appendix: CCD code and PDB ID tables

Table 9 | Crystallization aids

SO4, GOL, EDO, PO4, ACT, PEG, DMS, TRS, PGE, PG4, FMT, EPE, MPD, MES, CD, IOD

Table 10 | Ligand exclusion list

144, 15P, 1PE, 2F2, 2JC, 3HR, 3SY, 7N5, 7PE, 9JE, AAE, ABA, ACE, ACN, ACT, ACY, AZI, BAM, BCN, BCT, BDN, BEN, BME, BO3, BTB, BTC, BU1, C8E, CAD, CAQ, CBM, CCN, CIT, CL, CLR, CM, CMO, CO3, CPT, CXS, D10, DEP, DIO, DMS, DN, DOD, DOX, EDO, EEE, EGL, EOH, EOX, EPE, ETF, FCY, FJO, FLC, FMT, FW5, GOL, GSH, GTT, GYF, HED, IHP, IHS, IMD, IOD, IPA, IPH, LDA, MB3, MEG, MES, MLA, MLI, MOH, MPD, MRD, MSE, MYR, N, NA, NH2, NH4, NHE, NO3, O4B, OHE, OLA, OLC, OMB, OME, OXA, P6G, PE3, PE4, PEG, PEO, PEP, PG0, PG4, PGE, PGR, PLM, PO4, POL, POP, PVO, SAR, SCN, SEO, SEP, SIN, SO4, SPD, SPM, SR, STE, STO, STU, TAR, TBU, TME, TPO, TRS, UNK, UNL, UNX, UPL, URE

Table 11 | CCD codes defining glycans

045, 05L, 07E, 07Y, 08U, 09X, 0BD, 0H0, 0HX, 0LP, 0MK, 0NZ, 0UB, 0V4, 0WK, 0XY, 0YT, 10M, 12E, 145, 147, 149, 14T, 15L, 16F, 16G, 16O, 17T, 18D, 18O, 1CF, 1FT, 1GL, 1GN, 1LL, 1S3, 1S4, 1SD, 1X4, 20S, 20X, 22O, 22S, 23V, 24S, 25E, 26O, 27C, 289, 291, 293, 2DG, 2DR, 2F8, 2FG, 2FL, 2GL, 2GS, 2H5, 2HA, 2M4, 2M5, 2M8, 2OS, 2WP, 2WS, 32O, 34V, 38J, 3BU, 3DO, 3DY, 3FM, 3GR, 3HD, 3J3, 3J4, 3LJ, 3LR, 3MG, 3MK, 3R3, 3S6, 3SA, 3YW, 40J, 42D, 445, 44S, 46D, 46Z, 475, 48Z, 491, 49A, 49S, 49T, 49V, 4AM, 4CQ, 4GC, 4GL, 4GP, 4JA, 4N2, 4NN, 4QY, 4R1, 4RS, 4SG, 4UZ, 4V5, 50A, 51N, 56N, 57S, 5GF, 5GO, 5II, 5KQ, 5KS, 5KT, 5KV, 5L3, 5LS, 5LT, 5MM, 5N6, 5QP, 5SP, 5TH, 5TJ, 5TK, 5TM, 61J, 62I, 64K, 66O, 6BG, 6C2, 6DM, 6GB, 6GP, 6GR, 6K3, 6KH, 6KL, 6KS, 6KU, 6KW, 6LA, 6LS, 6LW, 6MJ, 6MN, 6PZ, 6S2, 6UD, 6YR, 6ZC, 73E, 79J, 7CV, 7D1, 7GP, 7JZ, 7K2, 7K3, 7NU, 83Y, 89Y, 8B7, 8B9, 8EX, 8GA, 8GG, 8GP, 8I4, 8LR, 8OQ, 8PK, 8S0, 8YV, 95Z, 96O, 98U, 9AM, 9C1, 9CD, 9GP, 9KJ, 9MR, 9OK, 9PG, 9QG, 9S7, 9SG, 9SJ, 9SM, 9SP, 9T1, 9T7, 9VP, 9WJ, 9WN, 9WZ, 9YW, A0K, A1Q, A2G, A5C, A6P, AAL, ABD, ABE, ABF, ABL, AC1, ACR, ACX, ADA, AF1, AFD, AFO, AFP, AGL, AH2, AH8, AHG, AHM, AHR, AIG, ALL, ALX, AMG, AMN, AMU, AMV, ANA, AOG, AQA, ARA, ARB, ARI, ARW, ASC, ASG, ASO, AXP, AXR, AY9, AZC, B0D, B16, B1H, B1N, B2G, B4G, B6D, B7G, B8D, B9D, BBK, BBV, BCD, BDF, BDG, BDP, BDR, BEM, BFN, BG6, BG8, BGC, BGL, BGN, BGP, BGS, BHG, BM3, BM7, BMA, BMX, BND, BNG, BNX, BO1, BOG, BQY, BS7, BTG, BTU, BW3, BWG, BXF, BXP, BXX, BXY, BZD, C3B, C3G, C3X, C4B, C4W, C5X, CBF, CBI, CBK, CDR, CE5, CE6, CE8, CEG, CEZ, CGF, CJB, CKB, CKP, CNP, CR1, CR6, CRA, CT3, CTO, CTR, CTT, D1M, D5E, D6G, DAF, DAG, DAN, DDA, DDL, DEG, DEL, DFR, DFX, DG0, DGO, DGS, DGU, DJB, DJE, DK4, DKX, DKZ, DL6, DLD, DLF, DLG, DNO, DO8, DOM, DPC, DQR, DR2, DR3, DR5, DRI, DSR, DT6, DVC, DYM, E3M, E5G, EAG, EBG, EBQ, EEN, EEQ, EGA, EMP, EMZ, EPG, EQP, EQV, ERE, ERI, ETT, EUS, F1P, F1X, F55, F58, F6P, F8X, FBP, FCA, FCB, FCT, FDP, FDQ, FFC, FFX, FIF, FK9, FKD, FMF, FMO, FNG, FNY, FRU, FSA, FSI, FSM, FSW, FUB, FUC, FUD, FUF, FUL, FUY, FVQ, FX1, FYJ, G0S, G16, G1P, G20, G28, G2F, G3F, G3I, G4D, G4S, G6D, G6P, G6S, G7P, G8Z, GAA, GAC, GAD, GAF, GAL, GAT, GBH, GC1, GC4, GC9, GCB, GCD, GCN, GCO, GCS, GCT, GCU, GCV, GCW, GDA, GDL, GE1, GE3, GFP, GIV, GL0, GL1, GL2, GL4, GL5, GL6, GL7, GL9, GLA, GLC, GLD, GLF, GLG, GLO, GLP, GLS, GLT, GM0, GMB, GMH, GMT, GMZ, GN1, GN4, GNS, GNX, GP0, GP1, GP4, GPH, GPK, GPM, GPO, GPQ, GPU, GPV, GPW, GQ1, GRF, GRX, GS1, GS9, GTK, GTM, GTR, GU0, GU1, GU2, GU3, GU4, GU5, GU6, GU8, GU9, GUF, GUL, GUP, GUZ, GXL, GXV, GYE, GYG, GYP, GYU, GYV, GZL, H1M, H1S, H2P, H3S, H53, H6Q, H6Z, HBZ, HD4, HNV, HNW, HSG, HSH, HSJ, HSQ, HSX, HSY, HTG, HTM, HVC, IAB, IDC, IDF, IDG, IDR, IDS, IDU, IDX, IDY, IEM, IN1, IPT, ISD, ISL, ISX, IXD, J5B, JFZ, JHM, JLT, JRV, JSV, JV4, JVA, JVS, JZR, K5B, K99, KBA, KBG, KD5, KDA, KDB, KDD, KDE, KDF, KDM, KDN, KDO, KDR, KFN, KG1, KGM, KHP, KME, KO1, KO2, KOT, KTU, L0W, L1L, L6S, L6T, LAG, LAH, LAI, LAK, LAO, LAT, LB2, LBS, LBT, LCN, LDY, LEC, LER, LFC, LFR, LGC, LGU, LKA, LKS, LM2, LMO, LNV, LOG, LOX, LRH, LTG, LVO, LVZ, LXB, LXC, LXZ, LZ0, M1F, M1P, M2F, M3M, M3N, M55, M6D, M6P, M7B, M7P, M8C, MA1, MA2, MA3, MA8, MAB, MAF, MAG, MAL, MAN, MAT, MAV, MAW, MBE, MBF, MBG, MCU, MDA, MDP, MFB, MFU, MG5, MGC, MGL, MGS, MJJ, MLB, MLR, MMA, MN0, MNA, MQG, MQT, MRH, MRP, MSX, MTT, MUB, MUR, MVP, MXY, MXZ, MYG, N1L, N3U, N9S, NA1, NAA, NAG, NBG, NBX, NBY, NDG, NFG, NG1, NG6, NGA, NGC, NGE, NGK, NGR, NGS, NGY, NGZ, NHF, NLC, NM6, NM9, NNG, NPF, NSQ, NT1, NTF, NTO, NTP, NXD, NYT, OAK, OI7, OPM, OSU, OTG, OTN, OTU, OX2, P53, P6P, P8E, PA1, PAV, PDX, PH5, PKM, PNA, PNG, PNJ, PNW, PPC, PRP, PSG, PSV, PTQ, PUF, PZU, QDK, QIF, QKH, QPS, QV4, R1P, R1X, R2B, R2G, RAE, RAF, RAM, RAO, RB5, RBL, RCD, RER, RF5, RG1, RGG, RHA, RHC, RI2, RIB, RIP, RM4, RP3, RP5, RP6, RR7, RRJ, RRY, RST, RTG, RTV, RUG, RUU, RV7, RVG, RVM, RWI, RY7, RZM, S7P, S81, SA0, SCG, SCR, SDY, SEJ, SF6, SF9, SFU, SG4, SG5, SG6, SG7, SGA, SGC, SGD, SGN, SHB, SHD, SHG, SIA, SID, SIO, SIZ, SLB, SLM, SLT, SMD, SN5, SNG, SOE, SOG, SOL, SOR, SR1, SSG, SSH, STW, STZ, SUC, SUP, SUS, SWE, SZZ, T68, T6D, T6P, T6T, TA6, TAG, TCB, TDG, TEU, TF0, TFU, TGA, TGK, TGR, TGY, TH1, TM5, TM6, TMR, TMX, TNX, TOA, TOC, TQY, TRE, TRV, TS8, TT7, TTV, TU4, TUG, TUJ, TUP, TUR, TVD, TVG, TVM, TVS, TVV, TVY, TW7, TWA, TWD, TWG, TWJ, TWY, TXB, TYV, U1Y, U2A, U2D, U63, U8V, U97, U9A, U9D, U9G, U9J, U9M, UAP, UBH, UBO, UDC, UEA, V3M, V3P, V71, VG1, VJ1, VJ4, VKN, VTB, W9T, WIA, WOO, WUN, WZ1, WZ2, X0X, X1P, X1X, X2F, X2Y, X34, X6X, X6Y, XDX, XGP, XIL, XKJ, XLF, XLS, XMM, XS2, XXM, XXR, XXX, XYF, XYL, XYP, XYS, XYT, XYZ, YDR, YIO, YJM, YKR, YO5, YX0, YX1, YYB, YYH, YYJ, YYK, YYM, YYQ, YZ0, Z0F, Z15, Z16, Z2D, Z2T, Z3K, Z3L, Z3Q, Z3U, Z4K, Z4R, Z4S, Z4U, Z4V, Z4W, Z4Y, Z57, Z5J, Z5L, Z61, Z6H, Z6J, Z6W, Z8H, Z8T, Z9D, Z9E, Z9H, Z9K, Z9L, Z9M, Z9N, Z9W, ZB0, ZB1, ZB2, ZB3, ZCD, ZCZ, ZD0, ZDC, ZDO, ZEE, ZEL, ZGE, ZMR

Table 12 | Ions

118, 119, 1AL, 1CU, 2FK, 2HP, 2OF, 3CO, 3MT, 3NI, 3OF, 4MO, 4PU, 4TI, 543, 6MO, AG, AL, ALF, AM, ATH, AU, AU3, AUC, BA, BEF, BF4, BO4, BR, BS3, BSY, CA, CAC, CD, CD1, CD3, CD5, CE, CF, CHT, CO, CO5, CON, CR, CS, CSB, CU, CU1, CU2, CU3, CUA, CUZ, CYN, DME, DMI, DSC, DTI, DY, E4N, EDR, EMC, ER3, EU, EU3, F, FE, FE2, FPO, GA, GD3, GEP, HAI, HG, HGC, HO3, IN, IR, IR3, IRI, IUM, K, KO4, LA, LCO, LCP, LI, LU, MAC, MG, MH2, MH3, MMC, MN, MN3, MN5, MN6, MO, MO1, MO2, MO3, MO4, MO5, MO6, MOO, MOS, MOW, MW1, MW2, MW3, NA2, NA5, NA6, NAO, NAW, NET, NI, NI1, NI2, NI3, NO2, NRU, O4M, OAA, OC1, OC2, OC3, OC4, OC5, OC6, OC7, OC8, OCL, OCM, OCN, OCO, OF1, OF2, OF3, OH, OS, OS4, OXL, PB, PBM, PD, PER, PI, PO3, PR, PT, PT4, PTN, RB, RH3, RHD, RU, SB, SE4, SEK, SM, SMO, SO3, T1A, TB, TBA, TCN, TEA, TH, THE, TL, TMA, TRA, V, VN3, VO4, W, WO5, Y1, YB, YB2, YH, YT3, ZCM, ZN, ZN2, ZN3, ZNO, ZO3, ZR

Table 13 | Standard residues

ALA, ARG, ASN, ASP, CYS, GLN, GLU, GLY, HIS, ILE, LEU, LYS, MET, PHE, PRO, SER, THR, TRP, TYR, VAL, UNK, A, G, C, U, DA, DG, DC, DT, N, DN

Table 14 | Recent PDB test set with nucleic acid complexes

7B0C, 7BCA, 7BJQ, 7EDS, 7EOF, 7F3J, 7F8Z, 7F9H, 7M4L, 7MKT, 7MWH, 7MZ0, 7MZ1, 7MZ2, 7N5U, 7N5V, 7N5W, 7NQF, 7NRP, 7OGS, 7OOO, 7OOS, 7OOT, 7OUE, 7OWF, 7OY7, 7OZZ, 7P0W, 7P3F, 7P8L, 7P9J, 7P9Z, 7PSX, 7PTQ, 7PZA, 7PZB, 7Q3O, 7Q4N, 7Q94, 7QAZ, 7QP2, 7R6R, 7R6T, 7R8G, 7R8H, 7R8I, 7RCC, 7RCD, 7RCE, 7RCF, 7RCG, 7RCU, 7RGU, 7RSR, 7RSS, 7S03, 7S68, 7S9J, 7S9K, 7S9L, 7S9M, 7S9N, 7S9O, 7S9P, 7S9Q, 7SOP, 7SOS, 7SOT, 7SOU, 7SOV, 7SOW, 7SUM, 7SUV, 7SVB, 7SX5, 7SXE, 7T18, 7T19, 7T1A, 7T1B, 7T8K, 7TDW, 7TDX, 7TEA, 7TEC, 7TO1, 7TO2, 7TQW, 7TUV, 7TXC, 7TZ1, 7TZR, 7TZS, 7TZT, 7TZU, 7TZV, 7U76, 7U79, 7U7A, 7U7B, 7U7C, 7U7F, 7U7G, 7U7I, 7U7J, 7U7K, 7U7L, 7UBL, 7UBU, 7UCR, 7UPZ, 7UQ6, 7UR5, 7URI, 7URM, 7UU4, 7UXD, 7UZ0, 7V2Z, 7VE5, 7VFT, 7VG8, 7VKI, 7VKL, 7VN2, 7VNV, 7VNW, 7VO9, 7VOU, 7VOV, 7VOX, 7VP1, 7VP2, 7VP3, 7VP4, 7VP5, 7VP7, 7VSJ, 7VTI, 7WM3, 7WQ5, 7X5E, 7X5F, 7X5G, 7X5L, 7X5M, 7XHV, 7XI3, 7XQ5, 7XRC, 7XS4, 7YHO, 7YZE, 7YZF, 7YZG, 7Z0U, 7Z5A, 7ZHH, 7ZVN, 7ZVX, 8A1C, 8A4I, 8AMG, 8AMI, 8AMJ, 8AMK, 8AML, 8AMM, 8AMN, 8B0R, 8CSH, 8CTZ, 8CU0, 8CZQ, 8D28, 8D2A, 8D2B, 8D5L, 8D5O, 8DVP, 8DVR, 8DVS, 8DVU, 8DVY, 8DW0, 8DW1, 8DW4, 8DW8, 8DWM, 8DZK, 8E2P, 8E2Q, 8EDJ, 8EF9, 8EFC, 8EFK, 8GMS, 8GMT, 8GMU

Table 15 | PoseBusters V2 Common Natural Ligands

2BA, 5AD, A3P, ACP, ADP, AKG, ANP, APC, APR, ATP, BCN, BDP, BGC, C5P, CDP, CTP, DGL, DSG, F15, FAD, FDA, FMN, GSH, GSP, GTP, H4B, IPE, MFU, MTA, MTE, NAD, NAI, NCA, NGA, OGA, PGA, PHO, PJ8, PLG, PLP, PRP, SAH, SFG, SIN, SLB, TPP, UD1, UDP, UPG, URI

References

[1] John Jumper, Richard Evans, Alexander Pritzel, Tim Green, Michael Figurnov, Olaf Ronneberger, Kathryn Tunyasuvunakool, Russ Bates, Augustin Žídek, Anna Potapenko, et al. Highly accurate protein structure prediction with AlphaFold. *Nature*, 596(7873):583–589, 2021.

[2] Jimmy Ba, Jamie R Kiros, and Geoffrey E Hinton. Layer Normalization. *arXiv preprint arXiv:1607.06450*, 2016.

[3] H M Berman. The protein data bank. *Nucleic Acids Res.*, 28(1):235–242, January 2000.

[4] Baris E Suzek, Yuqi Wang, Hongzhan Huang, Peter B McGarvey, Cathy H Wu, and UniProt Consortium. Uniref clusters: a comprehensive and scalable alternative for improving sequence similarity searches. *Bioinformatics*, 31(6):926–932, 2015.

[5] L Steven Johnson, Sean R Eddy, and Elon Portugaly. Hidden markov model speed heuristic and iterative hmm search procedure. *BMC Bioinformatics*, 11(1):1–8, 2010.

[6] The UniProt Consortium. Uniprot: the universal protein knowledgebase in 2023. *Nucleic Acids Research*, 51(D1):D523–D531, 2023.

[7] Milot Mirdita, Lars Von Den Driesch, Clovis Galiez, Maria J Martin, Johannes Söding, and Martin Steinegger. Uniclust databases of clustered and deeply annotated protein sequences and alignments. *Nucleic acids research*, 45(D1):D170–D176, 2017.

[8] Martin Steinegger, Milot Mirdita, and Johannes Söding. Protein-level assembly increases protein sequence recovery from metagenomic samples manyfold. *Nature methods*, 16(7):603–606, 2019.

[9] Michael Remmert, Andreas Biegert, Andreas Hauser, and Johannes Söding. Hhblits: lightning-fast iterative protein sequence searching by hmm-hmm alignment. *Nature Methods*, 9(2):173–175, 2012.

[10] Kathryn Tunyasuvunakool, Jonas Adler, Zachary Wu, Tim Green, Michal Zielinski, Augustin Žídek, Alex Bridgland, Andrew Cowie, Clemens Meyer, Agata Laydon, et al. Highly accurate protein structure prediction for the human proteome. *Nature*, 596(7873):590–596, 2021.

[11] Alex L Mitchell, Alexandre Almeida, Martin Beracochea, Miguel Boland, Josephine Burgin, Guy Cochrane, Michael R Crusoe, Varsha Kale, Simon C Potter, Lorna J Richardson, Ekaterina Sakharova, Maxim Scheremetjew, Anton Korobeynikov, Alex Shlemov, Olga Kunyavskaya, Alla Lapidus, and Robert D Finn. MGnify: the microbiome analysis resource in 2020. *Nucleic Acids Research*, 48(D1):D570–D578, 11 2019.

[12] Sean R Eddy. Accelerated profile hmm searches. *PLoS computational biology*, 7(10):e1002195, 2011.

[13] Ioanna Kalvari, Eric P Nawrocki, Nancy Ontiveros-Palacios, Joanna Argasinska, Kevin Lamkiewicz, Manja Marz, Sam Griffiths-Jones, Claire Toffano-Nioche, Daniel Gautheret, Zasha Weinberg, et al. Rfam 14: expanded coverage of metagenomic, viral and microrna families. *Nucleic Acids Research*, 49(D1):D192–D200, 2021.

[14] Martin Steinegger and Johannes Söding. Clustering huge protein sequence sets in linear time. *Nature Communications*, 9(1):1–8, 2018.

[15] Travis J Wheeler and Sean R Eddy. nhmmer: Dna homology search with profile hmms. *Bioinformatics*, 29(19):2487–2489, 2013.

[16] RNAcentral Consortium. Rnacentral 2021: secondary structure integration, improved sequence search and new member databases. *Nucleic acids research*, 49(D1):D212–D220, 2021.

[17] Eric W Sayers, Evan E Bolton, J Rodney Brister, Kathi Canese, Jessica Chan, Donald C Comeau, Catherine M Farrell, Michael Feldgarden, Anna M Fine, Kathryn Funk, et al. Database resources of the national center for biotechnology information in 2023. *Nucleic acids research*, 51(D1):D29–D38, 2023.

[18] Richard Evans, Michael O'Neill, Alexander Pritzel, Natasha Antropova, Andrew Senior, Tim Green, Augustin Žídek, Russ Bates, Sam Blackwell, Jason Yim, et al. Protein complex prediction with AlphaFold-Multimer. *bioRxiv preprint bioRxiv:10.1101/2021.10.04.463034*, 2021.

[19] Timo Lassmann, Oliver Frings, and Erik L L Sonnhammer. Kalign2: high-performance multiple alignment of protein and nucleotide sequences allowing external features. *Nucleic Acids Research*, 37:858–865, 2009.

[20] Jaime A Castro-Mondragon, Rafael Riudavets-Puig, Ieva Rauluseviciute, Roza Berhanu Lemma, Laura Turchi, Romain Blanc-Mathieu, Jeremy Lucas, Paul Boddie, Aziz Khan, Nicolás Manosalva Pérez, et al. Jaspar 2022: the 9th release of the open-access database of transcription factor binding profiles. *Nucleic acids research*, 50(D1):D165–D173, 2022.

[21] Yimeng Yin, Ekaterina Morgunova, Arttu Jolma, Eevi Kaasinen, Biswajyoti Sahu, Syed Khund-Sayeed, Pratyush K Das, Teemu Kivioja, Kashyap Dave, Fan Zhong, et al. Impact of cytosine methylation on dna binding specificities of human transcription factors. *Science*, 356(6337):eaaj2239, 2017.

[22] Arttu Jolma, Yimeng Yin, Kazuhiro R Nitta, Kashyap Dave, Alexander Popov, Minna Taipale, Martin Enge, Teemu Kivioja, Ekaterina Morgunova, and Jussi Taipale. Dna-dependent formation of transcription factor pairs alters their binding specificity. *Nature*, 527(7578):384–388, 2015.

[23] Alexey G Murzin, Steven E Brenner, Tim Hubbard, and Cyrus Chothia. SCOP: a structural classification of proteins database for the investigation of sequences and structures. *Journal of molecular biology*, 247(4):536–540, 1995.

[24] Peter JA Cock, Tiago Antao, Jeffrey T Chang, Brad A Chapman, Cymon J Cox, Andrew Dalke, Iddo Friedberg, Thomas Hamelryck, Frank Kauff, Bartek Wilczynski, et al. Biopython: freely available Python tools for computational molecular biology and bioinformatics. *Bioinformatics*, 25(11):1422, 2009.

[25] Rdkit: Open-source cheminformatics software. `https://www.rdkit.org/`.

[26] Shuzhe Wang, Jagna Witek, Gregory A Landrum, and Sereina Riniker. Improving conformer generation for small rings and macrocycles based on distance geometry and experimental torsional-angle preferences. *Journal of chemical information and modeling*, 60(4):2044–2058, 2020.

[27] William Peebles and Saining Xie. Scalable diffusion models with transformers. *arXiv preprint arXiv:2212.09748*, 2023.

[28] Noam Shazeer. GLU variants improve transformer. *CoRR*, abs/2002.05202, 2020.

[29] Tero Karras, Miika Aittala, Timo Aila, and Samuli Laine. Elucidating the Design Space of Diffusion-Based Generative Models. In S. Koyejo, S. Mohamed, A. Agarwal, D. Belgrave, K. Cho, and A. Oh, editors, *Advances in Neural Information Processing Systems*, volume 35, pages 26565–26577. Curran Associates, Inc., 2022.

[30] Yang Zhang and Jeffrey Skolnick. Scoring function for automated assessment of protein structure template quality. *Proteins: Structure, Function, and Bioinformatics*, 57(4):702–710, 2004.

[31] Diederik P Kingma and Jimmy Ba. Adam: A method for stochastic optimization. In *Proceedings of the International Conference on Learning Representations*, 2015.

[32] B. T. Polyak and A. B. Juditsky. Acceleration of stochastic approximation by averaging. *SIAM Journal on Control and Optimization*, 30(4):838–855, July 1992.

[33] Valerio Mariani, Marco Biasini, Alessandro Barbato, and Torsten Schwede. lddt: A local superposition-free score for comparing protein structures and models using distance difference tests. *Bioinformatics*, 29(21):2722–2728, 2013.

[34] Damiano Piovesan, Alexander Miguel Monzon, and Silvio C. E. Tosatto. Intrinsic protein disorder and conditional folding in AlphaFoldDB. *Protein Science*, 31(11), October 2022.

[35] Jerome Eberhardt, Diogo Santos-Martins, Andreas F. Tillack, and Stefano Forli. Autodock vina 1.2.0: New docking methods, expanded force field, and python bindings. *Journal of Chemical Information and Modeling*, 61(8):3891–3898, Aug 2021.

[36] Oleg Trott and Arthur J. Olson. AutoDock vina: Improving the speed and accuracy of docking with a new scoring function, efficient optimization, and multithreading. *Journal of Computational Chemistry*, 31(2):455–461, June 2009.

[37] Patrick J. Ropp, Jacob O. Spiegel, Jennifer L. Walker, Harrison Green, Guillermo A. Morales, Katherine A. Milliken, John J. Ringe, and Jacob D. Durrant. Gypsum-dl: an open-source program for preparing small-molecule libraries for structure-based virtual screening. *Journal of Cheminformatics*, 11(1):34, May 2019.

[38] Mats H. M. Olsson, Chresten R. Søndergaard, Michal Rostkowski, and Jan H. Jensen. Propka3: Consistent treatment of internal and surface residues in empirical pka predictions. *Journal of Chemical Theory and Computation*, 7(2):525–537, Feb 2011.

[39] Martin Buttenschoen, Garrett M. Morris, and Charlotte M. Deane. Posebusters: AI-based docking methods fail to generate physically valid poses or generalise to novel sequences. *arXiv preprint arXiv:2308.05777*, 2023.

[40] Minkyung Baek, Ryan McHugh, Ivan Anishchenko, David Baker, and Frank DiMaio. Accurate prediction of nucleic acid and protein-nucleic acid complexes using RoseTTAFoldNA. *bioRxiv preprint bioRxiv:10.1101/2022.09.09.507333*, 2022.

[41] Frank DiMaio, Blake Riley, and Alex Morehead. RoseTTAFold2NA, April 2023.

[42] Rohith Krishna, Jue Wang, Woody Ahern, Pascal Sturmfels, Preetham Venkatesh, Indrek Kalvet, Gyu Rie Lee, Felix S. Morey-Burrows, Ivan Anishchenko, Ian R. Humphreys, Ryan McHugh, Dionne Vafeados, Xinting Li, George A. Sutherland, Andrew Hitchcock, C. Neil Hunter, Alex Kang, Evans Brackenbrough, Asim K. Bera, Minkyung Baek, Frank DiMaio, and David Baker. Generalized biomolecular modeling and design with rosettafold all-atom. *Science*, March 2024.
