# Supplementary material for: Accurate structure prediction of biomolecular interactions with AlphaFold 3
Source: Nature. 2024 May 8;630(8016):493–500. doi: 10.1038/s41586-024-07487-w (PMC11168924; doi:10.1038/s41586-024-07487-w)
Supplement: Supplementary file 2 — Reporting Summary [file 41586_2024_7487_MOESM2_ESM.pdf]

nature portfolio

Corresponding author(s): John Jumper (jumper@google.com)

Last updated by author(s): Mar 26, 2024

Reporting Summary

Nature Portfolio wishes to improve the reproducibility of the work that we publish. This form provides structure for consistency and transparency in reporting. For further information on Nature Portfolio policies, see our Editorial Policies and the Editorial Policy Checklist.

Statistics

For all statistical analyses, confirm that the following items are present in the figure legend, table legend, main text, or Methods section.

n/a	Confirmed	
☐	☒	The exact sample size (*n*) for each experimental group/condition, given as a discrete number and unit of measurement
☐	☒	A statement on whether measurements were taken from distinct samples or whether the same sample was measured repeatedly
☐	☒	The statistical test(s) used AND whether they are one- or two-sided<br>*Only common tests should be described solely by name; describe more complex techniques in the Methods section.*
☒	☐	A description of all covariates tested
☐	☒	A description of any assumptions or corrections, such as tests of normality and adjustment for multiple comparisons
☐	☒	A full description of the statistical parameters including central tendency (e.g. means) or other basic estimates (e.g. regression coefficient) AND variation (e.g. standard deviation) or associated estimates of uncertainty (e.g. confidence intervals)
☐	☒	For null hypothesis testing, the test statistic (e.g. *F*, *t*, *r*) with confidence intervals, effect sizes, degrees of freedom and *P* value noted<br>*Give P values as exact values whenever suitable.*
☒	☐	For Bayesian analysis, information on the choice of priors and Markov chain Monte Carlo settings
☒	☐	For hierarchical and complex designs, identification of the appropriate level for tests and full reporting of outcomes
☐	☒	Estimates of effect sizes (e.g. Cohen's *d*, Pearson's *r*), indicating how they were calculated

Our web collection on statistics for biologists contains articles on many of the points above.

Software and code

Policy information about availability of computer code

Data collection	All scientific datasets used to create training and evaluation inputs are freely available from public sources (see Data section below). No additional data was collected.
Data analysis	Data analysis used Python v3.11.7 (https://www.python.org/), NumPy v1.26.3 (https://github.com/numpy/numpy), SciPy v1.9.3 (https://www.scipy.org/), seaborn v0.12.2 (https://github.com/mwaskom/seaborn), Matplotlib v3.6.1 (https://github.com/matplotlib/matplotlib), pandas v2.0.3 (https://github.com/pandas-dev/pandas), statsmodels v0.12.2 (https://github.com/statsmodels/statsmodels), RDKit v4.3.0 (https://github.com/rdkit/rdkit), and Colab (https://research.google.com/colaboratory). TM-align v20190822 (https://zhanglab.dcmb.med.umich.edu/TM-align/) was used for computing TM-scores. Structure visualizations were created in Pymol v2.55.5 (https://github.com/schrodinger/pymol-open-source). PoseBusters scoring done with PoseBusters v0.2.7 (https://github.com/maabuu/posebusters). RoseTTAFold2NA benchmarking done with RoseTTAFold2NA v0.2 (https://github.com/uw-ipd/RoseTTAFold2NA).

For manuscripts utilizing custom algorithms or software that are central to the research but not yet described in published literature, software must be made available to editors and reviewers. We strongly encourage code deposition in a community repository (e.g. GitHub). See the Nature Portfolio guidelines for submitting code & software for further information.

Data

Policy information about availability of data

All manuscripts must include a data availability statement. This statement should provide the following information, where applicable:

- Accession codes, unique identifiers, or web links for publicly available datasets
- A description of any restrictions on data availability
- For clinical datasets or third party data, please ensure that the statement adheres to our policy

All scientific datasets used to create training and evaluation inputs are freely available from public sources. Structures from the PDB were used for training and as templates (https://files.wwpdb.org/pub/pdb/data/assemblies/mmCIF/; for sequence clusters see https://cdn.rcsb.org/resources/sequence/clusters/clusters-by-entity-40.txt; for sequence data see https://files.wwpdb.org/pub/pdb/derived_data/).
Training used a version of the PDB downloaded 12 January 2023, while template search used a version downloaded 28 September 2022. We also used the Chemical Components Dictionary downloaded on 19 October 2023 (https://www.wwpdb.org/data/ccd).

We show experimental structures from the PDB with accession numbers 7PZB50,51, 7PNM52,53, 7TQL54,55, 7AU256,57, 7U8C58,59, 7URD60,61, 7WUX62,63, 7QIE64,65, 7T8266,67, 7CTM68,69, 8CVP43,70, 8D7U43,71, 7F6072,73, 8BTI74,75, 7KZ976,77, 7XFA78,79, 7PEU80,81, 7SDW82,83, 7TNZ84,85, 7R6R 86,87, 7USR88,89, and 7Z1K.90,91

We also used the following publicly available databases for training or evaluation. Detailed usage is described in Supplementary Methods 2.2{Genetic search} and Supplementary Methods 2.5.2{Distillation datasets}.
UniRef90 v.2020_01 (https://ftp.ebi.ac.uk/pub/databases/uniprot/previous_releases/release-2020_01/uniref/),
UniRef90 v.2020_03 (https://ftp.ebi.ac.uk/pub/databases/uniprot/previous_releases/release-2020_03/uniref/),
UniRef90 v.2022_05
https://ftp.ebi.ac.uk/pub/databases/uniprot/previous_releases/release-2022_05/uniref/),
Uniclust30 v.2018_08
(https://wwwuser.gwdg.de/~compbiol/uniclust/2018_08/),
Uniclust30 v.2021_03
(https://wwwuser.gwdg.de/~compbiol/uniclust/2021_03/),
MGnify clusters v.2018_12
(https://ftp.ebi.ac.uk/pub/databases/metagenomics/peptide_database/2018_12/),
MGnify clusters v.2022_05
(https://ftp.ebi.ac.uk/pub/databases/metagenomics/peptide_database/2022_05/),
BFD
(https://bfd.mmseqs.com),
RFam v.14.9
(https://ftp.ebi.ac.uk/pub/databases/Rfam/14.9/),
RNAcentral v.21.0
(https://ftp.ebi.ac.uk/pub/databases/RNAcentral/releases/21.0/),
Nucleotide Database (as of 23 February 2023)
(https://ftp.ncbi.nlm.nih.gov/blast/db/FASTA/nt.gz),
JASPAR 2022
(https://jaspar.elixir.no/downloads/; see https://jaspar.elixir.no/profile-versions for version information),
SELEX protein sequences from Supplementary Tables92
(https://www.ncbi.nlm.nih.gov/pmc/articles/PMC8009048/),
SELEX protein sequences from Supplementary Tables93
(https://www.nature.com/articles/nature15518).

Research involving human participants, their data, or biological material

Policy information about studies with human participants or human data. See also policy information about sex, gender (identity/presentation), and sexual orientation and race, ethnicity and racism.

Reporting on sex and gender	N/A
Reporting on race, ethnicity, or other socially relevant groupings	N/A
Population characteristics	N/A
Recruitment	N/A
Ethics oversight	N/A

Note that full information on the approval of the study protocol must also be provided in the manuscript.

Field-specific reporting

Please select the one below that is the best fit for your research. If you are not sure, read the appropriate sections before making your selection.

☒ Life sciences ☐ Behavioural & social sciences ☐ Ecological, evolutionary & environmental sciences

For a reference copy of the document with all sections, see nature.com/documents/nr-reporting-summary-flat.pdf

Life sciences study design

All studies must disclose on these points even when the disclosure is negative.

Sample size	All available data were used for each benchmark. No subsampling was performed.
Data exclusions	PDB structures were excluded on the basis of size or homology as described in the text
Replication	Code and method details were carefully checked for completeness and replicability.
Randomization	The work constitutes in-silico analysis so all treatments (software packages) were applied to all relevant data for benchmarking.
Blinding	Test sets were held back from training but researchers were not blinded. Large test sizes (all recent PDB) were used instead to avoid overfitting. Fully blind tests would be impractical over the development of the project due to the small size of recent PDB and the need for large samples size on individual new prediction modalities.

Reporting for specific materials, systems and methods

We require information from authors about some types of materials, experimental systems and methods used in many studies. Here, indicate whether each material, system or method listed is relevant to your study. If you are not sure if a list item applies to your research, read the appropriate section before selecting a response.

Materials & experimental systems

n/a	Involved in the study
☒	☐ Antibodies
☒	☐ Eukaryotic cell lines
☒	☐ Palaeontology and archaeology
☒	☐ Animals and other organisms
☒	☐ Clinical data
☒	☐ Dual use research of concern
☒	☐ Plants

Methods

n/a	Involved in the study
☒	☐ ChIP-seq
☒	☐ Flow cytometry
☒	☐ MRI-based neuroimaging

Plants

Seed stocks	*Report on the source of all seed stocks or other plant material used. If applicable, state the seed stock centre and catalogue number. If plant specimens were collected from the field, describe the collection location, date and sampling procedures.*
Novel plant genotypes	*Describe the methods by which all novel plant genotypes were produced. This includes those generated by transgenic approaches, gene editing, chemical/radiation-based mutagenesis and hybridization. For transgenic lines, describe the transformation method, the number of independent lines analyzed and the generation upon which experiments were performed. For gene-edited lines, describe the editor used, the endogenous sequence targeted for editing, the targeting guide RNA sequence (if applicable) and how the editor was applied.*
Authentication	*Describe any authentication procedures for each seed stock used or novel genotype generated. Describe any experiments used to assess the effect of a mutation and, where applicable, how potential secondary effects (e.g. second site T-DNA insertions, mosiacism, off-target gene editing) were examined.*
